# A systematic review of the relationship between subchondral bone features, pain and structural pathology in peripheral joint osteoarthritis

**DOI:** 10.1186/s13075-015-0735-x

**Published:** 2015-08-25

**Authors:** Andrew J. Barr, T. Mark Campbell, Devan Hopkinson, Sarah R. Kingsbury, Mike A. Bowes, Philip G. Conaghan

**Affiliations:** Leeds Institute of Rheumatic and Musculoskeletal Medicine, University of Leeds and NIHR Leeds Musculoskeletal Biomedical Research Unit, Chapeltown Rd, Leeds, LS7 4SA UK; Department of Medicine, University of Ottawa, Ottawa, Canada; Imorphics Ltd, Kilburn House, Manchester, UK

## Abstract

**Introduction:**

Bone is an integral part of the osteoarthritis (OA) process. We conducted a systematic literature review in order to understand the relationship between non-conventional radiographic imaging of subchondral bone, pain, structural pathology and joint replacement in peripheral joint OA.

**Methods:**

A search of the Medline, EMBASE and Cochrane library databases was performed for original articles reporting association between non-conventional radiographic imaging-assessed subchondral bone pathologies and joint replacement, pain or structural progression in knee, hip, hand, ankle and foot OA. Each association was qualitatively characterised by a synthesis of the data from each analysis based upon study design, adequacy of covariate adjustment and quality scoring.

**Results:**

In total 2456 abstracts were screened and 139 papers were included (70 cross-sectional, 71 longitudinal analyses; 116 knee, 15 hip, six hand, two ankle and involved 113 MRI, eight DXA, four CT, eight scintigraphic and eight 2D shape analyses). BMLs, osteophytes and bone shape were independently associated with structural progression or joint replacement. BMLs and bone shape were independently associated with longitudinal change in pain and incident frequent knee pain respectively.

**Conclusion:**

Subchondral bone features have independent associations with structural progression, pain and joint replacement in peripheral OA in the hip and hand but especially in the knee. For peripheral OA sites other than the knee, there are fewer associations and independent associations of bone pathologies with these important OA outcomes which may reflect fewer studies; for example the foot and ankle were poorly studied. Subchondral OA bone appears to be a relevant therapeutic target.

**Systematic review:**

PROSPERO registration number: CRD 42013005009

**Electronic supplementary material:**

The online version of this article (doi:10.1186/s13075-015-0735-x) contains supplementary material, which is available to authorized users.

## Introduction

Osteoarthritis (OA), the most common form of arthritis, is a major cause of chronic pain and disability. OA confers a huge burden on both individuals and health economies [1, 2]. There are currently no licensed disease-modifying osteoarthritis drugs (DMOADs) but ideally these should both inhibit structural progression and improve symptoms and/or function [3, 4]. While hyaline cartilage loss is the hallmark pathology, clinical OA usually involves multiple tissues. Describing the relationships of these tissues with structural progression and symptoms may identify potential tissue targets.

The subchondral bone in particular is intimately associated with hyaline cartilage and therefore a tissue of great potential interest. Conventional radiographs are known to be relatively insensitive to the structural features of OA [5], in part because they do not assess three-dimensional (3D) bone structure [6]. A number of non-conventional radiographic imaging modalities accurately demonstrate in vivo subchondral bone pathological changes, including magnetic resonance imaging (MRI), computed tomography (CT), dual-energy x-ray absorptiometry (DXA), scintigraphy and positron emission tomography (PET) [5, 7–13]. Hunter and colleagues found a moderate association between bone marrow lesions (BMLs), structural progression and longitudinal change in pain in a systematic review focused on MRI biomarkers and knee OA [7]. In another systematic review Kloppenburg and colleagues examined associations between MRI features and knee pain, but not structural pathology [14].

We therefore wished to comprehensively review the literature on subchondral bone structure assessed with all non-conventional radiographic imaging modalities, examining the common sites of peripheral OA and describing the relationships between imaging-detected subchondral bone features and joint replacement, structural progression and pain.

## Methods

### Systematic literature search

**Fig. 1:**
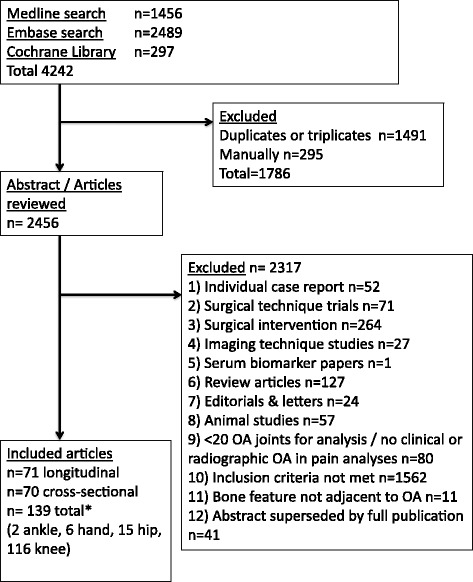
Search strategy results and article exclusion. *Two articles included both cross-sectional and longitudinal data. Longitudinal data included 16 case–control studies and 55 cohort studies

A systematic literature search of Medline (from 1950), EMBASE (from 1980) and the Cochrane library databases until September 2014 was performed. A full description of the search terms used is recorded in Additional file 1: Table S1. An abbreviation of the full search terms used was ‘knee, hip, hand, foot and ankle’ and ‘osteoarthritis’ and ‘subchondral bone’ manifestations of OA (‘bone marrow lesion’, ‘osteophyte’, ‘bone cyst’, ‘bone area’, ‘bone shape’, ‘bone attrition’, bone morphometry and mineral density) and ‘MRI’ or ‘CT’ or ‘DXA’ or ‘scintigraphy’ or ‘PET’. The search term ‘bone shape’ was not restricted to non-conventional radiographic imaging. The final search was restricted to humans. There was no language restriction and abstracts were not excluded. Exclusion criteria are listed in Fig. 1. Any analysis of fewer than 20 patients with confirmed OA was excluded to remove papers at risk of study imprecision. The inclusion criteria were in vivo observational studies of a human population with clinical and/or radiographic OA, which included an imaging description of the adjacent subchondral bone pathology to the osteoarthritic joint and the relationship of this with pain, structural progression or joint replacement. Analyses describing the relationship between OA bone manifestations and structural severity (cross-sectional) or progression (prospective cohorts) in populations without clinical and radiographic OA were included to incorporate early structural features of joint degeneration. The outcome measures of structural severity or progression included cartilage defects, cartilage thickness, cartilage volume, denuded subchondral bone, Kellgren-Lawrence (KL)grade, joint space width and joint space narrowing. Other outcome measures included joint replacement and any pain measures.

The articles identified by the preliminary search were screened by two reviewers (DH, AB) for relevance and for references not identified by the preliminary search, although no additional citations were found. Discordance in opinion was resolved by a third reviewer (SK). We applied the methods for reporting meta-analyses of observational studies in epidemiology that are recommended by the Cochrane collaboration [15, 16].

### Data extraction

Data extraction was performed by two reviewers (DH, AB) as described in the Supplementary methods ‘data extraction’ (see Additional file 1).

### Quality assessment

The quality of each observational study was independently assessed by two reviewers (TC, AB), as described in Supplementary methods ‘Quality assessment’ (see Additional file 1).

### Best evidence synthesis

Statistical pooling of the data was considered inappropriate in light of the heterogeneous study populations, methodological quality and bone feature or outcome measurements for OA. Therefore a qualitative summary of the evidence for each bone feature (e.g., BML) and its association with pain or structural progression and joint replacement was provided based on the study design, adequacy of adjustment for confounders (age, body mass index and gender) and quality score as described in the Supplementary methods ‘Best evidence synthesis’ (see Additional file 1).

Studies that investigated the association between multiple bone features and OA pain or structural progression outcomes were considered as a single study for each bone feature. Included studies that established significant correlation between bone and pain, structural progression or joint replacement were described as positive (+) or negative (−) accordingly. If no association or inconclusive findings were described this was reported as no association (NA) or no conclusion (NC) respectively.

## Results

### Systematic literature search and selection

The Preferred reporting items for systematic reviews and meta-analyses (PRISMA) diagram in Fig. 1 describes the literature flow. Following exclusion of duplicates and triplicates, 2,456 articles met the search criteria. After applying inclusion/exclusion criteria, 139 articles were included for data extraction and quality scoring. In total, 71 papers provided longitudinal data (55 cohorts, 16 case−controls), 70 provided cross-sectional data, and two papers provided both.

### Data extraction from selected studies

In only 12 studies did the mean age fall below 50 years [17–29]. Most (n = 93) described both genders; 2 studies included men only [27, 30], 14 studies included female individuals only [22, 28, 29, 31–41] and there was an undisclosed gender ratio in 6 [42–50]. Knee OA was defined using clinical and radiographic criteria and is described in Additional file 1: Table S6. Radiographic OA was invariably defined as KL grade ≥2 or any radiographic OA abnormality from the Altman atlas [51]. Individual pain or structural progression measures were examined in 88 studies; 52 studies examined multiple features. Subchondral bone was analyzed with MRI in 113 articles, DXA in 8 [30–32, 42, 52–55], CT in 4 [33, 40, 56, 57], and scintigraphy in 8 [24, 37, 38, 58–62], and no articles using PET met the inclusion criteria. Included articles described 116 knee, 15 hip, 6 hand and 2 ankle studies. Of these studies 13 described structural associations without clinical or radiographic OA [18, 19, 23, 25, 26, 35, 63–69]. There were no articles on studies of the foot that met the inclusion criteria.

### Quality assessment of studies

Concordance of opinion in quality scoring was observed in 2,040 (89 %) of the 2,242 scoring items assessed, which are recorded in Additional file 1: Tables S3-S5. The majority of discordant scoring was for study design (criteria 17) and data presentation (criteria 18). Quality scores were converted to percentages of the maximum scores for each class of paper. The mean (range) quality score was 59 % (29–79), 54 % (22–83) and 59 % (47–76) for cross-sectional, cohort and case–control studies, respectively.

### Relationship between knee bone feature and structural progression

The association of bone features with structural progression and joint replacement are described in Tables 1 and 5.

**Table 1:** Knee structural associations by feature and quality grade

Author	Feature (method)	Structural progression outcome	Adjustment for confounders	Association (magnitude) crude	Association (magnitude) adjusted	Association	Quality (score %)
MRI bone marrow lesion - cohorts							
Felson 2003 [70]	Baseline presence of BML in medial or lateral TFJ (C)	OARSI JSN grade progression of TFJ (L)	Age, sex, and BMI	NR	OR 6.5,	+	High (83)
					95 % CI 3.0 to 14.0		
Dore 2010 [124]	Baseline semi-quantitative MRI BML size (C) TFJ	Incident TKR over 5 years (L)	Age, sex, BMI, knee baseline pain, leg strength, cartilage defects, tibial bone area, ROA	OR (95 % CI)	OR (95 % CI)	+	High (64)
				2.04 (1.55 to 2.69)	2.10 (1.13 to 3.90)		
				*p* <0.01	*p* = 0.019		
Driban 2013 [72]	Knee baseline BML volume (C)	48-month change in OARSI JSN grade (L)	Age, sex, BMI	NR	Baseline BML volume	+	High (61)
					OR 1.27, 95 % CI 1.11 to 1.46		
	BML volume 48 month change (L) (TFJ)	(TFJ)			BML volume regression		
					OR 3.36, 95 % CI 1.55 to 7.28		
Davies-Tuck 2010 [67]	Incident BML (new BML after 2 years with no BMLs at baseline) MRI TFJ (L)	Progression in semi-quantitative MRI cartilage defects score after 2 years. TFJ (L)	Age, gender, BMI, baseline cartilage volume	OR (95 % CI)	OR (95 % CI)	+	High (61)
				Medial TFJ	Medial TFJ	Association in the lateral TFJ and a trend in the medial TFJ	
				1.86 (0.70 to 4.93) *p* = 0.21	2.63 (0.93 to 7.44) *p* = 0.07		
				Lateral TFJ	Lateral TFJ		
				3.0 (1.01 to 8.93) *p* = 0.05	3.13 (1.01 to 9.68) *p* = 0.05		
Hochberg 2014 [44]	Semi-quantitative MRI baseline femoral condyle BML size (C)	Incident TKR over 6 years (L)	Age, gender, BMI, race, marital status, depressive symptoms, quality of life, mechanical pain, KL grade, clinical effusion.	Medial TFJ	Medial TFJ	+	High (61)
				*p* <0.0001	*p* = 0.02		
Raynauld 2011 [75]	Baseline semi-quantitative BML score (C) TFJ	Incidence of TKR over 3 years (L)	Age, sex, BMI, JSW, WOMAC,	NR	OR (95% CI)	+	High (61)
					BML medial plateau		
					1.81 (1.08 to 2.03)		
					*p* = 0.025		
Raynauld 2013 [74]	Baseline semi-quantitative BML WORMS score (C) medial TFJ	Incident TKR (L) 4 year follow up	Age, BMI, gender WOMAC, CRP	NR	TKR incidence	+	High (61)
					OR (95 % CI) 2.107 (1.26 to 3.54) *p* = 0.005 time to TKR incidence hazard ratio (95% CI) 2.13 (1.38 to 3.30) *p* = 0.001		
		Time to TKR (L)					
Crema 2014 [71]	MRI BML (semi-quantitative)	Cartilage loss (semi-quantitative)	Age, gender, BMI	NR	*β* = 0.37 to 0.64 *p* <0.001	+	High (56)
	(C) all regions	(L) (all regions)					
Guermazi 2014 Abstract [73]	Baseline semi-quantitative BML score WORMS (C)	Cartilage thickness loss over 30 months (L)	Age, sex, body mass index, and anatomical alignment axis (degrees)	NR	Combined BML score in the medial and lateral TFJ compartment	+	High (56)
					OR 1.9, 95 % CI 1.1 to 3.3		
Scher 2008 [87]	Presence of any baseline semi-quantitative MRI BMLs (C)	Incident TKR (L) over 3 years	Age	NR	OR (95 % CI)	+	High (56)
					8.95 (1.49 to 53.68)		
					*p* = 0.02		
Sowers 2011 [28]	Semi-quantitative MRI BML, size in TFJ (C)	Progression in KL grade	Nil	*R* (95 % CI) medial tibia ~ 0.46 (0.35 to 0.55)	NR	+	Low (53)
		(11-year follow up) (L)		Lateral tibia ~0.23 (0.13 to 0.33)			
Kothari 2010 [82]	Semi-quantitative baseline MRI BML, (WORMS) (C) TFJ	Semi-quantitative cartilage defect score change over 2 years (WORMS) (L) TFJ.	Age, sex, BMI, other bone lesions	OR 4.04,	OR 3.75,	+	Low (50)
				95 % CI 2.25 to 7.26	95 % CI 1.59 to 8.82		
Raynauld 2008 [85]	Change in BML size (mm) at 24 months in medial TFJ (L)	Medial cartilage volume (L) at 24 months in medial TFJ	Age, gender, BMI, meniscal extrusion and tear, pain and bone lesions at baseline	NR	Change in BML size with femoral cartilage volume loss	-	Low (50)
						Larger medial BML size means more cartilage loss in medial compartment	
					*β* = −0.31		
					standard error (0.08)		
					*p* = 0.0004		
Roemer 2009 [90]	Change in MRI semi-quantitative BML size (WORMS) (L) TFJ and PFJ	Progression in semi-quantitative cartilage defects in (WORMS) over 30 months (L) TFJ and PFJ	Age, sex, BMI, baseline KL grade	NR	OR (95 % CI)	+	Low (50)
					Incident BML OR 3.5 (2.1 to 5.9)		
					Progression of BML 2.8 (1.5 to 3.2)		
					Resolution of BML OR 0.9 (0.5 to 1.6)		
					Stable BML OR 1.0 (reference)		
Dore 2010 [76]	Baseline semi-quantitative BML severity (C) (medial and lateral TFJ)	Ipsi-compartmental annual Cartilage volume loss (L)	Age, sex, BMI, meniscal damage	NR	Baseline	-	Low (50)
					BML severity	Bigger BML means bigger volume loss	
					*β* = −22.1 to −42.0, for all regions		
					(*p* <0.05)		
Parsons 2014 Abstract [83]	Baseline semi-quantitative BML score (C)	Annual TFJ JSN (L)	Age, sex, baseline KL grade	NR	*β* = −0.10, 95 % CI	+	Low (50)
					−0.18 to		
					−0.02		
Wildi 2010 [95]	24-month regional change in TFJ BML score WORMS (L)	24-month regional change in cartilage volume (L)	nil	*R* correlation coefficients all <0.07	NR	NC	Low (50)
				*p* >0.367 for all three compartments at 24 months			
Pelletier 2007 [84]	Regional Semi-quantitative baseline BML score (medial or lateral TFJ) (C)	Regional cartilage volume over 24 months (medial or lateral TFJ) (L)	NR	Lateral compartment BML score	NR		Low (50)
				*β* = −0.31, *p* = 0.001			
Driban 2011 [79]	Baseline BML volume (C) and 24 month change in BML volume (L) in TFJ compartments	24-month change in full thickness cartilage lesion area (L)	Age, sex, body mass index	NR	Baseline BML volume *r* = 0.48, 95 % CI 0.20 to 0.69	+	Low (50)
						Baseline femur BML volume with loss in ipsicompartmental full thickness cartilage lesion area.	
					*p* <0.002		
Tanamas 2010 [89]	Baseline semi-quantitative MRI BML size (C) TFJ	Cartilage volume change over 2 years (L) TFJ Incident TKR over 4 years	Age, sex, BMI, baseline tibial cartilage volume and bone area	*R* (95 % CI)	*R* (95 % CI)	+	Low (50)
				Total cartilage loss	Total cartilage loss		
				0.61 (−0.11 to 1.33)	1.09 (0.24, 1.93)		
				OR (95 % CI)	OR (95 % CI)		
				Incident TKR	Incident TKR		
				1.55 (1.04 to 2.29)	1.57 (1.04 to 2.35)		
				*p* = 0.03	*p* = 0.03		
Madan-Sharma 2008 [93]	Baseline MRI semi-quantitative BML (C) TFJ	OARSI medial TFJ JSN grade progression over 2 years (L) TFJ	Age, sex, BMI, family effect	NR	0.9 RR,	NA	Low (47)
					95 % CI 0.18 to 3.0		
Tanamas 2010 [88]	Semi-quantitative change in MRI BML severity (C)	Incident TKR over 4 years (L)	Age, gender, KL grade	OR (95 % CI)	OR (95 % CI)	+	Low (47)
				Medial TFJ	Medial TFJ	Association in the medial TFJ but not in the lateral TFJ	
				1.72	1.99		
				(0.93 to 3.18)	(1.01 to 3.90)		
				*p* = 0.08	*p* = 0.05		
				Lateral TFJ	Lateral TFJ		
				0.95 (0.48 to 1.88)	0.96 (0.48 to 1.94)		
				*p* = 0.89	*p* = 0.91		
Roemer 2012 [86]	Semi-quantitative BML (WORMS) TFJ and PFJ (C)	Semi-quantitative cartilage score 6-month progression TFJ and PFJ (L)	Age, sex, treatment, and BMI.	NR	BML TFJ OR 4.74, 95 % CI 1.14 to 19.5	+	Low (44)
					*p* = 0.032	BMLs and cartilage score correlate	
					BML PFJ OR, 1.63 (0.67 to 3.92)		
Crema 2013 [78]	MRI incident BML (WORMS)	Progressive (30 month) semi-quantitative cartilage defect (WORMS) TFJ (L)	Age, sex, BMI, malalignment, meniscal disease	NR	OR (95 % CI)	+	Low (44)
	TFJ				Medial TFJ 7.6		
	(L)				(5.1 to 11.3)		
					Lateral TFJ		
					11.9 (6.2 to 23.0)		
Hernandez-Molina 2008 [81]	Crude presence of central BMLs on MRI (C) TFJ	Semi-quantitative cartilage defect (WORMS) (L) TFJ	Alignment, BMI, KL grade, sex, and age.	NR	Medial TFJ cartilage loss	+	Low (44)
					OR 6.1,		
					95 % CI 1.0, 35.2		
Koster 2011 [25]	Baseline BML presence (C) TFJ	Any progression in KL grade over 1 year (L) TFJ	Age, BMI	OR (95 % CI)	OR (95 % CI)	+	Low (44)
				6.01 (1.92 to 18.8)	5.29 (1.64 to 17.1)		
				*p* = 0.002	*p* = 0.005		
Hunter 2006 [91]	Change in MRI semi-quantitative BML score (L) TFJ	Change in semi-quantitative cartilage defect score (WORMS) (L) medial or lateral TFJ	Limb alignment	Ipsilateral cartilage loss	Ipsilateral cartilage loss	NA after adjustment	Low (44)
				*β* = 0.65	*β* = 0.26		
				*p* = 0.003	*p* = 0.16		
				Contralateral cartilage loss	Contralateral cartilage loss		
				*β* = −0.27	*β* = −0.16		
				*p* = 0.22	*p* = 0.52		
Roemer 2009 [94]	Baseline MRI BML crude presence or absence (WORMS) (L) TFJ	Semi-quantitative cartilage defect progression over 30 months (WORMS) (L) TFJ	Age, sex, race, BMI, alignment	OR (95 % CI)	OR (95 % CI)	NA	Low (44)
				Slow cartilage loss OR 1.74 (0.85 to 3.55)	Slow cartilage loss OR 1.79 (0.83 to 3.87)		
				Fast cartilage loss OR 1.32 (0.37 to 4.78)	Fast cartilage loss OR 1.0 (0.24 to 4.10)		
Kubota 2010 [92]	MRI BML semi-quantitative volume score change over 6 months (L) TFJ	KL grade progression over 6 months (L) TFJ	Nil	BML score higher in KL progression group	NR	NC	Low (39)
				*p* = 0.044			
Driban 2012 abstract [80]	MRI BML volume change (L) TFJ over 24 months	Change in cartilage thickness and denuded area of bone (L) TFJ over 24 months	Nil	Cartilage thickness	NR	+	Low (28)
				*r* = −0.34, *p* = 0.04			
				denuded bone			
				*r* = 0.42, *p* = 0.01			
				Femoral cartilage indices *p* >0.05			
Carrino 2006 [77]	Crude presence of MRI BML, TFJ (C) and (L)	Any grade of cartilage defect TFJ (C) and (L)	Nil	NR	NR	+	Low (22)
MRI bone marrow lesion - cross-sectional studies							
Baranyay 2007 [63]	MRI BML defined as large or not large/absent in the medial and lateral compartments of TFJ (C)	MRI semi-quantitative cartilage defects of medial and lateral compartments of TFJ (C)	Age, gender, BMI, cartilage volume or bone area	OR (95 % CI)	OR (95 % CI)	+	High (71 %)
		Quantitative cartilage volume medial and lateral TFJ (C)		Cartilage defect Medial TFJ	Cartilage defect Medial TFJ	Cartilage defects	
				1.81 (1.26 to 2.59) *p* = 0.005	1.80 (1.21 to 2.69) *p* = 0.004	NA	
				Lateral TFJ	Lateral TFJ	Cartilage volume	
				1.52 (1.14 to 2.04)	1.45 (1.02 to 2.07)		
				*p* = 0.005	*p* = 0.04		
				No association with ipsicompartmental cartilage volume	No association with ipsicompartmental cartilage volume		
Guymer 2007 [35]	Presence or absence of MRI BMLs	Presence or absence of semi-quantitative cartilage defects	Age, height, weight, and tibial cartilage volume	OR (95 % CI)	OR (95 % CI)	+	High (71)
	(C) TFJ	(C) TFJ		Medial TFJ	Medial TFJ	A positive association is observed in the medial but not the lateral TFJ	
				6.46 (1.04 to 38.39)	3.51 (1.08 to 11.42)		
				*p* = 0.04	*p* = 0.04		
				Lateral TFJ	Lateral TFJ		
				1.17 (0.22 to 6.26)	1.02 (0.17 to 6.12)		
				*p* = 0.85	*p* = 0.98		
Stehling 2010 [65]	Presence of any MRI semi-quantitative BMLs (C)	Presence of any WORMS MRI cartilage defects (C)	Age, gender and BMI, KL score, knee injury or knee surgery, family history of TKR and Heberden's nodes	NR	*p* <0.0001	+	High (71)
Torres 2006 [103]	MRI BML (WORMS) (C) TFJ and PFJ	Semi-quantitative cartilage (WORMS) (C)	Nil	*R* = 0.56	NR	+	High (68)
Ip 2011 [99]	Semi-quantitative MRI BML (C)	KL grade (C)	Age, sex, BMI, OA stage, joint effusion, and meniscal damage	NR	Highest BML score *p* <0.001	+	High (68)
Hayes 2005 [22]	Semi-quantitative MRI BML (C)	KL grade (C)	Nil	*p* = 0.005	NR	+	High (61)
Kornaat 2005 [100]	Semi-quantitative MRI BML (KOSS)	Semi-quantitative cartilage defects (KOSS) TFJ and PFJ (C)	Nil	OR (95 % CI)	NR	+	Low (57)
				PFJ			
	TFJ and PFJ (C)			17 (3.8 to 72)			
				TFJ			
				120 (6.5 to 2,221)			
Gudbergsen 2013 [98]	Semi-quantitative MRI BML (BLOKS) (C)	KL grade (C)	Nil	KL grade	NR	+	Low (57)
				*p* = 0.046 lateral			
				*p* <0.001 medial			
Link 2003 [101]	Semi-quantitative MRI BML, (C)	KL grade (C)	Nil	*p* <0.05	NR	+	Low (54)
Sowers 2003 [29]	Semi-quantitative MRI BML (C)	Semi-quantitative cartilage defect (C)	Nil	*p* for trend	NR	+	Low (54)
				*p* <0.0001			
Felson 2001 [96]	Semi-quantitative MRI BMLs (C)	KL grade (C)	Nil	NR	NR	+	Low (54)
Lo 2005 [102]	Semi-quantitative MRI BML (WORMS ≥ 1) (C)	KL grade ≥ 2 (C)	Nil	NR	NR	+	Low (50)
Meredith 2009 [64]	Sum of semi-quantitative MRI	Sum of semi-quantitative MRI	Nil	*p* <0.0003	NR	+	Low (50)
	BML scores in the TFJ and PFJ (C)	Cartilage defect scores in the TFJ and PFJ (C)					
Fernandez-Madrid 1994 [97]	Crude presence of MRI BMLs (C)	KL grade (C)	Nil	*p* <0.001	NR	+	Low (46)
Scher 2008 [87]	Semi-quantitative MRI BML (C)	Semi-quantitative cartilage defect (modified Noyes) (C)	Nil	*p* = 0.012	NR	+	Low (43)
MRI bone marrow lesion - case control studies							
Ratzlaff 2014 [104]	Total tibial BML volume 12 and 24 months before TKR and interval change between 12 and 24 (C) and (L) TFJ	Incident TKR (L)	NB matched cases and controls	OR (95 % CI)	NR	+	High (65)
				12 months (C)		True of TFJ but not PFJ	
				1.68 (1.33 to 2.13)			
				24 months (C)			
				1.35 (1.02 to 1.78)			
				12 to 24 months change (L)			
				1.23 (1.03 to 1.46)			
Zhao 2010 [105]	Baseline crude presence of MRI BMLs at (C) TFJ	Overlying cartilage defect progression after 1 year (WORMS) (L) TFJ	Nil	Change in cartilage defect scores for areas with and without underlying BMLs	NR	+	Low (56)
				*p* = 0.00003			
Aitken 2013 Abstract [17]	Semi-quantitative BMLs tibia, femur and patella	Cartilage volume and defect score tibia and femur	Age, sex, BMI	NR	Tibial cartilage volume	-	Low (47)
					*β* = −433 mm^3^ per unit increase in BML		
					*p* <0.01		
Stahl 2011 [41]	Semi-quantitative MRI BML size (WORMS) (L) TFJ	Semi-quantitative cartilage defect size (L) TFJ	Nil	NR	*p* <0.165	NA	Low (47)
MRI osteophyte - cohort studies							
De-Lange 2014 abstract [106]	Semi-quantitative osteophyte (KOSS) (C)	Radiographic progression of JSN of TFJ (L)	Age, gender, BMI and baseline JSN	NR	OR (95 % CI)	+	High (61)
					1.8 (1.1 to 3.1)	Higher OST score, the higher the JSN	
Liu 2014 Abstract [45]	Baseline semi-quantitative osteophyte score (WORMS) (C) TFJ	Incident TKR at 6-months follow up (L)	Activity of daily living disability score	NR	RR (95 % CI) 3.01 (1.39 to 6.52)	+	Low (50)
Sowers 2011 [28]	Semi-quantitative MRI osteophyte size in TFJ (C)	Progression in KL grade (11-year follow up) (L)	Nil	*R* (95 % CI) medial tibia ~ 0.65 (0.59 to 0.71)	NR	+	Low (53)
				Lateral tibia ~0.57 (0.49 to 0.63)			
MRI osteophyte - cross-sectional studies							
Stehling 2010 [65]	Presence of any MRI semi-quantitative osteophytes (C)	Presence of any WORMS MRI cartilage defects (C)	Age, gender and BMI, KL score, knee injury or knee surgery, family history of TKR and Heberden’s nodes	NR	*p* = 0.0037	+	High (71)
Torres 2006 [103]	MRI osteophyte, (WORMS) TFJ and PFJ (C)	Semi-quantitative cartilage (WORMS) TFJ and PFJ (C)	Nil	*R* = 0.73	NR	+	High (68)
Hayes 2005 [22]	Semi-quantitative MRI osteophyte (C)	KL grade (C)	Nil	*p* <0.001	NR	+	High (61)
Meredith 2009 [64]	Sum of semi-quantitative MRI	Sum of semi-quantitative MRI	Nil	*p* <0.0001	NR	+	Low (50)
	Osteophyte scores in the TFJ and PFJ (C)	cartilage defect scores in the TFJ and PFJ (C)					
McCauley 2001 [26]	MRI central osteophyte presence (C) TFJ	MRI cartilage lesion presence (C) TFJ	Nil	Crude association of 32 of 35 central osteophytes having adjacent cartilage lesions	NR	+	Low (29)
						Crude, unadjusted	
Roemer 2012 [108]	MRI osteophyte	Cartilage defect (WORMS) (C)	Age, sex, BMI, race, TFJ radiographic OA	OR 2378.1,	OR 108.8,	+	Low (57)
				95 % CI 249.8 to 22643.4	95 % CI 14.2 to 834.9		
	(WORMS) (C)						
					*p* for trend <0.0001		
Link 2003 [101]	Semi-quantitative MRI osteophytes (C)	KL grade (C)	Nil	*p* <0.01	NR	+	Low (54)
Fernandez-Madrid 1994 [97]	Crude presence of MRI osteophytes (C)	KL grade (C)	Nil	*p* <0.001	NR	+	Low (46)
MRI bone attrition - cohort studies							
Kothari 2010 [82]	Semi-quantitative baseline MRI attrition	Semi-quantitative cartilage defect score change over 2 years (WORMS) (L) TFJ.	Age, sex BMI, other bone lesions	OR 3.17,	OR 1.85,	NA	Low (50)
				95 % CI 1.64 to 6.16	95 % CI 0.71 to 4.82		
	(WORMS) (C) TFJ						
MRI bone attrition - cross-sectional studies							
Torres 2006 [103]	MRI attrition (WORMS) TFJ and PFJ (C)	Semi-quantitative cartilage (WORMS) TFJ and PFJ (C)	Nil	*R* = 0.75	NR	+	High (68)
Reichenbach 2008 [110]	Semi-quantitative MRI bone attrition (WORMS) (C)	KL grade and semi-quantitative cartilage defects (WORMS) (C)	Nil	NR	NR	+	Low (43)
				Crude correlation			
MRI bone attrition - case control studies							
Neogi 2009 [109]	Baseline semi-quantitative MRI bone attrition size (WORMS) (C) TFJ	Cartilage defects progression (WORMS) after 30 months TFJ	Age, sex, BMI	OR 5.5,	OR 3.0,	+	Low (59)
				95 % CI 3.0 to 10.0	95 % CI 2.2 to 4.2		
MRI bone Shape/dimension – cohort studies							
Cicuttini 2004 [111]	Baseline quantitative MRI tibial bone area (C)	TKR incidence (L) over 4 years	Age, sex, height, weight, BMI, WOMAC, ROA severity	NR	OR (95 % CI)	+	High (78)
					1.2 (1.0 to 1.4)		
					*p* = 0.02		
Ding 2008 [20]	Baseline MRI tibial bone area (C) TFJ	Progressive cartilage volume loss (L) TFJ	Age, sex, BMI, OA family history, muscle strength and ROA.	*β* (95 % CI)	*β* (95 % CI)	-	High (72)
				Medial femoral cartilage	Medial femoral cartilage		
				*β* = 0.17 (0.04 to 0.29)	*β* = 0.35 (0.14 to 0.56)		
				Total femoral cartilage	Total femoral cartilage		
				*β* = 0.07	*β* = 0.13		
				(0.003 to 0.14)	(0.02 to 0.25)		
Ding 2006 [18]	Baseline MRI tibial bone area (C) TFJ	Change in semi-quantitative MRI cartilage defect scores over 2.3 years (L) TFJ	Age, sex, BMI, radiographic OA \features	NA	OR (95%CI)	-	High (61)
					Medial TFJ		
					1.24 (1.01 to 1.51)		
					*p* = 0.04		
					Lateral TFJ		
					2.07 (1.52 to 2.82)		
					*p* <0.001		
Everhart 2014 [114]	Baseline TFJ subchondral surface ratio of medial and lateral TFJ compartments (C)	Radiographic progression of lateral or medial TFJ knee OA at 48 months (L)	Sex, race, age, BMI, tobacco use, activity level, knee coronal alignment, baseline symptoms, injury history, surgery history, KL grade, and JSW	Unadjusted medial SSR vs progression of medial JSN	Neither medial nor lateral SSR was associated lateral or medial ROA progression in adjusted analysis *p* <0.05.	NA	High (61)
				OR 1.43, 95 % CI 1.15 to 1.77			
				*p* = 0.0015			
				Medial SSR vs progression of lateral JSN			
				OR 1.87, 95 % CI 1.44 to 2.42			
				*p* <0.001			
Davies-Tuck 2008 [112]	Baseline MRI tibial bone plateau area (C) TFJ	Progressive semi-quantitative cartilage defect score (L) medial and lateral TFJ	Age, sex, BMI, baseline cartilage defect score, baseline cartilage volume and baseline tibial plateau area	Lateral TFJ	OR (95 % CI)	+	High (56)
				OR (95 % CI) −0.01 (−0.06 to 0.03) *p* = 0.59	Lateral TFJ 0.06 (0.004 to 0.11) *p* = 0.03 Medial TFJ 0.07 (0.03 to 0.12) *p* = 0.002		
Carnes 2012 [113]	MRI tibial bone area (C)	Semi-quantitative cartilage defect progression TFJ (L)	Age, sex, BMI, cartilage defects, BML	Lateral tibial bone area OR 1.11, 95 % CI 1.0 to 1.23	OR (95 % CI) bone area medial 1.12 (1.01 to 1.26) and lateral tibial (1.35 (1.12 to 1.63)	+	Low (50)
Dore 2010 [68]	Baseline tibial bone area MRI (C)	Increase or no increase in semi-quantitative MRI tibial cartilage defects over 2.7 years (L)	Age, sex, body mass index, baseline cartilage defects, and subchondral bone mineral density	NR	OR (95 % CI) medial tibia 1.6 (1.0 to 2.6) *p* = 0.04 lateral tibia 2.4 (1.4 to 4.0) *p* <0.01	+ Bone area size is associated with increasing cartilage defect scores	Low (50)
Hudelmaier 2013 [180] Abstract	Annual change in segmented MRI knee bone area (L)	Baseline KL grade (C)	Nil	Medial tibia *p* <0.05	NR	+ The higher the KL grade the larger the increase in bone area	Low (50)
MRI bone shape/dimension - cross-sectional studies							
Ding 2005 [19]	MRI quantitative tibial bone area (C)	Semi-quantitative MRI knee cartilage defect severity scores (C) TFJ	Age, sex, BMI, family history, cartilage volume	*β* (95 % CI) medial TFJ 0.06 (0.03 to 0.09) lateral TFJ 0.09 (0.05 to 0.13)	*β* (95 % CI) medial TFJ 0.11 (0.07 to 0.15) lateral TFJ 0.17 (0.11 to 0.22)	+ Association maintained for the whole TFJ and by compartment	High (64)
Kalichman 2007 [165]	MRI patellar length ratio, trochlea sulcus angle (C)	JSN grade (C)	Age, sex, BMI	NR	Trochlea sulcus angle *p* for trend, medial JSN *p* = 0.0162, lateral JSN *p* = 0.1206	NC	High (64)
Kalichman 2007 [115]	MRI patellar length ratio, trochlea sulcus angle (C)	Cartilage defect (WORMS) (C)	Age, sex, BMI	NR	Trochlea sulcus angle *p* for trend, medial cartilage loss *p* = 0.0016, lateral cartilage loss *p* = 0.0009	+	Low (57)
Stefanik 2012 [116]	MRI lateral trochlear inclination and trochlear angle (C)	Semi-quantitative cartilage defect (WORMS) (C)	Age, sex, BMI	NR	Lateral trochlear inclination OR 2, 95 % CI 1.9 to 3.7, *p* <0.0001, trochlear angle OR 2.0, 95 % CI 1.2 to 3.5, *p* <0.0001	+	Low (57)
Frobell 2010 [107]	MRI bone area - manual segmentation (C)	KL grade, OARSI JSN grade (C)	Age and BMI	Medial tibia JSN and KL p <0.0125	Medial tibia JSN and KL *p* <0.0125	+	Low (57)
Wang 2005 [66]	Annual % change in tibial bone area (L) 2 years follow up	Baseline JSN (C)	Age, sex, BMI, WOMAC score, SF-36 score, physical activity, radiographic OA features, baseline tibial plateau bone area.	*β* (95 % CI) medial tibia *β* = 0.35 (−1.10 to 1.80) *p* = 0.63, lateral tibia −0.87 (−2.35 to 0.61) *p* = 0.25	*β* (95 % CI) medial tibia 1.88 (0.43 to 3.33) *p* = 0.01 lateral tibia −0.42 (−2.31 to 1.48) *p* = 0.66	+ Association with medial tibia but not in the lateral tibia	Low (57)
Jones 2004 [23]	Tibial bone area (MRI) (C)	Radiographic JSN (C)	Age, sex, height, weight	*β* (95 % CI) medial tibia *β* = −0.03 (−0.11 to 0.06), lateral tibia −0.00 (−0.07 to 0.06)	β (95 % CI) medial tibia *β* = −0.00 (−0.04 to 0.06), lateral tibia +0.00 (−0.04 to 0.05)	NA	Low (50)
Eckstein 2010 [117]	MRI tibial bone area (segmented) (C)	OARSI JSN grade (C)	Nil	*p* <0.01	NR	+	Low (43)
MRI bone shape/dimension - case–control studies							
Bowes 2013 [118]	Change in segmented MRI 3D bone area over 4 years (L)	KL grade defined ROA knee (C) and (L)	Nil	NR bone area increased significantly faster in ROA vs non-ROA *p* <0.0001	NR	+ Higher KL grades had greater increase in bone area,	High (71)
Neogi 2013 [120]	MRI 3D bone shape (tibia, femur and patella) (C)	Incident TFJ ROA KL grade ≥2 (L)	Age, sex, BMI	NR	OR 3, 95 % CI 1.8 to 5.0	+ Developing 3D OA knee shape is associated with increasing ROA knee	High (65)
Hunter 2013 abstract [119]	Change in MRI knee bone area over 24 months (L)	Incident TFJ ROA (KL grade ≥2) (L)	NR	NR	Hazard ratio (95 % CI) range from 1.17 (1.08 to 1.27) to 3.97 (2.38 to 6.63), all highly statistically significant	+ for all bone regions Enlarging bone area associated with increasing ROA knee	Low (59)
Wluka 2005 [121]	Change in MRI tibial bone area (L)	Baseline radiographic JSN (C)	Age, BMI, pain, physical activity	Medial tibial bone area *R* = 160, 95 % CI 120 to 201, *p* <0.001	Medial tibial bone area *R* = 145, 95 % CI 103 to 186, *p* <0.001	+	Low (47)
MRI bone cyst - cohort studies							
Kotharii 2010 [82]	Semi-quantitative baseline MRI bone cyst (WORMS) (C) TFJ	Semi-quantitative cartilage defect score change over 2 years (WORMS) (L) TFJ.	Age, sex BMI, other bone lesions	OR 1.66, 95 % CI 0.55 to 4.99	OR 0.47, 95 % CI 0.11 to 2.03	NA	Low (50)
Tanamas 2010 [88]	Semi-quantitative change in MRI bone cyst size (L)	Knee Cartilage volume loss over 2 years (L) TFJ	Nil	*β* (95 % CI) lateral tibial cartilage loss in cyst regression relative to stable and progressive cysts	NR	+	Low (47)
				*β* = −11.81 (−16.64 to −6.98)			
Madan-Sharma 2008 [93]	Baseline MRI semi-quantitative bone cyst (C) TFJ	OARSI medial TFJ JSN grade progression over 2 years (L) TFJ	Age, sex, BMI and family effect	NR	*RR* 1.6, 95 % CI 0.5 to 4.0	NA	Low (47)
Carrino 2006 [77]	Crude presence of MRI bone cyst TFJ (C) and (L)	Any grade of cartilage defect TFJ (C) and (L)	Nil	NR	NR	+	Low (22)
MRI bone cyst -– cross-sectional studies							
Stehling 2010 [65]	Presence of any MRI semi-quantitative cyst (C)	Presence of any WORMS MRI cartilage defects (C)	Age, gender and BMI, KL score, knee injury or knee surgery, family history of TKR and Heberden’s nodes	NR	*p* = 0.0131	+	High (71)
Torres 2006 [103]	MRI bone cyst (WORMS) TFJ and PFJ (C)	Semi-quantitative cartilage (WORMS) TFJ and PFJ (C)	Nil	*R* = 0.75		NC	High (68)
Hayes 2005 [22]	Semi-quantitative MRI bone cyst (C)	KL grade (C)	Nil	*p* = 0.02	NR	+	High (61)
Link 2003 [101]	Crude presence of MRI bone cyst (C)	KL grade (C)	Nil	*p* <0.01	NR	+	Low (54)
Crema 2010 [122]	MRI Bone cysts (WORMS) (C)	Cartilage defect (WORMS) (C)	Nil	NR	NR	+	Low (50)
CT bone cyst – cross-sectional studies							
Okazaki 2014 [40]	Number of CT bone cysts (medial femur and tibia) (C)	Knee KL grade (C)	Nil	p <0.05	Nil	+with KL grade in medial TFJ	Low (50)
MRI subchondral bone morphometry - cohort studies							
Lo 2012 Abstract [53]	MRI BVF, trabecular number, thickness and spacing (C)	OARSI medial TFJ JSN progression between 24 and 48 months (L)	Nil	OR 2.4, 95 % CI 1.1 to 5.0, *p* = 0.02	NR	BVF, trabecular number and thickness are positively associated with JSN progression but negatively associated with trabecular spacing.	Low (50)
MRI subchondral bone morphometry - cross-sectional studies							
Driban 2011 [50] Abstract	MRI bone volume fraction, trabecular number, spacing & thickness of medial tibia (C)	The presence of any grade of radiographic medial & lateral JSN (C)	Nil	*R* = 0.09 to 1.77	NR	+ Medial JSN associated with higher BVF, trabecular number and thickness but lower spacing	High (71)
Driban 2011 [49]	MRI bone volume fraction (C)	Radiographic JSN (C)	Nil	NR	NR	+ Higher JSN score, lower JSW) were associated with higher BVF	High (64)
Lindsey 2004 [123]	MRI bone volume fraction trabecular and trabecular number (TFJ) (C)	Cartilage volume of tibia or femur in contralateral TFJ compartment (C)	Nil	Medial TFJ cartilage with lateral TFJ BVF and trabecular number. *β* = 0.29 to 0.36, *p* = 0.0020 to 0.02	NR	+ With contralateral BVF and trabecular number, but – with trabecular spacing	High (64)
Lo 2012 [54]	MRI bone volume fraction, trabecular thickness, number, spacing and DXA BMD of (proximal medial tibia) (C)	Radiographic medial JSN grade (C)	Nil	All *p* <0.0001	Nil	+ (BV/TV, thickness, number, BMD) (spacing)	High (64)
Chiba 2012 [34]	MRI bone volume fraction and trabecular thickness of the medial & lateral femur & tibia. (C)	Metric JSW (radiographic) of the medial and lateral TFJ (C)	Nil	Bone volume fraction −0.48 (*p* <0.001) trabecular thickness −0.51 (*p* <0.001)	NR	-	Low (57)
DXA BMD - cohort studies							
Dore 2010 [68]	Baseline proximal tibial BMD, DXA (C)	Increase or no increase in semi-quantitative MRI tibial cartilage defects over 2.7 years (L)	Age, sex, BMI, baseline cartilage defects and subchondral tibial bone area	NR	OR (95 % CI) medial tibia 1.6 (1.2 to 2.1) *p* <0.01 lateral tibia 1.2 (0.9, 1.6) *p* = 0.19	+ Association only observed in medial tibia	Low (50)
Lo 2012 Abstract [53]	DXA-measured medial:lateral periarticular BMD (paBMD) (C)	OARSI medial TFJ JSN progression (L)	Nil	OR 8.4, 95 % CI 2.8 to 25.0, *p* <0.0001	nil	+ JSN association with baseline M:L paBMD	Low (50)
Bruyere 2003 [42]	Subchondral tibial bone BMD (DXA) (C)	Minimum medial JSW TFJ after one year (L)	Age, sex, BMI, minimum JSW	NR	*R* = −0.43, *p* = 0.02	Negative correlation i.e., lower BMD gives bigger JSW or less JSN	Low (44)
DXA BMD - cross-sectional studies							
Dore 2009 [52]	DXA tibial subchondral BMD (C)	Radiograph JSN grade and MRI cartilage defect and volume (C)	Age, sex BMI	NR	Medial tibial BMD vs JSN *R* = 0.11, *p* <0.01, defect *R* = 0.16, *p* <0.01, cartilage volume *R* = 0.12, *p* = 0.01	+ Higher the BMD the greater the JSN and cartilage defects,	High (71)
Lo 2006 [55]	DXA medial:lateral BMD ratio at the tibial plateau (C)	Radiographic JSN grade (medial and lateral TFJ) (C)	Age, sex, BMI	*p* <0.0001	NR	+ With medial JSN, − with lateral JSN	High (71)
Lo 2012 [54]	DXA BMD (proximal medial tibia) (C)	Radiographic medial JSN grade (C)	Nil	*p* <0.0001	NR	+	High (64)
Akamatsu 2014 [31] Abstract	BMD (DXA) (C) (medial tibia and femoral condyle)	Medial TFJ JSN (radiographic) (C)	Nil	Tibia *R* = 0.571, *p* <0.001 femur *R* = 0.550, *p* < 0.001	NR	+ Medial femoral and tibial condyle BMD correlated with medial JSN	Low (57)
Volumetric CT BMD - case control studies							
Bennell 2008 [56]	Volumetric BMD in tibial subchondral trabecular bone (C)	KL grade (C)	Age, sex, BMI	NR	*p* <0.05	NC BMD falls in posterior tibial plateau as KL increases but anteriorly increase in BMD noted	Low (59)
Knee scintigraphic subchondral bone cohort studies							
Mazzuca 2004 [37]	Baseline late-phase subchondral bone scintigraphy (adjusted for healthy diaphysis uptake) of the medial tibia and whole knee (C)	Progression of minimum JSN of the medial TFJ from baseline to 30 months (L)	Age, BMI, KL grade (NB all women)	*r* = 0.22 to 0.30 (*p* <0.05)	*r* = 0 to 0.08 (*p* <0.05)	NA after adjustment for covariates	High (56)
Mazzuca 2005 [38]	Baseline late-phase subchondral bone scintigraphy (adjusted for healthy diaphysis uptake) of the medial tibia and whole knee (C)	Progression of minimum JSN of the medial TFJ from baseline to 30 months (L)	Baseline JSW, treatment group	NR	Coefficient 0.221, 95 % CI 0.003 to 0.439, *p* = 0.049	+ The greater the scintigraphic bone signal the greater the JSN	High (56)
Dieppe 1993 [58]	Baseline late and or early-phase subchondral bone scintigraphy signal (C)	Progression of JSN by ≥2 mm or knee operation incidence after 5 years (L)	Nil	*p* <0.005	NR	+	Low (50)
Knee scintigraphic subchondral bone cross-sectional studies							
Kraus 2009 [59]	Ipsilateral late-phase bone scintigraphy, semi-quantitative retention scoring of TFJ (C)	Ipsilateral OARSI scale of JSN (C)	Age, gender, BMI, osteophyte OARSI score, knee alignment knee symptoms	Coefficient 0.47 to 0.48 (*p* <0.0001)	Coefficient 0.26 to 0.29 (*p* = 0.0005 to 0.001)	+	High (71)
McCrae 1992 [62]	Late-phase ‘extended bone uptake’ pattern bone scintigraphy, presence around the TFJ (C)	Radiographic JSN presence (C)	Nil	OR 47.3, 95% CI 6.4 to 352, *p* <0.01	NR	+	Low (50)
2D knee bone shape – cross-sectional studies							
Haverkamp 2011 [36]	2D bone shape knee. 1. Femur and tibial width 2. Elevation of lateral tibial plateau (C)	1. Presence of diffuse cartilage defects semi-quantitative scoring (MRI). 2. Presence of ROA knee (KL ≥2) (C)	NB (this is a population of women only) ROA models adjusted for age, BMI; cartilage defect models adjusted for KL only	OR (95 % CI) bone width vs knee ROA 2.03 (1.55 to 2.66) *p* <0.001 bone width Presence of diffuse cartilage defects *p* <0.001	OR (95 % CI) knee ROA 1.94 (1.44 to 2.62) *p* <0.001	+ Wider bones and elevated tibial plateau were associated with the presence of ROA knee. Cartilage defects were only associated with bone width	Low (46)

Positive correlation reported between bone feature and outcome measure (+); negative correlation reported between bone feature and outcome measure (−). *BMD* bone mineral density, *BMI* body mass index, *BML* bone marrow lesion, *BOKS* Boston osteoarthritis of the knee study, *BLOKS* Boston–Leeds osteoarthritis knee score, *BVF* bone volume fraction, *C* a feature or outcome described in cross-section, *CT* computed tomography, *DXA* dual-energy x-ray absorptiometry, *GARP* Genetics, osteoarthritis and progression study, *JSN* joint space narrowing, *JSW* joint space width, *KL* Kellgren-Lawrence, *KOSS* knee osteoarthritis scoring system, *L* a feature or outcome described longitudinally, *MAK-*2 mechanical factors in arthritis of the knee 2. *NC* no conclusion could be found for an association between bone feature and outcome measure, *SWAN* Michigan study of women’s health across the nation, *MOST* multicentre osteoarthritis study, *MRI* magnetic resonance imaging, *NA* no association. *NR* not reported, *OA* osteoarthritis, *OAI* Osteoarthritis Initiative, *OR* odds ratio, *RR* relative risk ratio, *SSR* subchondral surface ratio *TASOAC* Tasmanian older adult cohort, *TFJ* tibiofemoral joint, *VAS* visual analogue scale, *WOMAC* Western Ontario and McMaster Universities arthritis index, *WORMS* whole-organ magnetic resonance imaging score, *CRP* C-reactive protein, *TKR* total knee replacement, *OARSI* Osteoarthritis Research Society International, *PFJ* patellofemoral joint, *ROA* radiographic osteoarthritis

#### Bone marrow lesions

MRI (31 cohort, 15 cross-sectional, 4 case–control studies): in prospective cohorts with high- quality, well-adjusted analyses the presence and increasing size of baseline BMLs and incidence of BMLs conferred greater odds of structural progression [67, 70–73]. Similarly increasing baseline BML size increased the risk of total knee replacement (TKR) and expedited the outcome of TKR [44, 74–76]. The association between BMLs and structural progression of OA was maintained in cohorts without clinical features of knee OA [67] and in analyses with poorer quality or statistical adjustment [25, 28, 76–90]. Only five low quality cohort analyses did not support these findings [91–95]. All cross-sectional analyses found positive correlation between BMLs and structural severity of OA [22, 29, 35, 63–65, 87, 96–103]. Three case–control analyses found similar associations [17, 104, 105]. In summary, BMLs are independently associated with structural progression of OA of the knee and incident TKR.

#### Osteophytes

MRI (three cohorts, eight cross-sectional studies): in one prospective cohort with high quality and well-adjusted analysis, the increasing size of osteophytes conferred greater odds of structural progression of OA [106]. In lower quality, inadequately adjusted, prospective cohorts, increasing osteophyte size increased the risk of incident TKR and structural progression of OA [28, 45]. The increasing size and presence of osteophytes was associated with greater structural progression or severity in all included analyses [22, 26, 28, 45, 64, 65, 97, 101, 103, 106–108]. In summary, osteophytes are independently associated with knee structural progression and are associated with TKR incidence.

#### Bone attrition

MRI (one cohort, two cross-sectional, one case–control study): one prospective, well-adjusted, but below-average-quality cohort analysis found an association with baseline attrition severity and structural progression that became insignificant after covariate adjustment [82]. The unadjusted cross-sectional analyses and case–control analysis found similar associations with structural severity [103, 109, 110]. In summary, bone attrition is associated, but not independently so, with structural progression.

#### Bone shape/dimension

MRI (eight cohort, seven cross-sectional, four case–control studies): in prospective cohorts with high quality well-adjusted analyses, greater baseline tibial plateau bone area conferred greater odds of structural progression of OA and incidence of TKR [18, 20, 111, 112]. The same association was observed in a lower quality, prospective-cohort, well-adjusted analysis [113] and in a study of the knee in patients who predominantly had no radiographic evidence of knee OA [18]. The mismatch ratio of the femoral and tibial articulating areas was not associated with structural progression after adjustment [114], but the trochlear sulcus angle and shape was associated with cross-sectional patellofemoral structural severity demonstrated on MRI [115, 116]. All cross-sectional [23, 66, 107, 117] and case–control [118–121] analyses of tibial bone area or 3D knee bone shape found association with structural severity [23, 66, 107, 117–121]. In summary, tibial bone area is independently associated with structural progression of OA of the knee and incidence of TKR.

#### Bone cyst

MRI and CT (five cohort, five cross-sectional) studies: two prospective cohorts with well-adjusted but below-average-quality analyses of cysts reported no association with structural progression of OA before or after adjustment [82, 93]. Two prospective cohorts with low quality unadjusted analyses of cysts found an association with structural progression of OA [77, 88]. Cross-sectional well-adjusted [65] and unadjusted [22, 40, 101, 122] cyst analyses found an association with structural severity. In summary, after covariate adjustment there is no independent association between cysts and structural progression of OA.

#### Trabecular bone morphometry

MRI (one cohort, five cross-sectional studies): one prospective cohort, unadjusted, below-average-quality analysis reported increasing bone volume fraction, trabecular number and thickness and decreasing trabecular spacing were associated with structural progression [53]. The same bone changes were associated with structural severity in cross-sectional unadjusted analyses [34, 49, 50, 54, 123]. In summary, increasing bone volume fraction, trabecular number, trabecular thickness, and decreasing trabecular spacing are associated with structural progression and severity of OA of the knee.

#### Peri-articular bone mineral density

DXA and CT (three cohort, four cross-sectional, one–case control study): two prospective cohorts with well-adjusted but below-average-quality analyses reported that increasing tibial subchondral BMD is associated with structural progression of OA [42, 68]. In one prospective cohort with an unadjusted below-average-quality analysis, the medial-to-lateral ratio of tibial peri-articular BMD was associated with structural progression [53]. All cross-sectional analyses [31, 52, 54, 55], including two that were well-adjusted [52, 55], reported increasing BMD with greater structural severity. One well-adjusted analysis using quantitative CT (qCT) reported higher and lower BMD in the anterior and posterior tibial plateau, respectively, in knees of patients with moderate OA relative to asymptomatic controls. In summary, increasing peri-articular radiographic BMD is associated with structural progression and severity of OA.

#### Scintigraphy

Scintigraphy (three cohort, two cross-sectional studies): prospective cohorts with high quality analyses found greater late-phase bone signal was associated with structural progression of OA, with no or inadequate covariate adjustment [37, 38], but not after adequate covariate adjustment [37]. A prospective cohort, with below-average-quality, unadjusted analysis found greater bone signal was associated with structural progression of OA [58]. Bone signal was associated with structural severity in well-adjusted and unadjusted cross-sectional analyses [59, 62]. In summary, bone scintigraphy signal is associated, but not independently so, with structural progression of OA.

#### 2D Knee bone shape

One cross-sectional, well-adjusted analysis identified an association between greater femoral and tibial bone width and elevating tibial plateau, and greater structural severity [36]. In summary, 2D bone shape is associated with structural severity of OA.

### Relationship between knee bone feature and pain

The association between bone features and pain is described in Tables 2 and 5. In all types of study, bone features were compared with the presence, chronicity and severity of pain. In longitudinal studies, bone features were also compared with change in the presence or severity of pain (e.g., change in Western Ontario and McMaster Universities arthritis index (WOMAC) pain score). Change in the presence of pain included developing new frequent pain, [49], or the resolution of existing pain.

**Table 2:** Knee pain associations by feature and quality score

Author	Feature (method)	Knee pain outcome	Adjustment for confounders	Association (magnitude) crude	Association (magnitude) adjusted	Association	Quality score (%)
MRI bone marrow lesion - cohort studies							
Foong 2014 [21]	Change in BML size (L) and incident BMLs (L) in all three knee compartments	WOMAC Knee pain severity at 2-year and 10-year visits (L)	Age, sex, BMI, leg strength, and the presence of ROA	NR	Incident or change in total BML size *β* = 1.53 (95 % CI 0.37 to 2.70.	+	High (67)
					Medial tibial change in BML size *β* = 2.96 (95 % CI 0.59-5.34	Incidence of BML or increase in size associated with increase in pain in the medial tibia	
Driban 2013 [72]	Knee baseline BML volume (C), BML volume change (L) (TFJ)	48-month change in WOMAC pain (L)	Age, sex, BMI	NR	*β* = 0.21	+	High (61)
					(standard error 0.07)	Longitudinal (L) changes in BML correlated with (L) changes in pain severity	
					*p* = 0.004		
Dore 2010 [124]	MRI BML size (L) regional or whole TFJ over 2.7 years	Change in WOMAC pain (L) over 2.7 years	Age, sex, BMI, leg strength, quality of life, and baseline pain, function	*β* (95 % CI) Total BML size change = 1.06 (0.10 to 2.03)	*β* (95 % CI) total BML size change = 1.13 (0.28 to 1.98)	+	High (56)
Kornaat 2007 [173]	Semi-quantitative MRI BML change over 2 years (L) TFJ	Mean WOMAC pain over 2 years	Age, sex and BMI	NR	*β* (95 % CI) = 2 (−8 to 11)	NA	High (56)
Moisio 2009 [125]	Baseline MRI semi-quantitative BML score (C) TFJ and PFJ	Incident frequent knee pain 2 years after baseline (L)	Age, sex, BMI, BML score, % denuded bone	NR	OR (95 % CI) medial tibia and femur 1.41 (0.86 to 2.33), lateral tibia and femur 1.70 (1.07 to 2.69)	+ Lateral TFJ BML score associated with incident frequent knee pain	High (56)
Sowers 2011 [28]	Semi-quantitative MRI BML, size in TFJ (C)	Increasing WOMAC pain (L)	Nil	Medial and lateral TFJ BMLs both *p* <0.005	NR	+	Low (53)
Zhang 2011 [126]	Semi-quantitative change in MRI BML size (L) TFJ over 30 months	Incidence of frequent knee pain, and categorical severity (L) over 30 months	Synovitis and effusions	OR (95 % CI) Severity of frequent knee pain OR 3.0 (1.5 to 6.0)	OR (95 % CI) Incident frequent knee pain *p* for trend = 0.006. Severity of frequent knee pain OR 2.2 (1.0 to 4.7) *p* = 0.047	+ Ipsilateral association	Low (50)
Wildi 2010 [95]	24-month change in regional TFJ BML score WORMS (L)	24-month change in WOMAC pain (L)	Nil	*R* <0.15, *p* >0.067 for all compartments	NR	NA, all compartments had no correlation	Low (50)
Tanamas 2010 [89]	Baseline semi-quantitative MRI BML size (C)	Annual change in WOMAC pain (L)	Nil	NR	NR	NA	Low (50)
MRI bone marrow lesion - cross-sectional studies							
Zhai 2006 [135]	Semi-quantitative MRI BML (C)	WOMAC pain >1 (C)	Age, BMI, sex, knee strength, chondral defects	NR	OR 1.44, 95 % CI 1.04, 2.00	+	High (79)
Sharma 2014 [133]	Semi-quantitative BML score WORMS TFJ or PFJ (C)	Prevalent frequent knee symptoms (C)	Age, sex, body mass index (BMI), previous knee injury, and previous knee surgery	NR	BMLs in any compartment OR 1.96, 95 % CI 1.38 to 2.77	+ BML association with prevalent knee symptoms	High (71)
Kornaat 2006 [130]	Semi-quantitative MRI BML (C)	Chronic pain presence (C)	Age, sex, and BMI	NR	OR 1.13, 95 % 0.41, 3.11, *p* = 0.76	NA	High (71)
Lo 2009 [131]	Semi-quantitative MRI BML (BLOKS) (C)	WOMAC pain (C)	Synovitis, effusion scores	*p* for trend = 0.0009	*p* for trend = 0.006	+	High (71)
Stefanik 2014 abstract [134]	BML (WORMS) (C) (patellofemoral joint)	Prevalent knee pain (any pain in last 30 days) and pain VAS (C)	Adjusted for age, sex, BMI, depressive symptoms and TFJ BMLs	NR	Isolated BML of the lateral PFJ, OR (95 % CI) 1.4 (0.9 to 2.0); medial PFJ, OR (95 % CI) 1.1 (0.8 to 1.5). Isolated lateral PFJ BMLs OR 6.6 (1.7 to 11.5)	NC	High (71)
Ratzlaff 2013 [132]	Total BML volume in the femur or tibia (C)	Weight-bearing knee pain WOMAC subscale (C)	Age, sex, BMI, race, and medial minimum joint space width	NR	Total BML volume femur *p* = 0.003, tibia *p* = 0.101	+ Femoral NA Tibial	High (71)
Ip 2011 [99]	Semi-quantitative MRI BML (C)	WOMAC pain (C)	Age, sex, BMI, OA stage, joint effusion, and meniscal damage	NR	Total WOMAC pain	NC	High (68)
					*R* = 0.05, 95 % CI −0.04 to 0.14. Stair climbing pain *R* = 0.09 (0.00 to 0.18)		
Torres 2006 [103]	MRI BML (WORMS) TFJ and PFJ (C)	Pain VAS (C)	Age, BMI	Coefficient 5.00, 95 % CI 3.00 to 7.00	Coefficient 3.72, 95 % CI 1.76 to 5.68	+	High (68)
Kim 2013 [129]	Summary score and severity of MRI BML (WORMS) (C)	WOMAC pain severity or presence of knee pain (C)	Age, sex, BMI, radiographic OA	NR	BML summary score medial TFJ OR 2.33, 95 % CI 1.02 to 5.33, *p* <0.001	+ Severity of BML is proportional to WOMAC in medial compartment after adjustment	High (64)
Moisio 2009 [125]	Baseline MRI semi-quantitative BML score (C) TFJ and PFJ	Presence of baseline moderate to severe knee pain (C)	Percent denuded bone, age, sex, BMI	NR	Bone marrow lesion score, OR 0.95, 95 % CI 0.63 to 1.44. Not significant in all compartments	NA found on cross-sectional analysis	High (64)
Ratzlaff 2014 [48] Abstract	Median BML volume (PFJ, TFJ) (C)	Stair-climbing knee pain WOMAC (C)	Nil	TFJ *p* = 0.01, patellofemoral *p* = 0.01, femur *p* = 0.02, tibia *p* = 0.03	NR	+	High (64)
Hayes 2005 [22]	Semi-quantitative MRI BML (C)	Chronic pain presence (C)	Nil	*p* = 0.001	NR	+	High (61)
Ai 2010 [127]	Semi-quantitative MRI BML (C)	Pain verbal rating scale (Likert) (C)	Nil	*p* = 0.33	NR	NA	Low (57)
Bilgici 2010 [128]	MRI BML (WORMS) (C)	WOMAC pain, pain VAS (C)	Nil	WOMAC *r* = 0.508, *p* <0.01 Pain VAS *r* = 0.488, *p* <0.01	NR	+	Low (57)
Sowers 2003 [29]	Semi-quantitative MRI BML (C)	Chronic pain presence (C)	Nil	OR 5.0, 95 % CI 2.4 to 10.5	NR	+	Low (54)
Link 2003 [101]	Semi-quantitative MRI BML (C)	WOMAC pain (C)	Nil	*p* >0.05	NR	NA	Low (54)
Felson 2001 [96]	Semi-quantitative MRI BMLs (C)	Chronic knee pain presence (C)	Radiographic severity, age, sex, and effusion score	*p* <0.001	OR 3.31, 95 % CI 1.54 to 7.41	+	Low (54)
Fernandez-Madrid 1994 [97]	Crude presence of MRI BMLs (C)	Crude pain presence (C)	Nil	NR	NR	NA	Low (46)
MRI bone marrow lesion - case−control studies							
Javaid 2010 [140]	Baseline semi-quantitative MRI BML size (WORMS) (C) TFJ and PFJ	Incident frequent knee pain after 15 months (L)	Age, sex, race, BMI	NR	Whole knee OR 2.8, 95 % CI 1.2 to 6.5	+	High (76)
Felson 2007 [181]	Semi-quantitative MRI BML size increase (WORMS) (L) TFJ and PFJ	Incident frequent pain at 15 months (L)	Age, sex, race, BMI, quadriceps strength, KL score, malalignment, baseline BML score	OR 4.1, 95 % CI 2.1 to 8.1	OR 3.2, 95 % CI 1.5 to 6.8	+	High (71)
Javaid 2012 [139]	Baseline Semi-quantitative MRI BML, (WORMS) (C) TFJ and PFJ	Presence of frequent knee pain (C) after 2 years	Nil	OR 1.70, 95 % CI 1.08 to	NR	+	Low (59)
Zhao 2010 [105]	Baseline crude presence of MRI BMLs at (C) TFJ	Change in WOMAC Pain (L)	Nil	*p* = 0.60	NR	NA	Low (56)
Stahl 2011 [41]	Semi-quantitative MRI BML size (WORMS) (L) TFJ	Changes in WOMAC score (L)	Nil	NR	Data not shown	NA	Low (47)
MRI osteophyte – cohort studies							
Sowers 2011 [28]	Semi-quantitative MRI osteophyte, size in TFJ (C)	Increasing WOMAC pain (L)	Nil	Medial and lateral TFJ BMLs both *p* <0.001	NR	+	Low (53)
MRI osteophyte - cross-sectional studies							
Kornaat 2006 [130]	Semi-quantitative MRI osteophyte (C)	Chronic pain presence (C)	Age, sex, BMI	NR	Patellofemoral OR 2.25, 95 % CI 1.06 to 4.77	+	High (71)
Sengupta 2006 [136]	Semi-quantitative MRI osteophyte (WORMS) (C)	Pain severity WOMAC, chronic pain (C)	Age, sex, BMI	NR	OR 0.97, 95 % CI 0.86 to 1.10	NA	High (71)
Torres 2006 [103]	MRI osteophyte, (WORMS) TFJ and PFJ (C)	Pain VAS (C)	Nil	Coefficient 1.18, 95 % CI 0.63 to 1.72	Coefficient 0.50, 95 % CI 0.07 to 0.94	NC	High (68)
Hayes 2005 [22]	Semi-quantitative MRI osteophyte (C)	Chronic pain presence (C)	Nil	*p* <0.001	NR	+	High (61)
Ai 2010 [127]	Semi-quantitative MRI osteophytes (C)	Pain verbal rating scale (Likert) (C)	Nil	*p* = 0.166	NR	NA	Low (57)
Hayashi 2012 [137]	Crude presence of MRI osteophytes (C)	Presence of pain on WOMAC pain subscale (C)	Nil	OR 4.2 to 6.4, *p* = 0.001-0.011	NR	+	Low (57)
Link 2003 [101]	Semi-quantitative MRI osteophytes (C)	WOMAC pain (C)	Nil	*p* >0.05	NR	NA	Low (54)
Fernandez-Madrid 1994 [97]	Crude presence of MRI osteophytes (C)	Crude pain presence (C)	Nil	NR	NR	NA	Low (46)
MRI osteophyte - case–control studies							
Javaid 2010 [140]	Baseline semi-quantitative MRI osteophyte, size (WORMS) (C) TFJ and PFJ	Incident frequent knee pain after 15 months (L)	Age, sex, race, BMI	NR	Whole knee severe osteophyte OR 4.7, 95 % CI 1.3 to 18	+	High (76)
MRI bone attrition - cross-sectional studies							
Hernandez-Molina 2008 [138]	Semi-quantitative MRI bone attrition (WORMS) (C)	Pain severity and nocturnal pain (WOMAC) (C)	Age, sex, BMI, BMLs, effusions and KL grade	OR (95 % CI) pain severity OR 1.6 (1.1 to 2.3), nocturnal pain OR 1.1 (0.5 to 2.1)	OR (95 % CI) pain severity OR 0.9 (0.6 to 1.4), nocturnal pain OR 1.0 (0.5 to 2.1).	NA	High (71)
Torres 2006 [103]	MRI attrition, (WORMS) TFJ and PFJ (C)	Pain VAS (C)	Nil	Coefficient 3.33, 95 % CI 1.79 to 4.87	Coefficient 1.91, 95 % CI 0.68 to 3.13	+	High (68)
MRI bone attrition - case−control studies							
Javaid 2012 [139]	Baseline semi-quantitative MRI attrition size (WORMS) (C) TFJ and PFJ	Presence of frequent knee pain (C) after 2 years	Nil	OR 2.40, 95 % CI 1.51 to 3.83	NR	+	Low (59)
MRI bone shape/dimension - cohort studies							
Everhart 2014 [114]	Baseline TFJ subchondral surface ratio of medial and lateral TFJ compartments (C)	Incident frequent knee pain at 48 months, (L)	Sex, race, age, BMI, tobacco use, activity level, knee coronal alignment, baseline symptoms, injury history, surgery history, KL grade, and JSW	NR	Medial SSR OR 0.48, 95 % CI 0.30 to 0.75, *p* = 0.0009. Lateral SSR OR 1.27, 95 % CI 0.86 to 1.88, *p* = 0.19	- larger MSSR gets less incident frequent knee pain	High (61)
MRI bone shape/dimension - cross-sectional studies							
Ochiai 2010 [47]	MRI irregularity of femoral condyle contour (C)	Knee pain VAS (C)	Nil	Irregularity of femoral condyle contour *r* = 0.472, *p* = 0.0021	NR	+	Low (50)
MRI bone cyst - cohort studies							
Sowers 2011 [28]	Semi-quantitative MRI bone cyst size in TFJ (C)	Increasing WOMAC pain (L)	Nil	NR	NR analysis described as not significant but data not shown	NA	Low (53)
MRI bone cyst - cross-sectional studies							
Kornaat 2006 [130]	Semi-quantitative MRI bone cyst(C)	Chronic pain presence (C)	Nil	NR	Patellofemoral OR 1.83, 95 % CI (0.80 to 4.16)	NA	High (71)
Torres 2006 [103]	MRI bone cyst (WORMS) TFJ and PFJ (C)	Pain VAS (C)	Age, BMI	Coefficient 2.50, 95 % CI −0.38 to 5.38	Coefficient 0.82, 95 % CI −0.50 to 2.14	NA	High (68)
Hayes 2005 [22]	Semi-quantitative MRI bone cyst (C)	Chronic pain presence (C)	Age, sex, and BMI	*p* <0.001	NR	+	High (61)
Hayashi 2012 [137]	Crude presence of MRI bone cysts (C)	Presence of pain on WOMAC pain subscale (C)	Nil	OR 6.7 to 17.8, p = 0.004 to 0.03	NR	+	Low (57)
Link 2003 [101]	Crude presence of MRI bone cyst (C)	WOMAC pain (C)	Nil	*p* >0.05	NR	NA	Low (54)
MRI bone cyst - case control studies							
Javaid 2010 [140]	Baseline semi-quantitative MRI bone cyst size (WORMS) (C) TFJ and PFJ	Incident frequent knee pain after 15 months (L)	Nil	NR	NR *p* >0.1	NA	High (76)
Javaid 2012 [139]	Baseline semi-quantitative MRI bone cyst size (WORMS) (C) TFJ and PFJ	Presence of frequent knee pain (C) after 2 years	Nil	OR 1.61, 95 % CI 1.03 to 2.52	NR	+	Low (59)
qCT bone mineral density - cross-sectional studies							
Burnett 2012 [57]	BMD of patellar lateral facet (qCT) (C)	WOMAC – knee pain at rest (C)	Nil	Total lateral patella facet *p* = 0.04, inferior lateral facet *p* = 0.005	NR		Low (57)
2D Knee bone shape - cross-sectional studies							
Haverkamp 2011 [36]	2D Bone shape knee, 1. femur and tibial width, 2. elevation of lateral tibial plateau (C)	Pain severity VAS (C)	Models adjusted for Age, BMI	NR	Bone width *p* = 0.167, lateral tibia plateau elevation *p* = 0.002	+ Lateral tibial plateau associated with pain severity, NA bone width with pain severity	Low (46)

Positive correlation reported between bone feature and outcome measure (+); negative correlation reported between bone feature and outcome measure (−). *BMI* body mass index, *BML* bone marrow lesion, *C* a feature or outcome described in cross-section, knee pain on most days for at least the last month (chronic pain) confidence interval (*CI*), *KL* Kellgren-Lawrence, *L* a feature or outcome described longitudinally, *NA* no association, *NC* no conclusion could be found for an association between bone feature and outcome measure, *NR* not reported, *OA* osteoarthritis, *OAI* Osteoarthritis Initiative, *OR* odds ratio, *PFJ* patellofemoral joint, *ROA* radiographic osteoarthritis, *SSR* subchondral surface ratio *VAS* visual analogue scale, *WOMAC* Western Ontario and McMaster Universities arthritis index, *qCT* quantitative computed tomography

#### Bone marrow lesions

MRI (9 cohort, 18 cross-sectional, 5 case–control studies): in 3 prospective cohort, well-adjusted, high quality analyses the baseline or longitudinal increase in size of BMLs was associated with longitudinally increasing knee WOMAC pain severity [21, 72, 124]. This association was observed in one [28] but not two [89, 95] similar prospective-cohort, unadjusted, lower quality analyses. Baseline BML size in the lateral but not the medial tibiofemoral joint was associated with incident frequent knee pain in a prospective-cohort, well-adjusted, high quality analysis [125]. Longitudinally increasing BML size was associated with incident frequent knee pain in a similar but inadequately adjusted analysis of below average quality [126]. In cross-sectional studies the size or presence of BMLs was inconsistently associated with the presence of a heterogenous range of pain measures, irrespective of adequate covariate adjustment [22, 29, 48, 96, 97, 99, 101, 103, 125, 127–135]. In summary, BMLs are independently associated with longitudinally increasing pain severity and are associated with incident frequent knee pain.

#### Osteophytes

MRI (one cohort, eight cross-sectional, one case–control study): one prospective cohort, unadjusted, below-average-quality analysis reported increasing baseline osteophyte size was associated with increasing WOMAC pain severity score [28]. In well-adjusted cross-sectional analyses, osteophyte size was associated with the presence [130] but not severity of pain [136]. In unadjusted cross-sectional analyses osteophytes were inconsistently associated with a heterogenous range of pain measures [22, 97, 101, 103, 127, 137]. In summary, osteophytes are associated with longitudinally increasing pain severity and the cross-sectional presence of pain.

#### Bone attrition

MRI (no cohort, two cross-sectional, one case–control study); cross-sectional analyses found greater attrition was associated with greater pain severity, without covariate adjustment [103, 138], but not after adequate covariate adjustment [138]. An unadjusted case–control analysis found an association between attrition and prevalent pain [139]. In summary, bone attrition is associated, but not independently so, with severity of pain.

#### Bone shape/dimension

MRI (one cohort, one cross-sectional study): one prospective, well-adjusted, high quality analysis found the femoro-tibial articulating surface mismatch was associated with incident frequent knee pain [114]. One unadjusted cross-sectional analysis found the irregularity of the femoral condyle surface was associated with severity of knee pain [47]. In summary, specific features of bone shape are independently associated with incident frequent knee pain and severity of pain.

#### Bone cyst

MRI (one cohort, five cross-sectional, two case–control studies): one prospective cohort, unadjusted, low quality analysis found no association between bone cyst size and increasing WOMAC pain score [28]. In mostly unadjusted cross-sectional [22, 101, 103, 130, 137] and case control analyses [139, 140] of heterogenous cyst measures and pain measures, an association between cysts and pain was inconsistently found. In summary, bone cysts may not be associated with longitudinal severity of pain and cross-sectional association with pain is uncertain.

#### 2D Knee bone shape

One inadequately adjusted cross-sectional analysis found an association between the elevation of the lateral tibial plateau and severity of pain [36]. In summary, 2D lateral tibial bone shape is associated with cross-sectional severity of pain.

### Relationship between hand bone feature and structural progression

The association between bone features and structural progression is described in Tables 3 and 5.

**Table 3:** Hand, hip and ankle structural associations by feature and quality grade

Author	Feature (method)	Structural severity or progression outcome	Adjustment for confounders	Association (magnitude) crude	Association (magnitude) adjusted	Association	Quality (score %)
Hand MRI bone marrow lesion case series							
Haugen 2014 [141]	BMLs - semi-quantitative at 2^nd^ to 5^th^ IPJs (C)	Progression of hand ROA (JSN, KL grade or new erosion) (L)	Age, sex, BMI,	OR 2.73, 95 % CI 1.29 to 5.78	NR	+	High (61)
						Bigger the BML, the more the JSN	
Hand MRI bone marrow lesion cross-sectional studies							
Haugen 2012 Abstract [143] 299	BML (Oslo MRI hand score) (C) IPJs	Radiographic JSN grade IPJ (OARSI atlas) (C)	Age, sex,	OR 10.0, 95 % CI 4.2 to 23	OR 4.4, 95 % CI 2.2 to 9.0	+	Low (43)
						BML score association with more JSN	
Haugen 2012 [142]	BML (Oslo MRI hand score) (C) IPJs	Hand KL grade of IPJs (C)	Age, sex	NR	OR (95 % CI)	+	High (64)
					BMLs 11 (5.5 to 21)		
					*p* <0.001		
Hand MRI osteophyte cross-sectional studies							
Haugen 2012 [142]	Osteophyte (Oslo MRI hand score) (C) IPJs	Hand KL grade of IPJs (C)	Age, sex	NR	OR (95 % CI)	+	High (64)
					osteophytes		
					415 (189 to 908)		
					*p* <0.001		
Hand MRI attrition cross-sectional studies							
Haugen 2012 [142]	Attrition (Oslo MRI hand score) (C) IPJs	Hand KL grade of IPJs (C)	Age, sex	NR	OR (95 % CI) attrition 87 (37 to 204)	+	High (64)
					*p* <0.001		
Hand MRI bone cyst cross-sectional studies							
Haugen 2012 [142]	Cyst (Oslo MRI hand score) (C) IPJs	Hand KL grade of IPJs (C)	Age, sex	NR	OR (95 % CI)	Nil	High (64)
					cysts 2.0 (0.6 to 6.3)		
					*p* = 0.26		
Hip MRI BML cross-sectional studies							
Neumann 2007 [46]	Semi-quantitative BMLs (C)	Semi-quantitative cartilage lesions (C)	Nil	*R* = 0.44, *p* ≤0.001	NR	+ Correlation between BML and cartilage lesions	Low (43)
Dawson 2013 Abstract [69]	Femoral head BMLs (MRI) (C)	1. Presence of hip OA. 2. Femoral head cartilage volume (MRI) (C)	Age, sex, BMI	NA	OA hip presence	+	Low (14)
						BMLs associated with diagnosis of hip OA	
					OR (95 % CI)		
					5.32 (1.78 to 15.9)		
					*p* = 0.003	BMLs inversely associated with cartilage volume	
					cartilage volume		
					regression coefficient (95 % CI)		
					−245.7 mm^3^		
					(−456 to −36) *p* = 0.02		
Hip CT bone morphometry cross-sectional studies							
Chiba 2011 [33]	Acetabular and femoral head subchondral trabecular morphometry: bone volume fraction, trabecular thickness, number, separation (CT) (C)	Hip joint space volume (CT) (C)	Nil	Femoral head Bone volume fraction *r* = −0.691, *p* <0.001	NR	Joint space narrowing is associated with increased bone volume fraction, trabecular thickening. trabecular number and spacing decrease	Low (57)
Hip DXA BMD cross-sectional studies							
Chaganti 2010 [30]	Femoral neck BMD (C) DXA	Hip ROA Modified Croft score (categorical 0–4) (C)	Age, BMI, height, activity level, race, 6-m walk pace, Nottingham muscle strength, inability to do chair stands, and clinic site,	NR	p <0.0001	+	High (64)
						Higher BMD for higher grade of OA of hip	
Antoniades 2000 [32]	DXA BMD of the femoral neck of left (nondominant) hip with ROA (C)	Radiographic OA (Croft score) (C)	BMI, lifetime physical activity, menopausal status, use of oestrogen, and smoking	OR 1.63, 95 % CI 1.06 to 2.50)	OR 1.80, 95 % CI 1.05 to 3.12	+ Association between BMD and hip ROA grade in the index hip	High (64)
						Higher OA grade means higher BMD	
2D Hip bone shape longitudinal studies							
Agricola 2013 [146]	Baseline 2D femoral and acetabular shape modes (segmented by statistical shape modelling) (C)	THR at or within 5 years (L)	Age, sex, BMI, shape modes	5 modes were associated with THR OR 1.71 to 2.01, *p* ≤0.001	3 modes were associated with THR OR 1.78 to 2.10, *p* ≤0.001	+ Increasing femoral head asphericity is associated with THR	High (72)
Agricola 2013 [147]	Baseline alpha angle (2D femur shape) dichotomous abnormal >60 °, normal ≤60 ° (C)	Incident ROA hip (KL >1), incident end-stage ROA hip (KL >2 or THR) at or within 5 years (L)	Age, sex, BMI, KL grade	OR (95 % CI) Incident ROA hip 6.82 (3.55 to 13.10) *p* <0.0001	OR (95 % CI) incident ROA hip 2.42 (1.15 to 5.06) *p* = 0.02, incident severe ROA or THR 3.67 (1.68 to 8.01) *p* <0.0001	+ Elevated alpha angle is associated with incident end-stage OA hip	High (67)
Agricola 2013 [148]	Baseline 2D centre edge angle (acetabular shape): 25 ° <normal <40 °, undercoverage <25 °, overcoverage >40 ° (C)	Incidence within 5 years of: 1. ROA hip (KL >1), 2. end-stage OA (KL >2 or THR)	Age, sex, BMI, KL grade	OR (95 % CI) overcoverage 0.52 (0.19 to 1.43) *p* = 0.21, undercoverage 3.64 (1.91 to 6.99) *p* = 0.00	OR (95 % CI) overcoverage 0.34 (0.13 to 0.87) *p* = 0.025, undercoverage 5.45 (2.40 to 12.34) *p* = 0.00	Overcoverage is protective against OA incidence (−). Undercoverage is associated with greater odds of OA incidence and end-stage OA (+)	High (67)
2D and 3D hip bone shape cross-sectional studies							
Gosvig 2010 [149]	Categorical hip 2D deformity: 1. normal, 2.‘pistol grip’, 3) deep acetabular socket (C)	Presence of radiographic hip OA (JSW ≤2 mm) (C)	Age, sex, BMI, other hip deformities	NR	RR (95 % CI) pistol grip 2.2 (1.7 to 2.8) *p* <0.001, deep acetabular socket 2.4 (2.0 to 2.9) *p* <0.001, normal (*p* >0.05)	+	Low (50)
Reichenbach 2011 [27]	The presence or absence of any 3D semi-quantitative MRI-defined cam-deformity (C)	Combined femoral and acetabular cartilage thickness (C)	Age, BMI (NB all participants were young men)	Unadjusted mean cartilage thickness difference with CAM deformity −0.24 mm (95 % CI −0.46 to −0.03)	Adjusted mean cartilage thickness difference with CAM deformity −0.19 mm (95 % CI −0.41 to 0.02)	NC	High (64)
2D hip bone shape case control studies							
Doherty 2008 [43]	Non-spherical femoral head 2D shape assessment: 1. appearance of ‘pistol grip deformity’ (C), 2. maximum femoral head diameter ratio to minimum parallel femoral neck diameter (C)	Presence of radiographic hip OA (JSW ≤2.5 mm) (C)	Age, sex, BMI, BMD, physical activity, history of hip injury, type 3 hand (index finger shorter than ring finger), hand nodes, and center-edge angle	OR (95 % CI) pistol grip deformity 5.75 (4.00 to 8.27). Femoral head-to-neck ratio 10.45 (7.16 to 15.24)	OR (95 % CI) pistol grip deformity 6.95 (4.64 to 10.41). Femoral head-to-neck ratio 12.08 (8.05 to 18.15)	+	Low (53)
Barr 2012 [150]	2D Shape measures of centre-edge angle (acetabular shape) (C)	THR vs no radiographic progression over 5 years (L)	Age, gender, BMI KL grade, use of walking stick, WOMAC function, duration of pain	OR (95 % CI) mode 2 0.74 (0.50 to 1.10) *p* >0.05	OR (95 % CI) Mode 2 0.17 (0.04 to 0.71) p <0.05	NB, this model association is inverse and correlates with acetabular shape	High (76)
Nicholls 2011 [39]	CAM deformity; mean modified triangular index height, alpha angle. 2D acetabular dysplasia; mean lateral center edge angle, (C)	Total hip replacement (L)	BMI, age	OR (*p* value) triangular index 1.131 (0.021). Alpha angle 1.056 (<0.0005). Centre edge angle 0.906 (0.004)	OR (*p* value) Triangular index 1.291 (0.011). Alpha angle 1.057 (<0.0005). Centre- edge angle 0.887 (0.002)	+ Association of hip replacement with CAM impingement and acetabular dysplasia indicated by these results	High (71)
Ankle scintigraphic subchondral bone cross-sectional studies							
Kraus 2013 [60]	Ipsilateral late phase bone scintigraphy, retention presence in tibiotalar joint (C)	Tibiotalar ROA KL grade and JSN (C)	Age, gender, BMI	NR	KL grade *r* = 0.49, *p* <0.0001. JSN *r* = 0.35, *p* <0.0001	+	High (71)
Knupp 2009 [24]	Late phase bone scintigraphy, semi-quantitative retention scoring of tibiotalar joint (C)	Tibiotalar ankle joint JSN. (modified Takakura score) (C)	Nil	0.62 to 0.75 (*p* <0.01)	NR	+	Low (57)

Positive correlation was reported between bone feature and outcome measure (+); negative correlation reported between bone feature and outcome measure (−).*BMD* bone mineral density, *BML* bone marrow lesion, *C* a feature or outcome described in cross-section, *CT* computed tomography, *DXA* dual-energy x-ray absorptiometry, *HOAMS* Hip osteoarthritis MRI scoring system, *IPJ* interphalangeal joint, *JSN* joint space narrowing, *JSW* joint space width, *KL* Kellgren-Lawrence, *L* a feature or outcome described longitudinally, *NA* no association, *NC* no conclusion could be found for an association between bone feature and outcome measure, *MRI* magnetic resonance imaging, *PFJ* patellofemoral joint, *ROA* radiographic osteoarthritis, *OA* osteoarthritis, *OARSI* Osteoarthritis Research Society International, *OR* odds ratio, *RR* relative risk, *TFJ* tibiofemoral joint, *THR* total hip replacement, *TKR* total knee replacement, *VAS* visual analogue scale, *WOMAC* Western Ontario and McMaster Universities arthritis index, *WORMS* whole-organ magnetic resonance imaging score

#### Bone marrow lesions

MRI (one case series, two cross-sectional studies): one well-adjusted, high quality analysis of a prospective OA case series, found that increasing BML number and size in the interphalangeal joints at baseline conferred greater odds of structural progression of OA [141]. Two adjusted cross-sectional analyses found increasing BML number and size scores were associated with increasing severity of structural progression [142, 143]. In summary, BMLs are independently associated with structural progression of hand OA.

#### Osteophyte attrition and cysts

One cross-sectional, adjusted analysis found greater MRI attrition or MRI osteophyte number and size was associated with greater structural severity [142]. However, greater presence of cysts observed on MRI was not associated with greater structural severity of OA [142]. In summary, osteophytes and attrition, but not cysts, are associated with structural severity of hand OA.

### Relationship between hand bone feature and pain

The association between bone features and pain is described in Tables 4 and 5.

**Table 4:** Hand and hip pain associations by feature and quality score

Author	Feature (method)	Pain outcome	Adjustment for confounders	Association (magnitude) crude	Association (magnitude) adjusted	Association	Quality score (%)
Hand MRI bone marrow lesion case series							
Haugen 2014 Abstract [144]	Sum scores (0–48) for BMLs (Oslo hand OA MRI score) (C)	AUSCAN pain scale (L)	Age, sex, BMI, follow-up time	NR	*β* = −0.26, 95 % CI −0.55 to 0.03	NA	High (61)
Hand MRI bone marrow lesion cross-sectional studies							
Haugen 2012 [145]	BML (Oslo MRI hand score) (C) IPJs sum scores	AUSCAN pain scale (C)	Age, sex	NR	OR (95 % CI) 0.96 (0.82 to 1.12)	NA	High (64)
Hand MRI osteophyte cross-sectional studies							
Haugen 2012 [145]	Osteophyte (Oslo MRI hand score) (C) IPJs sum scores	AUSCAN pain scale (C)	Age, sex	NR	OR (95 % CI) 1.04 (0.98 to 1.10)	NA	High (64)
Hand MRI attrition cross-sectional studies							
Haugen 2012 [145]	Attrition (Oslo MRI hand score) (C) IPJs sum scores	AUSCAN pain scale (C)	Age, sex	NR	OR (95 % CI) 1.15 (0.98 to 1.34)	NA	High (64)
Hand MRI subchondral cyst cross-sectional studies							
Haugen 2012 [145]	Cyst (Oslo MRI hand score) (C) IPJs sum scores	AUSCAN pain scale (C)	Age, sex	NR	OR (95 % CI) 0.93 (0.56 to 1.55)	NA	High (64)
Hand scintigraphy subchondral bone cross-sectional studies							
Macfarlane 1993 [61]	Late phase isotope bone scan small joints of the hand (C)	Hand pain VAS (C)	Nil	Correlation coefficient 0.06, *p* = 0.304	NR	NA	Low (57)
Hip MRI bone marrow lesion cross-sectional studies							
Kumar 2013 [151]	Total hip semi-quantitative BML score (C)	Self-reported hip pain HOOS score (C)	Nil	NR	p correlation −0.29 (*p* <0.01)	A higher BML score means a lower or worse HOOS pain score	High (71)
Maksymowych 2014 [152]	Semi-quantitative BML HIP (HOAMS) (C)	Baseline WOMAC pain (C)	Nil	*p* <0.001	NR	+	High (64)
Hip MRI subchondral cyst cross-sectional studies							
Kumar 2013 [151]	Total hip semi-quantitative subchondral cyst score (C)	Self-reported hip pain HOOS score (C)	Nil	NR	p correlation −0.37 (*p* <0.001)	A higher cyst score means a lower or worse HOOS pain score	High (71)

Positive correlation reported between bone feature and outcome measure (+); negative correlation reported between bone feature and outcome measure (−). *AUSCAN* Australian/Canadian Osteoarthritis hand index, *BML* bone marrow lesion, *C* a feature or outcome described in cross-section, *chronic pain* knee pain on most days for at least the last month, *HOAMS* Hip osteoarthritis MRI scoring system, *HOOS* Hip dysfunction and osteoarthritis outcome score, *IPJ* interphalangeal joint, *L* a feature or outcome described longitudinally, *NA* no association, *NR* not recorded, *OA* osteoarthritis, *OR* odds ratio, *VAS* visual analogue scale

**Table 5:** The summary subchondral bone associations with joint replacement, structural progression and pain in peripheral OA

Subchondral bone feature of OA	Pain and structural associations						
	Knee structure	Knee pain	Hand structure	Hand pain	Hip structure	Hip pain	Ankle structure
MRI bone marrow lesions	Progression (i)	LPS (i)	Progression (i)	No LPS (w)	Severity (w)	Severity (n)	
		IFP (n)					
				No severity (n)			
	TKR (i)						
MRI osteophytes	Progression (i)	LPS (n)	Severity (n)	No severity (n)			
	TKR (n)						
MRI bone attrition	No progression (0)	No severity (0)	Severity (n)	No severity (n)			
MRI bone shape or dimensions	Progression (i)	IFP (i)			No severity (0)		
		Severity (n)					
	TKR (i)						
MRI bone cyst	No progression	No LPS (n)	No severity (n)	No severity (n)		Severity (n)	
	?severity						
MRI or CT trabecular morphometry	Progression (n)				Severity (n)		
DXA or CT Peri-articular BMD	Progression (n)				Severity (w)		
2D Bone shape	Severity (w)	Severity (n)			Progression (i)		
					THR (i)		
Scintigraphy	No Progression (0)			No severity (n)			Severity (w)

*CT* computed tomography, dual-energy *DXA* x-ray absorptiometry, *(i)* independent association, *IFP* incident frequent pain, *(n)* association with no or inadequate covariate adjustment, *TKR* total knee replacement, *THR* total hip replacement, *LPS* mean change in longitudinal pain severity, *(w)* well-adjusted association, *(0)* association insignificant after covariate adjustment

#### Bone marrow lesions

MRI (one case series, one cross-sectional study): one well-adjusted, high quality analysis of a prospective OA case series, found that BML number and size at baseline was not associated with longitudinal change in hand pain [144]. One adjusted cross-sectional analysis found no association of BMLs with severity of pain [145]. In summary, BMLs are not independently associated with longitudinal or cross-sectional severity of pain.

#### Osteophyte attrition and cysts

One cross-sectional, adjusted analysis found no association between bone features, osteophytes, attrition or cysts observed on MRI, and pain severity [145]. In summary, osteophytes, attrition and cysts are not associated with severity of hand pain.

#### Scintigraphy

Scintigraphy (one cross-sectional study): one cross-sectional unadjusted analysis found no significant association between bone signal in the hands and severity of pain. In summary, bone scintigraphy signal is not associated with severity of pain in hand OA.

### Relationship between hip bone feature and structural progression

The association between bone features, and structural progression and joint replacement is described in Tables 3 and 5.

#### Bone marrow lesions

MRI (two cross-sectional studies): one well-adjusted [69] and one unadjusted [46] cross-sectional analysis both found that BMLs were associated with greater structural severity. In summary, BMLs are associated with structural severity of hip OA.

#### Trabecular bone morphometry

One unadjusted cross-sectional analysis found greater MRI bone volume fraction, trabecular thickening, trabecular number and lower trabecular spacing were associated with greater structural severity of OA [33]. In summary, bone volume fraction, trabecular thickening, number and spacing are associated with structural severity in hip OA.

#### Peri-articular bone mineral density

DXA (two cross-sectional studies): one well-adjusted [30] and one adjusted [32] cross-sectional analysis found greater BMD was associated with greater structural severity. In summary, BMD is associated with structural severity of hip OA.

#### 2D and 3D hip bone shape

Hip bone shape (three cohort, two cross-sectional, three case–control studies): in two prospective cohort, well-adjusted, high quality analyses increasing asphericity of the femoral head (measured as an elevated alpha angle, or in shape modes 11 and 15) was associated with total hip replacement (THR) [146] or with structural progression and THR [147] respectively. In one prospective cohort, well-adjusted, high quality analysis, acetabular undercoverage of the femoral head (a low centre-edge angle) was associated with structural progression or THR [148]. In one well-adjusted cross-sectional analysis, 2D asphericity deformity of the femoral head (cam-type deformity) was associated with structural severity [149]. In one well-adjusted cross-sectional analysis of MRI-determined femoral head asphericity in asymptomatic young men, there was a significantly lower cartilage thickness in those with than those without any detectable asphericity. This became insignificant after covariate adjustment [27]. Case–control analyses identified the same associations as the cohort analyses [39, 43, 150]. In summary, asphericity of the femoral head and acetabular undercoverage of the femoral head are independently associated with structural progression and THR.

### Relationship between hip bone feature and pain

The association between bone features and pain is described in Tables 4 and 5.

#### Bone marrow lesions

MRI (two cross-sectional studies): two cross-sectional, unadjusted analyses found that increasing semi-quantitative BML scores were associated with greater severity of pain [151, 152]. In summary, BMLs are associated with severity of pain in hip OA.

#### Bone cyst

One cross-sectional, unadjusted analysis found that increasing semi-quantitative cyst scores on MRI were associated with greater severity of pain [151]. In summary: cysts are associated with severity of pain in hip OA.

### Relationship between ankle bone features and structural progression

The association between bone features and structure is described in Table 3 and 5.

#### Scintigraphy

Scintigraphy (two cross-sectional studies): one well-adjusted [59] and one unadjusted [62] cross-sectional analysis found the presence or semi-quantitative scoring of late-phase bone signal in the tibiotalar joint was associated with greater structural severity. In summary, bone scintigraphy signal is associated with ankle structural severity.

## Discussion

This systematic review is the first to have incorporated quality scoring alongside statistical adjustment in the comprehensive examination of the relationship of subchondral bone pathology with both structural progression of OA and pain for all non-conventional types of radiographic imaging of peripheral joints with OA. This systematic review has concluded that there are independent associations between imaging-assessed bone pathology and structural progression and pain in the knee, hand, and hip.

Subchondral bone pathology may lead to cartilage degeneration by altering the biomechanical force distribution across joint cartilage, or disruption of the osteochondral junction and release of soluble biomediators influencing the cartilage [153, 154]. In OA the homeostatic process of subchondral bone remodeling fails, leading to increased bone turnover, volume and change in stiffness and shock-absorbing capacity [155–157]. BMLs histologically represent increased bone turnover [158]. Cartilage overlying altered bone has been observed to have greater damage than healthy bone in knees from human cadavers [159]. That study, and an excluded study [160], concur with the independent association between BMLs, and structural progression of OA in knees and hands and total knee replacement, as concluded by this analysis. Although randomised control trials were not excluded from this review, several such trials were excluded on the basis of failure to formally quantify any correlation between BMLs and structural progression outcomes. These include the strontium [161], intensive weight-loss therapy [162] and glucosamine [163] trials, and some of these describe a concordant reduction in BML size and cartilage volume loss.

Osteophytes represent subchondral bone hypertrophy typical of OA. They represent endochondral and direct bone formation and create a circumferential increase in bone area around each knee cartilage plate, particularly on the medial side in OA [118], which concurs with the independent association between osteophytes demonstrated on MRI and structural progression as observed in this analysis.

In terms of bone morphology, knee OA is associated with shallow trochlear patellar grooves in multiple epiphyseal dysplasia [164]. These findings concur with the findings of Stefanik and Kalichman, and colleagues in studies of knee OA in this review [115, 116, 165]. Anterior-cruciate ligament (ACL) rupture represents a risk factor for developing knee OA. In cases of ACL tear in previously normal knees of young healthy adults, the 3D shape of the femur, tibia and patella expands more rapidly than in controls without radiographic evidence of knee OA in the subsequent 5 years [166]. The 3D shape of the same knee bones has also been associated with the outcome of joint replacement [167]. This highlights the importance of bone shape and concurs with our conclusion that 3D knee shape and 2D hip shape are independently associated with structural progression of OA and total joint replacement.

We found that bone attrition and cysts were associated with structural progression or severity, but not after covariate adjustment, which included other OA subchondral bone features. This suggests these bone features are an epiphenomenon of the pathogenic process of structural progression rather than a primary cause. This hypothesis is supported by bone cysts and attrition frequently occurring synchronously with BMLs [88, 138] and incident bone attrition has been strongly associated with the presence of BMLs within the same compartment [168].

Increasing bone volume fraction, trabecular number and thickness, but decreasing trabecular spacing on CT and MRI studies were associated with structural progression. These specific associations concur with numerous histological analyses of peripheral joint OA [169–171].

Subchondral bone, particularly BMLs, have been found to be associated with pain in knee, hip and hand OA. However, some analyses, in which pain was measured using heterogenous pain outcomes, report an absence of longitudinal or cross-sectional association with BMLs [101, 172, 173]. Furthermore, previous systematic reviews have concluded moderate association at the most between BMLs and knee pain [7, 14]. With the benefit of incorporating more well-adjusted analyses in this systematic review, we have highlighted that BMLs are independently longitudinally associated with change in severity of pain, but are only associated with incident frequent knee pain. In analyses excluded from the current review, incident knee BMLs predicted incident knee pain in healthy community-based adults at risk of OA [174]. Concurrent trends in reduction of pain and BML size were observed in the zoledronic acid trial [175] and the intensive diet and exercise for arthritis trial [176]. These were not included because they did not make a formal comparison of pain and BMLs. The mechanism by which BMLs may cause pain is unknown but may include subchondral microfractures, angina from a decreased blood supply causing ischaemia, and raised intraosseous pressure [177–179].

The independent association between a mismatch of the femoral and tibial articulating surface areas and incident frequent knee symptoms indicates that bone shape may predict not only the incidence of radiographic knee OA [120], but also symptomatic OA.

In terms of limitations, stratifying observational studies by quality may artificially create relatively high quality studies from a collection of generally low quality studies. However the distribution and summary statistics of quality scores indicate a suitably broad range of quality, particularly in the influential cohort studies with a mean of 54 % and range of 22−83 %. The decision to exclude articles reporting analysis of association that included fewer than 20 patients with OA may seem arbitrary. However, several papers report associations with the presence or absence of pain or structural progression based upon small numbers of patients. Our threshold decision reflects the absence of specific guidelines on how to exclude such papers, with inherent risk of imprecision, in the context of heterogenous populations and statistical analyses. Had these papers been included there would have been no change in any of the conclusions in Table 5 (data not shown). The use of joint replacement as an outcome measure has a number of limitations including the effect of patient willingness, variation in orthopaedic opinion, availability of health services and health insurance, and therefore may be influenced depending upon the country and context in which the study is performed.

Publication bias could not be assessed with a funnel plot as there were insufficient results for odds and relative risk ratios. The heterogenous nature of the measures of bone features and structural or pain outcomes precluded a meta-analysis or calculation of an effect size. This was because there were insufficient analyses describing the same association between the same bone features and outcome measure pair.

## Conclusions

In conclusion subchondral bone plays an integral role in the pathogenesis of OA. BMLs, osteophytes identified on MRI and tibial bone area are independently associated with structural progression of knee OA. BMLs and tibial bone area are independently associated with TKR. BMLs are independently associated with structural progression of hand OA and 2D hip bone shape is associated with progression of structural hip OA and THR. BMLs are independently associated with longitudinal change in severity of pain and femorotibial articulating area mismatch is independently associated with incident frequent knee pain. These bone features may be used in the future for targeting treatment, stratifying patients into those most in need of OA modification and measuring treatment response.

## Additional file

Additional file 1: **Supplementary material: supplementary methods and results.** (DOC 1659 kb)

## Abbreviations

2D: two-dimensional
3D: three-dimensional
ACL: anterior cruciate ligament
BLOKS: Boston–Leeds osteoarthritis knee score
BMD: bone mineral density
BMI: body mass index
BML: bone marrow lesion
BOKS: Boston osteoarthritis of the knee study
BVF: bone volume fraction
Cam: a resemblance to a camshaft
CRP: C-reactive protein
CT: computed tomography
DMOAD: disease-modifying osteoarthritis drug
DXA: dual-energy x-ray absorptiometry
EMBASE: Excerpta Medica database
GARP: Genetics, osteoarthritis and progression study
HOAMS: Hip osteoarthritis MRI scoring system
IPJ: interphalangeal joint
JSN: joint space narrowing
JSW: joint space width
KL: Kellgren-Lawrence
KOSS: knee osteoarthritis scoring system
MOST: multicentre osteoarthritis study
MRI: magnetic resonance imaging
NA: no association
NC: no conclusion
OA: osteoarthritis
OAI: Osteoarthritis Initiative
OARSI: Osteoarthritis Research Society International
OR: odds ratio
PET: positron emission tomography
PFJ: patellofemoral joint
PRISMA: Preferred reporting items for systematic reviews and meta-analyses
qCT: quantitative computed tomography
ROA: radiographic osteoarthritis
RR: relative risk ratio
SSR: subchondral surface ratio
THR: total hip replacement
TFJ: tibiofemoral joint
TKR: total knee replacement
VAS: visual analogue scale
WOMAC: Western Ontario and McMaster Universities arthritis index
WORMS: whole-organ magnetic resonance imaging score
