# Supplementary material for: A systematic review of the relationship between subchondral bone features, pain and structural pathology in peripheral joint osteoarthritis
Source: Arthritis Res Ther. 2015 Aug 25;17(1):228. doi: 10.1186/s13075-015-0735-x (PMC4548899; doi:10.1186/s13075-015-0735-x)
Supplement: Supplementary file 1 — Supplementary material: supplementary methods and results. (DOC 1659 kb) [file 13075_2015_735_MOESM1_ESM.doc]

# SUPPLEMENTARY MATERIAL

# Supplementary Methods

## Systematic Search (Supplementary Table 1)

A search of the Medline, EMBASE and Cochrane library databases was performed for original articles reporting relationships between non-radiographic imaging-assessed subchondral bone pathologies joint replacement, pain or structural progression in knee, hip, hand, ankle and foot OA

The bone pathological changes include bone marrow lesions (BMLs), osteophytes, attrition, cysts, as well as changes in shape, bone mineral density, bone morphometry (bone volume fraction, trabecular number, spacing and thickness), and bone signal from positron emission tomography and scintigraphy.

## Data extraction

Papers meeting the inclusion/exclusion criteria were divided into longitudinal and cross-sectional papers. Longitudinal papers included prospective and retrospective cohorts and case-control studies with longitudinal data (i.e. nested case control studies). Extracted data included (a) patient demographics (age, sex and body mass index) (b) OA (clinical, radiographic or diagnostic) classification used, with the definition and prevalence of radiographic OA, (c) subchondral bone pathology feature, (d) joint replacement, pain or structural progression outcome measure (e) presence/absence of a relationship between feature and outcome (f) statistical results with or without adjustment for confounders and (g) the ipsicompartmental or contralateral compartment structural progression in relation to the bone pathology in longitudinal studies.

## Quality Assessment (Supplementary Table 2)

A standardised quality scoring tool, previously used in other similar systematic reviews was adapted to assess the following components: (a) study population, (b) MRI subchondral bone feature, (c) pain or structural progression outcome, (d) study design and (e) analysis and data presentation (Supplementary Table 2). A score of ‘1’ or ‘0’ was allocated for each question according to whether the study fulfilled the criteria or not respectively. Where multiple bone features were assessed per article (e.g. criteria 11) the mean score was used. Any discordance in opinion was recorded and where consensus could not be achieved a third reviewer (PC) was consulted. The number of criteria applied to each type of study (e.g. cohort n=18 and cross-sectional n=14) varied and therefore scores were compared as percentages of the maximum score. A study was considered to be high quality if it exceeded or equaled the mean score in its class.

Most studies selected patients from existing cohorts rather than from the general population (criteria 1) and patients were selected by a minimum of evidence of OA (e.g. KL≥2) rather than at a uniform stage of OA severity (e.g. KL=2) (criteria 2). Most studies did not provide evidence of assessing the bone image feature before the knee OA outcome (criteria 9) and similarly did not indicate a prospective analysis plan for the relationship between bone image feature and OA outcome (criteria 17).

## Best evidence synthesis

An association of a bone feature with a longitudinal OA outcome (structural progression, longitudinal change in pain, incident pain or joint replacement) was determined from cohort studies only. If a prospective cohort study analysis was of above average quality and found a statistically significant association between a bone feature and a longitudinal outcome after adjustment for at least age, gender and body mass index (referred to in the text as ‘well-adjusted’) this association was referred to as an ‘independent’ association. These three criteria were determined for all longitudinal analyses and if any of these three criteria were not fulfilled, the association was referred to simply as an association. The validity of cross-sectional associations was determined using cross-sectional and case-control studies and establishing whether the analysis of the association of severity was well-adjusted or not. This data is summarised in Table 5 of the main manuscript.

# Supplementary Table 1

## EMBASE (1980 to September 2014):

| 1 | osteoarthri* | 42 | 25 or 26 or 27 or 28 or 29 or 30 or 31 or 32 or 33 or 34 or 35 or 36 or 37 or 38 or 39 or 40 or 41 |
| --- | --- | --- | --- |
| 2 | osteoarthro* | 43 | "magnetic resonance imag*".ti,ab |
| 3 | (arthri* adj2 degenerative) | 44 | mri.ti,ab |
| 4 | exp OSTEOARTHRITIS | 45 | mr.ti,ab |
| 5 | 1 or 2 or 3 or 4 | 46 | "magnetic resonance".ti,ab |
| 6 | knee*.ti,ab | 47 | NUCLEAR MAGNETIC RESONANCE IMAGING/ |
| 7 | KNEE/ | 48 | 43 or 44 or 45 or 46 or 47 |
| 8 | hand*.ti,ab | 49 | 24 AND 42 and 48 |
| 9 | HAND/ | 50 | DEXA |
| 10 | Hip*.ti,ab | 51 | DXA |
| 11 | HIP/ | 52 | DUAL ENERGY X RAY ABSORPTIOMETRY |
| 12 | foot* | 53 | 50 OR 51 or 52 |
| 13 | exp FOOT/ | 54 | "bone mineral density" |
| 14 | ankle* | 55 | BMD |
| 15 | exp ANKLE/ | 56 | BONE DENSITY/ |
| 16 | 6 or 7 or 8 or 9 or 10 or 11 or 12 or 13 or 14 or 15 | 57 | 54 or 55 or 56 |
| 17 | 5 and 16 | 58 | 24 and 53 and 57 |
| 18 | HAND OSTEOARTHRITIS | 59 | "Computed tomography" |
| 19 | HIP OSTEOARTHRITIS | 60 | CT |
| 20 | coxarthr*.ti,ab | 61 | COMPUTER ASSISTED TOMOGRAPHY |
| 21 | KNEE OSTEOARTHRITIS/ | 62 | "micro-computed tomography" |
| 22 | gonarthr*.ti,ab | 63 | pQCT |
| 23 | 18 or 19 or 20 or 21 or 22 | 64 | HR-pQCT |
| 24 | 17 or 23 | 65 | 59 or 60 or 61 or 62 or 63 or 64 |
| 25 | "subchondral bone".ti,ab | 66 | 57 or 42 |
| 26 | bml.ti,ab | 67 | 24 and 65 and 66 |
| 27 | "bone marrow lesion*".ti,ab | 68 | PET |
| 28 | "bone marrow oedema".ti,ab | 69 | 24 and 68 |
| 29 | "bone marrow edema".ti,ab | 70 | scintigraphy |
| 30 | BONE MARROW EDEMA/ | 71 | 24 and 70 |
| 31 | osteophyte*.ti,ab | 72 | Bone shape |
| 32 | OSTEOPHYTE/ | 73 | 24 and 72 |
| 33 | "bone cyst*".ti,ab | 74 | 49 or 58 or 67 or 69 or 71 or 73 |
| 34 | BONE CYST/ | 75 | 74 limit to humans |
| 35 | "bone area*".ti,ab |  |  |
| 36 | "bone shape".ti,ab |  |  |
| 37 | "bone attrition".ti,ab |  |  |
| 38 | "trabecular".ti,ab |  |  |
| 39 | TRABECULAR BONE/ |  |  |
| 40 | "volume fraction".ti,ab |  |  |
| 41 | "BV/TV".ti,ab |  |  |

## Medline (1950 to September 2014):

| 1 | osteoarthri* | 40 | "magnetic resonance imag*" |
| --- | --- | --- | --- |
| 2 | osteoarthro* | 41 | mri |
| 3 | (arthri* adj2 degenerative) | 42 | mr |
| 4 | exp OSTEOARTHRITIS | 43 | "magnetic resonance" |
| 5 | 1 or 2 or 3 or 4 | 44 | MAGNETIC RESONANCE IMAGING/ |
| 6 | knee* | 45 | 40 OR 41 or 42 OR 43 OR 44 |
| 7 | knee/ OR exp KNEE JOINT/ | 46 | 23 AND 39 and 45 |
| 8 | hand* | 47 | DEXA |
| 9 | hand/ OR exp HAND joint | 48 | DXA |
| 10 | Hip* | 49 | Absorbtiometry, photon/ |
| 11 | hip/ OR exp HIP JOINT | 50 | 47 or 48 or 49 |
| 12 | foot*.ti,ab | 51 | "bone mineral density" |
| 13 | foot/ OR exp FOOT JOINTS/ | 52 | BMD |
| 14 | ankle*.ti,ab | 53 | BONE DENSITY/ |
| 15 | ankle / OR exp ANKLE JOINT/ | 54 | 51 OR 52 or 53 |
| 16 | 6 or 7 or 8 or 9 or 10 or 11 or 12 or 13 or 14 or 15 | 55 | 23 AND 50 and 54 |
| 17 | 5 and 16 | 56 | "Computed tomograph*" |
| 18 | osteoarthritis hip | 57 | CT |
| 19 | coxarthr* | 58 | tomography, X-RAY COMPUTED/ |
| 20 | osteoarthritis knee | 59 | **"micro-computed tomography"** |
| 21 | gonarthr* | 60 | pQCT |
| 22 | 18 or 19 or 20 or 21 | 61 | HR-pQCT |
| 23 | 17 or 22 | 62 | 56 or 57 or 58 or 59 or 60 or 61 |
| 24 | "subchondral bone" | 63 | 54 or 39 |
| 25 | bml | 64 | 23 and 62 and 63 |
| 26 | "bone marrow lesion*" | 65 | PET |
| 27 | "bone marrow oedema" | 66 | 23 and 65 |
| 28 | "bone marrow edema" | 67 | Scintigraphy |
| 29 | osteophyte* | 68 | 23 and 67 |
| 30 | OSTEOPHYTE/ | 69 | Bone shape |
| 31 | "bone cyst*" | 70 | 23 and 69 |
| 32 | BONE CYSTS/ | 71 | 46 or 55 or 64 or 66 or 68 or 70 |
| 33 | "bone area*" | 72 | 71 limit to humans |
| 34 | "bone shape" |  |  |
| 35 | "bone attrition" |  |  |
| 36 | trabecular |  |  |
| 37 | "volume fraction" |  |  |
| 38 | "BV/TV" |  |  |
| 39 | 24 OR 25 OR 26 OR 27 OR 28 OR 29 OR 30 OR 31 OR 32 OR 33 OR 34 OR 35 OR 36 OR 37 OR 38 |  |  |

# Supplementary Table 2 – Quality scoring tool

| Item | Criterion | CC | CH | CS |
| --- | --- | --- | --- | --- |
| Study population | | | | |
| 1 | Recruitment from the general population | 1 | 1 | 1 |
| 2 | Selection occurred before disease onset or at a uniform point.  A uniform point was considered to be equal baseline grade of structural progression (e.g. Kellgren Lawrence grade) or an analysis within the same osteoarthritic joint | 1 | 1 | 1 |
| 3 | Cases and controls drawn were from the same population | 1 |  |  |
| 4 | Participation rate >80% for cohort studies (retrospective cohort studies score zero automatically) |  | 1 |  |
| 5 | Sufficient description of baseline characteristics - must include age, gender and BMI (or height and weight) | 1 | 1 | 1 |
| 6 | Baseline characteristics comparable between cases and controls - must include age, gender and BMI (or height and weight) | 1 |  |  |
| Assessment of Imaging-detected subchondral bone risk factor or feature | | | | |
| 7 | Risk factor / feature assessed with a standardised method (e.g. WORMS BML scoring or an automated calculation of bone area but not a subjective opinion of a radiologist on the presence of bone attrition) | 1 | 1 | 1 |
| 8 | Risk factor / feature assessment was identical (performed the same way) in the studied population(s) | 1 | 1 | 1 |
| 9 | Risk factor / feature was assessed prior to the outcome (structural progression or pain). A score of zero was allocated if the methods did not describe this. | 1 | 1 | 1 |
| Assessment of joint OA outcome (pain or structural progression) | | | | |
| 10 | Outcome assessment was identical in the studied population(s) | 1 | 1 | 1 |
| 11 | Outcomes were assessed reproducibly (intraclass correlation coefficient > 0.81 with a standardised assessment). If multiple outcomes were measured the mean reproducibility score was used. | 1 | 1 | 1 |
| 12 | Outcome classification was standardised (e.g. the WOMAC pain score but not a subjective opinion of a patient’s pain) | 1 | 1 | 1 |
| Study design | | | | |
| 13 | Prospective study design used |  | 1 |  |
| 14 | Follow up time > 3 years | 1 | 1 |  |
| 15 | Information provided on completers vs withdrawls in cohorts (without prospective trial data cohorts automatically score zero) |  | 1 |  |
| 16 | Outcome evaluators were blinded to feature (risk factor) | 1 | 1 | 1 |
| 17 | Analysis of relationship between feature and outcome was planned prospectively | 1 | 1 | 1 |
| Analysis and data presentation | | | | |
| 18 | The frequency of most important outcomes were given | 1 | 1 | 1 |
| 19 | appropriate analysis techniques used (statistical or comparative techniques) | 1 | 1 | 1 |
| 20 | adjusted for at least age, BMI and gender | 1 | 1 | 1 |
| Maximum Score | | 17 | 18 | 14 |

CC: case control, CH cohort (prospective and retrospective), CS: cross sectional

# Supplementary Tables 3: Quality Scoring Results Cross-sectional studies

|  |  | Quality Scoring Criteria | | | | | | | | | | | | | | | | | | | | | | | | | | |  | |
| --- | --- | --- | --- | --- | --- | --- | --- | --- | --- | --- | --- | --- | --- | --- | --- | --- | --- | --- | --- | --- | --- | --- | --- | --- | --- | --- | --- | --- | --- | --- |
| No. | Cross-sectional Study | 1 | 2 | 3 | 4 | | 5 | | 6 | | 7 | | 8 | 9 | 10 | | 11 | | 12 | 13 | 14 | 15 | 16 | 17 | 18 | 19 | 20 | Total | | % |
| 1 | Ai 2010 | 0 | 0 |  |  | | 0 | |  | | 1 | | 1 | 0 | 1 | | 1 | | 1 |  |  |  | 1 | 0 | 1 | 1 | 0 | 8 | | 57% |
| 2 | Akamatsu 2014 | 0 | 0 |  |  | | 1 | |  | | 1 | | 1 | 0 | 1 | | 0 | | 1 |  |  |  | 1 | 0 | 1 | 1 | 0 | 8 | | 57% |
| 3 | Antoniades 2000 | 0 | 0 |  |  | | 1 | |  | | 1 | | 1 | 0 | 1 | | 1 | | 1 |  |  |  | 1 | 0 | 1 | 1 | 0 | 9 | | 64% |
| 4 | Baranyay 2007 | 0 | 1 |  |  | | 1 | |  | | 1 | | 1 | 0 | 1 | | 1 | | 1 |  |  |  | 0 | 0 | 1 | 1 | 1 | 10 | | 71% |
| 5 | Bilgici 2010 | 0 | 0 |  |  | | 1 | |  | | 1 | | 1 | 0 | 1 | | 1 | | 1 |  |  |  | 1 | 0 | 0 | 1 | 0 | 8 | | 57% |
| 6 | Burnett 2012 | 0 | 0 |  |  | | 0 | |  | | 1 | | 1 | 0 | 1 | | 1 | | 1 |  |  |  | 1 | 0 | 1 | 1 | 0 | 8 | | 57% |
| 7 | Chaganti 2010 | 0 | 0 |  |  | | 1 | |  | | 1 | | 1 | 0 | 1 | | 0 | | 1 |  |  |  | 1 | 0 | 1 | 1 | 1 | 9 | | 64% |
| 8 | Chiba 2011 | 0 | 0 |  |  | | 0 | |  | | 1 | | 1 | 1 | 1 | | 0 | | 1 |  |  |  | 1 | 0 | 1 | 1 | 0 | 8 | | 57% |
| 9 | Chiba 2012 | 0 | 0 |  |  | | 1 | |  | | 1 | | 1 | 0 | 1 | | 0 | | 1 |  |  |  | 1 | 0 | 1 | 1 | 0 | 8 | | 57% |
| 10 | Crema 2010 | 0 | 0 |  |  | | 1 | |  | | 1 | | 1 | 0 | 1 | | 1 | | 1 |  |  |  | 0 | 0 | 1 | 0 | 0 | 7 | | 50% |
| 11 | Dawson 2013 abstract | 0 | 0 |  |  | | 1 | |  | | 0 | | 0 | 0 | 0 | | 0 | | 0 |  |  |  | 0 | 0 | 0 | 0 | 1 | 2 | | 14% |
| 12 | Ding 2005 | 0 | 0 |  |  | | 1 | |  | | 1 | | 1 | 0 | 1 | | 1 | | 1 |  |  |  | 0 | 0 | 1 | 1 | 1 | 9 | | 64% |
| 13 | Dore 2009 | 0 | 0 |  |  | | 1 | |  | | 1 | | 1 | 0 | 1 | | 1 | | 1 |  |  |  | 1 | 0 | 1 | 1 | 1 | 10 | | 71% |
| 14 | Driban 2011 | 0 | 0 |  |  | | 1 | |  | | 1 | | 1 | 0 | 1 | | 1 | | 0 |  |  |  | 1 | 0 | 1 | 1 | 1 | 9 | | 64% |
| 15 | Driban 2011 | 0 | 0 |  |  | | 1 | |  | | 1 | | 1 | 0 | 1 | | 1 | | 1 |  |  |  | 1 | 0 | 1 | 1 | 1 | 10 | | 71% |
| 16 | Eckstein 2010 | 0 | 0 |  |  | | 1 | |  | | 1 | | 1 | 0 | 1 | | 0 | | 1 |  |  |  | 0 | 0 | 1 | 0 | 0 | 6 | | 43% |
| 17 | Felson 2001 | 0 | 0 |  |  | | 1 | |  | | 1 | | 1 | 0 | 1 | | 0.5 | | 1 |  |  |  | 0 | 0 | 1 | 1 | 0 | 7.5 | | 54% |
| 18 | Fernandez-Madrid1994 | 0 | 0 |  |  | | 1 | |  | | 0 | | 0 | 0 | 1 | | 0.5 | | 1 |  |  |  | 1 | 0 | 1 | 1 | 0 | 6.5 | | 46% |
| 19 | Frobell 2010 | 0 | 0 |  |  | | 1 | |  | | 1 | | 1 | 0 | 1 | | 0 | | 1 |  |  |  | 0 | 0 | 1 | 1 | 1 | 8 | | 57% |
| 20 | Gosvig 2010 | 0 | 0 |  |  | | 0 | |  | | 1 | | 1 | 0 | 1 | | 0 | | 1 |  |  |  | 0 | 0 | 1 | 1 | 1 | 7 | | 50% |
| 21 | Gudbergsen | 0 | 0 |  |  | | 1 | |  | | 1 | | 1 | 0 | 1 | | 0 | | 1 |  |  |  | 0 | 0 | 1 | 1 | 1 | 8 | | 57% |
| 22 | Guymer 2007 | 0 | 1 |  |  | | 1 | |  | | 1 | | 1 | 0 | 1 | | 1 | | 1 |  |  |  | 0 | 0 | 1 | 1 | 1 | 10 | | 71% |
| 23 | Haugen 2012 | 0 | 0 |  |  | | 1 | |  | | 1 | | 1 | 0 | 1 | | 1 | | 1 |  |  |  | 1 | 0 | 1 | 1 | 0 | 9 | | 64% |
| 24 | Haugen 2013 | 0 | 0 |  |  | | 0 | |  | | 1 | | 1 | 0 | 1 | | 0 | | 1 |  |  |  | 0 | 0 | 1 | 1 | 0 | 6 | | 43% |
| 25 | Haugen 2012 | 0 | 0 |  |  | | 1 | |  | | 1 | | 1 | 0 | 1 | | 1 | | 1 |  |  |  | 1 | 0 | 1 | 1 | 0 | 9 | | 64% |
| 26 | Haverkamp 2011 | 0 | 0 |  |  | | 1 | |  | | 1 | | 1 | 0 | 1 | | 0 | | 1 |  |  |  | 0 | 0 | 0 | 1 | 0.5 | 6.5 | | 46% |
| 27 | Hayashi | 0 | 0 |  |  | | 1 | |  | | 0 | | 1 | 0 | 1 | | 1 | | 1 |  |  |  | 1 | 0 | 1 | 1 | 0 | 8 | | 57% |
| No. | Cross-sectional Study | 1 | 2 | 3 | 4 | | 5 | | 6 | | 7 | | 8 | 9 | 10 | | 11 | | 12 | 13 | 14 | 15 | 16 | 17 | 18 | 19 | 20 | Total | | % |
| 28 | Hayes | 0 | 0 |  |  | | 1 | |  | | 1 | | 1 | 0 | 1 | | 0.5 | | 1 |  |  |  | 1 | 0 | 1 | 1 | 0 | 8.5 | | 61% |
| 29 | Hernandez-Molina 2008 | 0 | 0 |  |  | | 1 | |  | | 1 | | 1 | 0 | 1 | | 1 | | 1 |  |  |  | 1 | 0 | 1 | 1 | 1 | 10 | | 71% |
| 30 | Ip | 0 | 0 |  |  | | 1 | |  | | 1 | | 1 | 0 | 1 | | 0.5 | | 1 |  |  |  | 1 | 0 | 1 | 1 | 1 | 9.5 | | 68% |
| 31 | Jones 2004 | 0 | 0 |  |  | | 0 | |  | | 1 | | 1 | 0 | 1 | | 1 | | 1 |  |  |  | 0 | 0 | 0 | 1 | 1 | 7 | | 50% |
| 32 | Kalichman 2007 | 0 | 0 |  |  | | 1 | |  | | 1 | | 1 | 0 | 1 | | 0 | | 1 |  |  |  | 1 | 0 | 1 | 1 | 1 | 9 | | 64% |
| 33 | Kalichman 2007 | 0 | 0 |  |  | | 1 | |  | | 1 | | 1 | 0 | 1 | | 0 | | 1 |  |  |  | 0 | 0 | 1 | 1 | 1 | 8 | | 57% |
| 34 | Kim | 0 | 0 |  |  | | 0 | |  | | 1 | | 1 | 0 | 1 | | 1 | | 1 |  |  |  | 1 | 0 | 1 | 1 | 1 | 9 | | 64% |
| 35 | Knupp 2009 | 0 | 0 |  |  | | 0 | |  | | 1 | | 1 | 0 | 1 | | 1 | | 1 |  |  |  | 1 | 0 | 1 | 1 | 0 | 8 | | 57% |
| 36 | Kornaat 2006 | 0 | 0 |  |  | | 1 | |  | | 1 | | 1 | 0 | 1 | | 1 | | 1 |  |  |  | 1 | 0 | 1 | 1 | 1 | 10 | | 71% |
| 37 | Kornaat 2005 | 0 | 0 |  |  | | 1 | |  | | 1 | | 1 | 0 | 1 | | 1 | | 1 |  |  |  | 0 | 0 | 1 | 1 | 0 | 8 | | 57% |
| 38 | Kraus 2009 | 0 | 0 |  |  | | 1 | |  | | 1 | | 1 | 0 | 1 | | 1 | | 1 |  |  |  | 1 | 0 | 1 | 1 | 1 | 10 | | 71% |
| 39 | Kraus 2013 | 0 | 0 |  |  | | 1 | |  | | 1 | | 1 | 0 | 1 | | 1 | | 1 |  |  |  | 1 | 0 | 1 | 1 | 1 | 10 | | 71% |
| 40 | Kumar | 1 | 0 |  |  | | 1 | |  | | 1 | | 1 | 0 | 1 | | 1 | | 1 |  |  |  | 1 | 0 | 1 | 1 | 0 | 10 | | 71% |
| 41 | Lindsey | 0 | 0 |  |  | | 1 | |  | | 1 | | 1 | 0 | 1 | | 1 | | 1 |  |  |  | 1 | 0 | 1 | 1 | 0 | 9 | | 64% |
| 42 | Link 2003 | 0 | 0 |  |  | | 0 | |  | | 1 | | 1 | 0 | 1 | | 0.5 | | 1 |  |  |  | 1 | 0 | 1 | 1 | 0 | 7.5 | | 54% |
| 43 | Lo 2005 | 0 | 0 |  |  | | 1 | |  | | 1 | | 1 | 0 | 1 | | 1 | | 1 |  |  |  | 0 | 0 | 1 | 0 | 0 | 7 | | 50% |
| 44 | Lo 2009 | 0 | 0 |  |  | | 1 | |  | | 1 | | 1 | 0 | 1 | | 1 | | 1 |  |  |  | 1 | 0 | 1 | 1 | 1 | 10 | | 71% |
| 45 | Lo 2012 | 0 | 0 |  |  | | 1 | |  | | 1 | | 1 | 0 | 1 | | 1 | | 1 |  |  |  | 1 | 0 | 1 | 1 | 0 | 9 | | 64% |
| 46 | Lo 2006 | 0 | 0 |  |  | | 1 | |  | | 1 | | 1 | 0 | 1 | | 1 | | 1 |  |  |  | 1 | 0 | 1 | 1 | 1 | 10 | | 71% |
| 47 | Macfarlane 1993 | 0 | 0 |  |  | | 0 | |  | | 1 | | 1 | 0 | 1 | | 1 | | 1 |  |  |  | 1 | 0 | 1 | 1 | 0 | 8 | | 57% |
| 48 | Maksymowych 2014 | 0 | 0 |  |  | | 1 | |  | | 1 | | 1 | 0 | 1 | | 1 | | 1 |  |  |  | 1 | 0 | 1 | 1 | 0 | 9 | | 64% |
| 49 | McCauley 2001 | 0 | 0 |  |  | | 0 | |  | | 1 | | 1 | 0 | 1 | | 0 | | 1 |  |  |  | 0 | 0 | 0 | 0 | 0 | 4 | | 29% |
| 50 | McCrae 1992 | 0 | 0 |  |  | | 1 | |  | | 1 | | 1 | 0 | 1 | | 0 | | 1 |  |  |  | 0 | 0 | 1 | 1 | 0 | 7 | | 50% |
| 51 | Meredith 2009 | 0 | 1 |  |  | | 0 | |  | | 1 | | 1 | 0 | 1 | | 0 | | 1 |  |  |  | 0 | 0 | 1 | 1 | 0 | 7 | | 50% |
| 52 | Moisio 2009 | 0 | 0 |  |  | | 1 | |  | | 1 | | 1 | 0 | 1 | | 1 | | 1 |  |  |  | 1 | 0 | 0 | 1 | 1 | 9 | | 64% |
| 53 | Neumann 2007 | 0 | 0 |  |  | | 0 | |  | | 1 | | 1 | 0 | 1 | | 0 | | 1 |  |  |  | 0 | 0 | 1 | 1 | 0 | 6 | | 43% |
| 54 | Ochiai 2010 | 0 | 0 |  |  | | 0 | |  | | 0 | | 1 | 0 | 1 | | 1 | | 1 |  |  |  | 1 | 0 | 1 | 1 | 0 | 7 | | 50% |
| 55 | Okazaki 2014 | 0 | 0 |  |  | | 0 | |  | | 1 | | 1 | 0 | 1 | | 0 | | 1 |  |  |  | 1 | 0 | 1 | 1 | 0 | 7 | | 50% |
| 56 | Ratzlaff 2013 | 0 | 0 |  |  | | 1 | |  | | 1 | | 1 | 0 | 1 | | 1 | | 1 |  |  |  | 1 | 0 | 1 | 1 | 1 | 10 | | 71% |
| 57 | Ratzlaff 2014 | 0 | 0 |  |  | | 1 | |  | | 1 | | 1 | 0 | 1 | | 1 | | 1 |  |  |  | 1 | 0 | 1 | 1 | 0 | 9 | | 64% |
| 58 | Reichenbach 2008 | 0 | 0 |  |  | | 1 | |  | | 1 | | 1 | 0 | 1 | | 0 | | 1 |  |  |  | 0 | 0 | 1 | 0 | 0 | 6 | | 43% |
| No. | Cross-sectional Study | 1 | 2 | 3 | 4 | | 5 | | 6 | | 7 | | 8 | 9 | 10 | | 11 | | 12 | 13 | 14 | 15 | 16 | 17 | 18 | 19 | 20 | Total | | % |
| 59 | Reichenbach 2011 | 0 | 1 |  |  | | 1 | |  | | 1 | | 1 | 0 | 1 | | 0 | | 1 |  |  |  | 0 | 0 | 1 | 1 | 1 | 9 | | 64% |
| 60 | Roemer 2012 | 0 | 0 |  |  | | 1 | |  | | 1 | | 1 | 0 | 1 | | 0 | | 1 |  |  |  | 0 | 0 | 1 | 1 | 1 | 8 | | 57% |
| 61 | Scher 2008 | 0 | 0 |  |  | | 0 | |  | | 1 | | 1 | 0 | 1 | | 0 | | 1 |  |  |  | 0 | 0 | 1 | 1 | 0 | 6 | | 43% |
| 62 | Sengupta 2006 | 0 | 0 |  |  | | 1 | |  | | 1 | | 1 | 0 | 1 | | 1 | | 1 |  |  |  | 1 | 0 | 1 | 1 | 1 | 10 | | 71% |
| 63 | Sharma 2014 | 0 | 0 |  |  | | 1 | |  | | 1 | | 1 | 0 | 1 | | 1 | | 1 |  |  |  | 1 | 0 | 1 | 1 | 1 | 10 | | 71% |
| 64 | Sowers 2003 | 0 | 0 |  |  | | 1 | |  | | 1 | | 1 | 0 | 1 | | 0.5 | | 1 |  |  |  | 0 | 0 | 1 | 1 | 0 | 7.5 | | 54% |
| 65 | Stefanik 2014 | 0 | 0 |  |  | | 1 | |  | | 1 | | 1 | 0 | 1 | | 1.0 | | 1 |  |  |  | 1 | 0 | 1 | 1 | 1 | 10 | | 71% |
| 66 | Stefanik 2012 | 0 | 0 |  |  | | 1 | |  | | 1 | | 1 | 0 | 1 | | 0 | | 1 |  |  |  | 0 | 0 | 1 | 1 | 1 | 8 | | 57% |
| 67 | Stehling 2010 | 0 | 1 |  |  | | 1 | |  | | 1 | | 1 | 0 | 1 | | 1 | | 1 |  |  |  | 0 | 0 | 1 | 1 | 1 | 10 | | 71% |
| 68 | Torres 2006 | 1 | 0 |  |  | | 1 | |  | | 1 | | 1 | 0 | 1 | | 0.5 | | 1 |  |  |  | 0 | 1 | 1 | 1 | 0 | 9.5 | | 68% |
| 69 | Wang 2005 | 0 | 0 |  |  | | 0 | |  | | 1 | | 1 | 0 | 1 | | 1 | | 1 |  |  |  | 0 | 0 | 1 | 1 | 1 | 8 | | 57% |
| 70 | Zhai 2006 | 1 | 0 |  |  | | 1 | |  | | 1 | | 1 | 0 | 1 | | 1 | | 1 |  |  |  | 1 | 0 | 1 | 1 | 1 | 11 | | 79% |
|  |  |  |  |  |  | |  | |  | |  | |  |  |  | |  | |  |  |  |  |  |  |  | Mean | | 8.3 | | 59% |
|  |  |  |  |  |  | |  | |  | |  | |  |  |  | |  | |  |  |  |  |  |  |  | Max | | 14 | |  |
|  |  |  | |  |  |  | |  |  |  | |  |  |  |  |  | |  |  |  |  |  | | | | | | | | |
|  |  |  | |  |  |  | |  |  |  | |  |  |  |  |  | |  |  |  |  |  | | | | | | | | |

# Supplementary Tables 4: Quality Scoring Results cohort studies

|  |  | Quality Scoring Criteria | | | | | | | | | | | | | | | | | | | |  |  |
| --- | --- | --- | --- | --- | --- | --- | --- | --- | --- | --- | --- | --- | --- | --- | --- | --- | --- | --- | --- | --- | --- | --- | --- |
| No. | Cohort Study | 1 | 2 | 3 | 4 | 5 | 6 | 7 | 8 | 9 | 10 | 11 | 12 | 13 | 14 | 15 | 16 | 17 | 18 | 19 | 20 | total | % |
| 1 | Agricola 2013 | 0 | 1 |  | 1 | 1 |  | 1 | 1 | 0 | 1 | 1 | 1 | 0 | 1 | 0 | 0 | 0 | 1 | 1 | 1 | 12 | 67% |
| 2 | Agricola 2013 | 0 | 1 |  | 1 | 1 |  | 1 | 1 | 0 | 1 | 1 | 1 | 0 | 1 | 0 | 0 | 0 | 1 | 1 | 1 | 12 | 67% |
| 3 | Agricola 2013 | 0 | 1 |  | 1 | 1 |  | 1 | 1 | 0 | 1 | 1 | 1 | 0 | 1 | 0 | 1 | 0 | 1 | 1 | 1 | 13 | 72% |
| 4 | Bruyere 2003 | 0 | 0 |  | 0 | 0 |  | 1 | 1 | 0 | 1 | 0 | 1 | 0 | 0 | 0 | 1 | 0 | 1 | 1 | 1 | 8.0 | 44% |
| 5 | Carnes 2012 | 0 | 0 |  | 0 | 1 |  | 1 | 1 | 0 | 1 | 1 | 1 | 0 | 0 | 0 | 0 | 0 | 1 | 1 | 1 | 9.0 | 50% |
| 6 | Carrino 2006 | 0 | 0 |  | 0 | 0 |  | 0 | 1 | 0 | 1 | 0 | 1 | 0 | 0 | 0 | 0 | 0 | 1 | 0 | 0 | 4.0 | 22% |
| 7 | Cicuttini 2004 | 0 | 0 |  | 1 | 1 |  | 1 | 1 | 0 | 1 | 1 | 1 | 1 | 1 | 1 | 1 | 0 | 1 | 1 | 1 | 14 | 78% |
| 8 | Crema 2013 | 0 | 0 |  | 0 | 1 |  | 1 | 1 | 0 | 1 | 0 | 1 | 0 | 0 | 0 | 0 | 0 | 1 | 1 | 1 | 8.0 | 44% |
| 9 | Crema 2014 | 1 | 0 |  | 0 | 1 |  | 1 | 1 | 0 | 1 | 1 | 1 | 0 | 0 | 0 | 0 | 0 | 1 | 1 | 1 | 10.0 | 56% |
| 10 | Davies-Tuck 2008 | 0 | 0 |  | 0 | 1 |  | 1 | 1 | 0 | 1 | 1 | 1 | 0 | 0 | 1 | 0 | 0 | 1 | 1 | 1 | 10.0 | 56% |
| 11 | Davies-Tuck 2010 | 0 | 1 |  | 0 | 1 |  | 1 | 1 | 0 | 1 | 1 | 1 | 0 | 0 | 1 | 0 | 0 | 1 | 1 | 1 | 11 | 61% |
| 12 | De-Lange 2014 | 0 | 0 |  | 0 | 1 |  | 1 | 1 | 0 | 1 | 0 | 1 | 1 | 1 | 0 | 1 | 0 | 1 | 1 | 1 | 11 | 61% |
| 13 | Dieppe 1993 | 0 | 0 |  | 0 | 1 |  | 1 | 1 | 0 | 1 | 0 | 1 | 0 | 1 | 0 | 1 | 0 | 1 | 1 | 0 | 9.0 | 50% |
| 14 | Ding 2006 | 1 | 0 |  | 1 | 1 |  | 1 | 1 | 0 | 1 | 1 | 1 | 0 | 0 | 0 | 0 | 0 | 1 | 1 | 1 | 11 | 61% |
| 15 | Ding 2008 | 1 | 0 |  | 0 | 1 |  | 1 | 1 | 0 | 1 | 1 | 1 | 1 | 0 | 1 | 0 | 1 | 1 | 1 | 1 | 13 | 72% |
| 16 | Dore 2010 | 1 | 0 |  | 0 | 1 |  | 1 | 1 | 0 | 1 | 1 | 1 | 0 | 0.5 | 0 | 1 | 0 | 1 | 1 | 1 | 11.5 | 64% |
| 17 | Dore 2010 | 0 | 0 |  | 0 | 1 |  | 1 | 1 | 0 | 1 | 1 | 1 | 0 | 0 | 0 | 0 | 0 | 1 | 1 | 1 | 9.0 | 50% |
| 18 | Dore 2010 | 0 | 0 |  | 0 | 1 |  | 1 | 1 | 0 | 1 | 1 | 1 | 0 | 0 | 0 | 0 | 0 | 1 | 1 | 1 | 9.0 | 50% |
| 19 | Driban 2011 | 0 | 0 |  | 0 | 1 |  | 1 | 1 | 0 | 1 | 1 | 1 | 0 | 0 | 0 | 0 | 0 | 1 | 1 | 1 | 9.0 | 50% |
| 20 | Driban 2012 | 0 | 0 |  | 0 | 1 |  | 0 | 1 | 0 | 1 | 0 | 1 | 0 | 0 | 0 | 0 | 0 | 0 | 1 | 0 | 5.0 | 28% |
| 21 | Driban 2013 | 0 | 0 |  | 0 | 1 |  | 1 | 1 | 0 | 1 | 1 | 1 | 0 | 1 | 0 | 1 | 0 | 1 | 1 | 1 | 11.0 | 61% |
| 22 | Everhart 2014 | 0 | 0 |  | 0 | 1 |  | 1 | 1 | 0 | 1 | 1 | 1 | 0 | 1 | 0 | 1 | 0 | 1 | 1 | 1 | 11.0 | 61% |
| 23 | Felson 2003 | 0 | 0 |  | 1 | 1 |  | 1 | 1 | 1 | 1 | 1 | 1 | 1 | 0 | 1 | 1 | 1 | 1 | 1 | 1 | 15.0 | 83% |
| 24 | Foong 2014 | 0 | 0 |  | 0 | 1 |  | 1 | 1 | 0 | 1 | 1 | 1 | 0 | 1 | 1 | 1 | 0 | 1 | 1 | 1 | 12.0 | 67% |
| 25 | Guermazi 2014 | 0 | 0 |  | 0 | 1 |  | 1 | 1 | 0 | 1 | 0 | 1 | 0 | 1 | 0 | 1 | 0 | 1 | 1 | 1 | 10.0 | 56% |
| 26 | Haugen 2014 | 0 | 0 |  | 0 | 1 |  | 1 | 1 | 0 | 1 | 1 | 1 | 0 | 1 | 0 | 1 | 0 | 1 | 1 | 1 | 11.0 | 61% |
| 27 | Haugen 2014 | 0 | 0 |  | 0 | 1 |  | 1 | 1 | 0 | 1 | 1 | 1 | 0 | 1 | 0 | 1 | 0 | 1 | 1 | 1 | 11.0 | 61% |
| 28 | Hernandez-Molina 2008 | 0 | 0 |  | 0 | 1 |  | 0 | 1 | 0 | 1 | 1 | 1 | 0 | 0 | 0 | 0 | 0 | 1 | 1 | 1 | 8.0 | 44% |
| No. | Cohort Study | 1 | 2 | 3 | 4 | 5 | 6 | 7 | 8 | 9 | 10 | 11 | 12 | 13 | 14 | 15 | 16 | 17 | 18 | 19 | 20 | total | % |
| 29 | Hochberg 2014 | 0 | 0 |  | 1 | 0 |  | 1 | 1 | 0 | 1 | 1 | 1 | 0 | 1 | 0 | 1 | 0 | 1 | 1 | 1 | 11.0 | 61% |
| 30 | Hudelmaier 2013 | 0 | 0 |  | 0 | 1 |  | 1 | 1 | 0 | 1 | 1 | 1 | 0 | 0 | 0 | 1 | 0 | 1 | 1 | 0 | 9.0 | 50% |
| 31 | Hunter 2006 | 0 | 0 |  | 0 | 1 |  | 1 | 1 | 0 | 1 | 1 | 1 | 0 | 0 | 0 | 0 | 0 | 1 | 1 | 0 | 8.0 | 44% |
| 32 | Kornaat 2007 | 0 | 0 |  | 0 | 1 |  | 1 | 1 | 0 | 1 | 1 | 1 | 0 | 0 | 0 | 1 | 0 | 1 | 1 | 1 | 10.0 | 56% |
| 33 | Koster 2011 | 0 | 0 |  | 0 | 1 |  | 0 | 1 | 0 | 1 | 0 | 1 | 1 | 0 | 1 | 0 | 0 | 1 | 1 | 0 | 8 | 44% |
| 34 | Kothari 2010 | 1 | 0 |  | 0 | 1 |  | 1 | 1 | 0 | 1 | 0 | 1 | 0 | 0 | 0 | 0 | 0 | 1 | 1 | 1 | 9.0 | 50% |
| 35 | Kubota 2010 | 0 | 0 |  | 0 | 1 |  | 0 | 1 | 0 | 1 | 0 | 1 | 1 | 0 | 0 | 0 | 0 | 1 | 1 | 0 | 7.0 | 39% |
| 36 | Liu 2014 | 0 | 1 |  | 1 | 0 |  | 1 | 1 | 0 | 1 | 1 | 1 | 1 | 0 | 0 | 0 | 0 | 0 | 1 | 0 | 9.0 | 50% |
| 37 | Lo 2012 | 0 | 0 |  | 0 | 1 |  | 1 | 1 | 0 | 1 | 0 | 1 | 0 | 0 | 0 | 1 | 0 | 1 | 1 | 1 | 9.0 | 50% |
| 38 | Madan-Sharma 2008 | 0 | 0 |  | 0 | 1 |  | 1 | 1 | 0 | 1 | 0.5 | 1 | 0 | 0 | 0 | 0 | 0 | 1 | 1 | 1 | 8.5 | 47% |
| 39 | Mazzuca 2004 | 0 | 0 |  | 1 | 1 |  | 1 | 1 | 0 | 1 | 1 | 1 | 0 | 0 | 0 | 1 | 0 | 1 | 1 | 1 | 11 | 56% |
| 40 | Mazzuca 2005 | 0 | 0 |  | 1 | 1 |  | 1 | 1 | 0 | 1 | 1 | 1 | 0 | 0 | 0 | 1 | 0 | 1 | 1 | 0 | 10 | 56% |
| 41 | Moisio 2009 | 0 | 0 |  | 0 | 1 |  | 1 | 1 | 0 | 1 | 1 | 1 | 0 | 0 | 0 | 1 | 0 | 1 | 1 | 1 | 10.0 | 56% |
| 42 | Parsons 2014 | 0 | 0 |  | 0 | 1 |  | 1 | 1 | 0 | 1 | 0 | 1 | 0 | 0 | 0 | 1 | 0 | 1 | 1 | 1 | 9.0 | 50% |
| 43 | Pelletier 2007 | 0 | 0 |  | 0 | 1 |  | 1 | 1 | 0 | 1 | 1 | 1 | 0 | 0 | 0 | 0 | 0 | 1 | 1 | 1 | 9.0 | 50% |
| 44 | Raynauld 2008 | 0 | 0 |  | 0 | 1 |  | 1 | 1 | 0 | 1 | 1 | 1 | 0 | 0 | 0 | 0 | 0 | 1 | 1 | 1 | 9.0 | 50% |
| 45 | Raynauld 2011 | 0 | 0 |  | 0 | 1 |  | 1 | 1 | 0 | 1 | 1 | 1 | 0 | 1 | 0 | 1 | 0 | 1 | 1 | 1 | 11.0 | 61% |
| 46 | Raynauld 2013 | 0 | 0 |  | 0 | 1 |  | 1 | 1 | 0 | 1 | 1 | 1 | 0 | 1 | 0 | 1 | 0 | 1 | 1 | 1 | 11.0 | 61% |
| 47 | Roemer 2009 | 0 | 0 |  | 0 | 1 |  | 1 | 1 | 0 | 1 | 1 | 1 | 0 | 0 | 0 | 0 | 0 | 1 | 1 | 1 | 9.0 | 50% |
| 48 | Roemer 2009 | 0 | 0 |  | 0 | 1 |  | 1 | 1 | 0 | 1 | 0 | 1 | 0 | 0 | 0 | 0 | 0 | 1 | 1 | 1 | 8.0 | 44% |
| 49 | Roemer 2012 | 0 | 0 |  | 0 | 1 |  | 1 | 1 | 0 | 1 | 0 | 1 | 0 | 0 | 0 | 0 | 0 | 1 | 1 | 1 | 8.0 | 44% |
| 50 | Scher 2008 | 0 | 1 |  | 0 | 1 |  | 1 | 1 | 0 | 1 | 1 | 1 | 0 | 0 | 0 | 1 | 0 | 1 | 1 | 0 | 10 | 56% |
| 51 | Sowers 2011 | 0 | 0 |  | 0 | 1 |  | 1 | 1 | 0 | 1 | 0.5 | 1 | 0 | 1 | 0 | 1 | 0 | 1 | 1 | 0 | 9.5 | 53% |
| 52 | Tanamas 2010 | 0 | 0 |  | 0 | 1 |  | 1 | 1 | 0 | 1 | 1 | 1 | 0 | 0 | 1 | 0 | 0 | 1 | 1 | 0 | 9.0 | 50% |
| 53 | Tanamas 2010 | 0 | 0 |  | 0 | 1 |  | 1 | 1 | 0 | 1 | 1 | 1 | 0 | 0 | 0 | 0 | 0 | 1 | 1 | 0.5 | 8.5 | 47% |
| 54 | Wildi 2010 | 0 | 0 |  | 0 | 1 |  | 1 | 1 | 0 | 1 | 1 | 1 | 0 | 0 | 0 | 0 | 0 | 1 | 1 | 1 | 9.0 | 50% |
| 55 | Zhang 2011 | 0 | 0 |  | 0 | 1 |  | 1 | 1 | 0 | 1 | 1 | 1 | 0 | 0 | 0 | 1 | 0 | 1 | 1 | 0 | 9.0 | 50% |
|  |  |  |  |  |  |  |  |  |  |  |  |  |  |  |  |  |  |  |  |  | mean | 9.7 | 54% |
|  |  |  |  |  |  |  |  |  |  |  |  |  |  |  |  |  |  |  |  |  | Max | 18.0 |  |

# Supplementary Tables 5: Quality Scoring Results case-control studies

|  |  | Quality Scoring Criteria | | | | | | | | | | | | | | | | | | | |  |  |
| --- | --- | --- | --- | --- | --- | --- | --- | --- | --- | --- | --- | --- | --- | --- | --- | --- | --- | --- | --- | --- | --- | --- | --- |
| No. | Case control study | 1 | 2 | 3 | 4 | 5 | 6 | 7 | 8 | 9 | 10 | 11 | 12 | 13 | 14 | 15 | 16 | 17 | 18 | 19 | 20 | total | % |
| 1 | Aitken 2013 | 0 | 0 | 0 |  | 0 | 0 | 1 | 1 | 0 | 1 | 0 | 1 |  | 1 |  | 0 | 0 | 1 | 1 | 1 | 8 | 47% |
| 2 | Barr 2012 | 0 | 1 | 1 |  | 1 | 0 | 1 | 1 | 0 | 1 | 1 | 1 |  | 1 |  | 1 | 0 | 1 | 1 | 1 | 13 | 76% |
| 3 | Bennell 2008 | 0 | 0 | 0 |  | 1 | 0 | 1 | 1 | 0 | 1 | 1 | 1 |  | 0 |  | 1 | 0 | 1 | 1 | 1 | 10 | 59% |
| 4 | Bowes 2013 | 0 | 1 | 1 |  | 1 | 0 | 1 | 1 | 0 | 1 | 0 | 1 |  | 1 |  | 1 | 0 | 1 | 1 | 1 | 12 | 71% |
| 5 | Doherty 2008 | 0 | 0 | 0 |  | 1 | 0 | 1 | 1 | 0 | 1 | 1 | 1 |  | 0 |  | 0 | 0 | 1 | 1 | 1 | 9 | 53% |
| 6 | Felson 2007 | 0 | 1 | 1 |  | 1 | 0 | 1 | 1 | 0 | 1 | 1 | 1 |  | 0 |  | 1 | 0 | 1 | 1 | 1 | 12 | 71% |
| 7 | Hunter 2013 | 0 | 0 | 1 |  | 1 | 0 | 1 | 1 | 0 | 1 | 0 | 1 |  | 1 |  | 1 | 0 | 1 | 1 | 0 | 10 | 59% |
| 8 | Javaid 2012 | 0 | 0 | 1 |  | 1 | 1 | 1 | 1 | 0 | 1 | 1 | 1 |  | 0 |  | 0 | 0 | 1 | 1 | 0 | 10 | 59% |
| 9 | Javaid 2010 | 0 | 1 | 1 |  | 1 | 1 | 1 | 1 | 0 | 1 | 1 | 1 |  | 0 |  | 1 | 0 | 1 | 1 | 1 | 13 | 76% |
| 10 | Neogi 2013 | 0 | 1 | 1 |  | 1 | 0 | 1 | 1 | 0 | 1 | 0 | 1 |  | 0 |  | 1 | 0 | 1 | 1 | 1 | 11 | 65% |
| 11 | Neogi 2009 | 0 | 0 | 1 |  | 1 | 1 | 1 | 1 | 0 | 1 | 0 | 1 |  | 0 |  | 0 | 0 | 1 | 1 | 1 | 10 | 59% |
| 12 | Nicholls 2011 | 0 | 0 | 1 |  | 1 | 1 | 1 | 1 | 0 | 1 | 1 | 1 |  | 1 |  | 1 | 0 | 0 | 1 | 1 | 12 | 71% |
| 13 | Ratzlaff 20148 | 0 | 1 | 1 |  | 0 | 0 | 1 | 1 | 0 | 1 | 1 | 1 |  | 0 |  | 1 | 0 | 1 | 1 | 1 | 11 | 65% |
| 14 | Stahl 2011 | 0 | 0 | 1 |  | 1 | 0 | 1 | 1 | 0 | 1 | 1 | 1 |  | 0 |  | 0 | 0 | 1 | 0 | 0 | 8 | 47% |
| 15 | Wluka 2005 | 0 | 0 | 0 |  | 1 | 0 | 1 | 1 | 0 | 1 | 1 | 1 |  | 0 |  | 0 | 0 | 0 | 1 | 1 | 8 | 47% |
| 16 | Zhao 2010 | 1 | 0 | 0 |  | 1 | 0 | 1 | 1 | 0 | 1 | 0.5 | 1 |  | 0 |  | 0 | 1 | 1 | 1 | 0 | 9.5 | 56% |
|  |  |  |  |  |  |  |  |  |  |  |  |  |  |  |  |  |  |  |  |  | mean | 10.1 | 59% |
|  |  |  |  |  |  |  |  |  |  |  |  |  |  |  |  |  |  |  |  |  | max | 17 |  |

# Supplementary Tables 6: - A description of the included studies, the relationships examined and the quality of each paper

| Author of  longitudinal studies | Patient  number  (n) | Study demographics | Subchondral bone feature assessed | Structural progression or severity / pain measure | Statistical analysis | Quality (score %) |
| --- | --- | --- | --- | --- | --- | --- |
| Knee cohort studies | | | | | | |
| Bruyere 2003 | 56 | Knee OA (ACR criteria), gender distribution unknown, mean age 65 yrs | Subchondral tibial bone BMD (DXA) (C) | Minimum medial JSW TFJ after one year (L) | Multiple regression | Low (44) |
| Carnes 2012 | 395 | Randomly selected older adults with over 52% knee ROA. 50% female, mean age 63 yrs. TASOAC | MRI tibial Bone area (C) | Semi-quantitative cartilage defect progression TFJ (L) | Logistic regression | Low (50) |
| Carrino 2006 | 32 | Chronic knee pain with MRI features of OA. 63% female, mean age 51 yrs. USA | Crude presence of MRI BML, bone cyst TFJ (C) and (L) | Graded cartilage defect TFJ (L) | Crude comparison | Low (22) |
| Cicuttini 2004 | 113 | Symptomatic, clinical (ACR) knee OA with mild to moderate TFJ ROA, mean age 64yrs, mean BMI 29, 58% females. Australia | Baseline Quantitative MRI tibial bone area (C) | TKR incidence (L) over 4 years | Logistic regression | High (78) |
| Crema 2013 | 1351 | Knee OA or at high risk of it. 39% ROA, 62% female, mean age 62 yrs. MOST | MRI Incident BML (WORMS)  TFJ  (L) | Progressive (30 month) semi-quantitative cartilage defect  (WORMS) TFJ (L) | Logistic regression | Low (44) |
| Crema 2014 | 163 | Clinical knee OA, 37% knee ROA, 54% female, Mean age 58 yrs. | MRI BML (semi-quantitative)  (C) (all regions) | Cartilage loss (semi-quantitative)  (L)  (all regions) | Logistic regression | High (56) |
| Davies-Tuck 2008 | 117 | ACR knee OA. 58% female, mean age 64 yrs. Australia | Baseline MRI tibial bone plateau area (C) TFJ | Progressive semi-quantitative cartilage defect score (L) medial and lateral TFJ | Linear regression | High (56) |
| Davies-Tuck 2010 | 271 | No clinical knee OA (ACR clinical criteria) and no current or historic knee pathology, mean age 58yrs, 65% female, mean BMI 25. Melbourne. | Incident BML (new BML after 2 years with no BMLs at baseline) MRI TFJ (L) | Progression in semi-quantitative MRI cartilage defects score after 2 years. TFJ (L) | Logistic regression | High (61) |
| De-Lange 2014 abstract | 133 | Symptomatic OA knee. ROA Knee (>50%), 80% female, mean age 60 yrs. GARP study | MRI osteophytes (medial or lateral TFJ) (C) | Radiographic JSN progression (OARSI) (L) | Generalised estimation equation models | High (61) |
| Dieppe 1993 | 94 | Symptomatic and ROA knee (100%). 96% women, mean age 64 yrs, mean BMI 26. Referrals to hospital rheumatology unit | Baseline late and or early-phase subchondral bone scintigraphy signal (C) | Progression of JSN by ≥2mm or knee operation after 5 years (L) | Pearson Chi squared test | Low (50) |
| Ding 2006 | 325 | Mostly no ROA knee (17% ~ KL =1), 58% female, Mean age 45 yrs. Mean BMI 27. Offspring study | Baseline MRI tibial bone area (C) TFJ | Change in semi-quantitative MRI cartilage defect scores over 2.3 yrs (L) TFJ | Logistic regression | High (61) |
| Ding 2008 | 252 | Randomly selected adults with 15% knee ROA. 58% female, mean age 45 yrs. | Baseline MRI  tibial bone area (C) TFJ | Progressive Cartilage volume loss (L) TFJ | Multivariable linear regression | High (72) |
| Dore 2010 | 395 | Symptomatic knee OA, knee ROA (58%). 51% female, mean age 63 yrs, mean BMI 28. TASOAC | MRI BML size (L) regional or whole TFJ over 2.7 years | Change in WOMAC pain (L) over 2.7 years  Incident TKR over 5 years (L) | Mixed effects models | High (64) |
| Dore 2010  12(6) | 405 | Prevalent knee OA, 50% female, Mean age 63 yrs. TASOAC | Baseline semi-quantitative BML severity (C) TFJ | Ipsi-compartmental cartilage volume loss (L) | Logistic regression and generalised estimating equations | Low (50) |
| Dore 2010 | 341 | Older adult cohort. Right knees only. Mean age 63 yrs, ~48% female, mean BMI 27.  TASOAC | Baseline proximal tibial BMD, DXA  Baseline tibial bone area MRI (C) | Increase or no increase in semi-quantitative MRI cartilage defects over 2.7 years (L) | Logistic regression | Low (50) |
| Driban 2011 | 44 | ACR knee OA(100%). 100% knee ROA, , 52% female, Mean age 65 yrs. Clinical trial of Vitamin D | Baseline 3D BML volume (C) and 24 month change in 3D BML volume (L) in TFJ compartments | 24 month change in ipsicompartmental full thickness cartilage lesion area (L) | Multiple linear regression,  Spearman correlations | Low (50) |
| Driban 2012  Abstract | 38 | Knee ROA (100%). 66% female, mean age 61 yrs. OAI | MRI BML volume change (L) TFJ over 24 months | Change in cartilage thickness and denuded area of bone (L) TFJ over 24 months | Pearson correlation  coefficients | Low (28) |
| Driban 2013 | 404 | Prevalent ROA knee (71%) 49% female, mean age 63 yrs. OAI | Knee baseline BML volume (C)  BML volume 48 month change (L)  (TFJ) | 48 month change in WOMAC pain (L) and  OARSI JSN grade (L)  (TFJ) | Multiple linear regressions & logistic regression | High (61) |
| Everhart 2014 | 1338 | Prevalent ROA knee (74%) 60% female, Mean age 62 yrs. OAI | Baseline TFJ subchondral surface ratio of medial and lateral TFJ compartments (C) | Incident frequent knee pain at 48 months or radiographic progression of lateral or medial knee TFJ OA at 48 months (L) | Logistic regression | High (61) |
| Felson 2003 | 223 | ACR knee OA and 75% ROA. 42% female, mean age 66 yrs. BOKS | Baseline presence of BML in medial or lateral TFJ (C) | OARSI JSN grade progression of TFJ (L) | Generalised estimating  equations | High (83) |
| Foong 2014 | 198 | 17% ROA knee, 42% female, Mean age 47 yrs. Offspring study | Change in quantitative BML size (L) and  incident BMLs (L)  In all three knee compartments over 8 years | WOMAC Knee pain severity over 8 years (L) | Linear regression | High (67) |
| Guermazi 2014  Abstract | 196 | Knee ROA (24%), 62% female, mean age 60 yrs | Semi-quantitative BML score  WORMS (C)  TFJ | Cartilage thickness loss over 30 months (L) | multivariable logistic regression, | High (56) |
| Hernandez-Molina 2008 | 258 | ACR knee OA and 77% ROA. 43% female, mean age 67 yrs. BOKS | Crude presence of central BMLs on MRI (C) TFJ | Semi-quantitative cartilage defect (WORMS) (L) TFJ | Logistic regression | Low (44) |
| Hochberg 2014  Abstract | 1024 | 100% Symptomatic and radiographic knee OA (the ‘progressor’ arm). OAI | Semi-quantitative MRI baseline femoral condyle BML size (C) | Incident TKR over 6 years (L) | Multivariable Cox proportional hazard models | High (61) |
| Hudelmaier 2013 abstract | 899 | Prevalent ROA knee, ROA 60%, 60% women, mean age 62 yrs, OAI | Annual change in Segmented MRI knee bone area (L) | Baseline KL grade (C) | Non-paired t-test | Low (50) |
| Hunter 2006 | 217 | ACR knee OA. 44% female, mean age 66 yrs. BOKS | Change in MRI semi-quantitative BML score (L) TFJ | Change in semi-quantitative cartilage defect score (WORMS) (L) TFJ | Generalised estimating  equations | Low (44) |
| Kornaat 2007 | 182 | OA knee symptoms (38%) and ROA (38%). 80% female, mean age 59 yrs. GARP | Semi-quantitative MRI BML change over 2 years (L) TFJ | Mean WOMAC pain over 2 years | Linear mixed models | High (56) |
| Koster 2011 | 117 | One year follow up after acute knee trauma in primary care, 12% ROA knee, mean age 41yrs, 43% female, mean BMI 26, HONEUR | Baseline BML presence (C) TFJ | Any progression in KL grade over 1 year (L) TFJ | Logistic regression | Low (44) |
| Kothari 2010 | 177 | Some WOMAC dysfunction and >74% ROA knee, 79% female, mean age 66 yrs. MAK-2 | Semi-quantitative baseline MRI BML, bone cyst and attrition  (WORMS) (C) TFJ | Semi-quantitative cartilage defect score change over 2 years (WORMS) (L) TFJ. | Logistic regression, with generalised  estimating equations | Low (50) |
| Kubota 2010 | 122 | Clinical and ROA (80%) of the knee. >90% female, mean age 68 yrs. Japan | MRI BML semi-quantitative volume score change over 6 months (L) TFJ | KL grade progression over 6 months (L) TFJ | Mann-Whitney U-test | Low (39) |
| Lo 2012 | 497 | 52% ROA knee, 47% female , Mean age 64 yrs. OAI | DXA measured  medial:lateral periarticular BMD and MRI BVF, trabecular number, thickness and spacing (C) | OARSI medial TFJ JSN grade progression between 24 and 48 months (L) | Logistic regression | Low (50) |
| Liu 2014 S470  Abstract | 128 | Medial knee OA, KL grade 4. Japan | Baseline Semi-quantitative osteophyte score (WORMS) (C) TFJ | Incident TKR at 6 months follow up (L) | Mann Whitney-U test & ROC curve | Low (50) |
| Madan-Sharma 2008 | 186 | Prevalent ROA knee (40%). 81% female, mean age 60 yrs. GARP | Baseline MRI semi-quantitative BML, bone cyst (C) TFJ | OARSI medial TFJ JSN grade progression over 2 years (L) TFJ | Logistic regression | Low (47) |
| Mazzuca 2004 | 86 | 100% female, mean age 55 yrs, mean BMI 37. | Baseline late-phase bone scintigraphy (adjusted for normal bone uptake) of the medial tibia and whole knee (C) | Progression of minimum JSN of the medial TFJ from baseline to 30 months  (L) | Pearson  correlation coefficients | High (56) |
| Mazzuca 2005 | 174 | 100% female, mean age 56 yrs, mean BMI 36. A placebo controlled trial of doxycycline | Baseline late-phase bone scintigraphy (adjusted for normal bone uptake) of the medial tibia and whole knee (C) | Progression of minimum JSN of the medial TFJ from baseline to 30 months  (L) | multiple linear regression | High (56) |
| Moisio 2009 | 168 | Some WOMAC dysfunction and 90% ROA knee. 78% female, mean age 66 yrs. MAK-2 | Baseline MRI semi-quantitative BML score (C) TFJ and PFJ | Incident frequent knee pain 2 years after baseline (L) | Logistic regression | High (56) |
| Parsons 2014  Abstract | 559 | Knee ROA (100%), 73% female, mean age 63 yrs. SEKOIA | Baseline semi-quantitative BML score (C) | Annual TFJ JSN (L) | Linear regression | Low (50) |
| Pelletier 2007 | 107 | 100% radiographic OA knee, 64% female, Mean age 62 yrs. Bisphosphonate Trial | Regional semi-quantitative baseline BML score (medial or lateral TFJ) (C) | Regional cartilage volume over 24 months (medial or lateral TFJ) (L) | Multivariate regression | Low (50) |
| Raynauld 2008 | 86 | 64% female, Mean age 61 yrs. Trial of bisphosphonate | Change in BML size (L) at 24 months in TFJ | Medial cartilage volume loss (L) at 24 months in TFJ | Multivariate linear regression | Low (50) |
| Raynauld 2011 | 123 | Symptomatic knee OA of the medial TFJ, 65% female, mean age 61 yrs, mean BMI 32. | Baseline semi-quantitative BML score (C) TFJ | Incidence of TKR over 3 years (L) | Logistic regression | High (61) |
| Raynauld 2013 | 57 | Patients from a chondroitin trial  81% female, mean age 63, mean BMI 31. | Baseline semi-quantitative BML WORMS score (C)  Medial TFJ | Incident TKR (L) 4 year follow up  Time to TKR | Logistic regression, Cox regression | High (61) |
| Roemer 2009 | 395 | Knee OA or at high risk of it. 33% ROA. 68% female, mean age 63 yrs. MOST | Change in MRI semi-quantitative BML size over 30 months (WORMS) (L) TFJ and PFJ | Progression in semi-quantitative cartilage defects in (WORMS) over 30 months (L) TFJ and PFJ | Logistic regression | Low (50) |
| Roemer 2009 | 347 | Knee OA or at high risk of it. 14% ROA. 65% female, mean age 64 yrs. MOST | Baseline MRI BML crude presence or absence (WORMS) (L) TFJ | Semi-quantitative cartilage defect progression over 30 months (WORMS) (L) TFJ | Logistic regression | Low (44) |
| Roemer 2012 | 177 | Chronic knee pain, 71% knee ROA, 49% female. Mean age 52 yrs. Glucosamine trial | Semi-quantitative BML  (WORMS) TFJ and PFJ (C) | Semi-quantitative cartilage score 6-month progression  TFJ and PFJ (L) | Logistic regression | Low (44) |
| Sowers 2011 | 363 | Minor OA symptoms with18% ROA knee. 100% female, median age 45 yrs. SWAN | Semi-quantitative MRI BML, osteophyte, bone cyst size  in TFJ (C) | Progression in KL grade  and WOMAC pain score  (11 years follow up ) (L) | Chi-square tests or Fisher exact  tests. Logistic regression | Low (53) |
| Tanamas 2010 | 109 | ACR knee OA, 73% ROA. 40% female, mean age 63 yrs. Australia | Semi-quantitative change in MRI Bone cyst or BML size (L) | Knee cartilage volume loss over 2 years (L) TFJ  Incident TKR over 4 years | Logistic regression | Low (47) |
| Tanamas 2010 | 109 | ACR knee OA, 72% ROA. 50% female, mean age 63 yrs, mean BMI 29. Australia | Baseline semi-quantitative MRI BML size (C) | Cartilage volume change over 2 years (L) TFJ  Annual change in WOMAC pain(L)  Incident TKR over 4 years | Logistic regression | Low (50) |
| Wildi 2010 | 161 | Symptomatic medial TFJ OA knee (ACR criteria). 100% ROA knee, 66% female, Licofelone trial | 24 month change in regional TFJ BML score WORMS (L) | 24 months change in WOMAC pain (L) or regional change in cartilage volume (L) | Multivariate regression | Low (50) |
| Zhang 2011 | 570 | Patients with knee OA or at high risk of it. 41% ROA knee. 68% female, mean age 62 yrs. MOST | Semi-quantitative change in MRI BML size (L) TFJ over 30 months | Incidence of frequent knee pain, and categorical severity (L) over 30 months | Logistic regression | Low (50) |
| Knee case-controls studies | | | | | | |
| Aitken 2013 Abstract | 220 | 57% female, Mean age 45 yrs. Prevalence of ROA unknown. Offspring | Semi-quantitative BMLs tibia, femur & patella (C) | Cartilage volume and defect score Tibia and femur (L) | Linear regression | Low (47) |
| Bowes 2013 | 2197 | Cases of clinical knee OA (100% ROA, n=1312), control healthy knees (0% ROA, n=885). 56% female, mean age 61 yrs. OAI dataset | Change in segmented MRI 3D Bone area over 4 year (L) | KL grade defined ROA knee (C) | ANCOVA model | High (71) |
| Bennell 2008 | 116 | Cases of ACR medial knee OA (100% medial TFJ ROA) (n=75), asymptomatic control knees (n=41). 54% female, mean age 64 yrs. Australia | Volumetric BMD of tibial subchondral trabecular bone  (qCT) (C) | KL grade (C) | Generalised linear models | Low (59) |
| Felson 2007 | 330 | Patients at high risk of knee OA , cases with incident pain (n=110, ROA 30%), controls without pain (n=220,ROA 20%); 66%female, mean age 63 yrs. MOST | Semi-quantitative MRI BML size increase (WORMS) (L) TFJ & PFJ | Incident frequent pain at 15 months (L) | Multiple logistic  regression, | High (71) |
| Hunter 2013  Abstract | 636 | 636 knees at risk of OA (ROA=0% at baseline). Cases of incident ROA n=318, Controls without incident ROA n=318, 67% female. Mean age 60 yrs. OAI | MRI bone area 8 knee regions (L) | Incident ROA knee  (KL grade ≥2)  (TFJ) (L) | Discrete-time Cox Proportional Hazards Regression | Low (59) |
| Javaid 2012 | 636 | Patients with prevalent ROA. Cases have painful knees (n= 546), controls have no knee pain (n=90); 65% female, mean age 73 yrs. Health aging and body composition study | Baseline Semi-quantitative MRI BML, bone cyst and attrition size (WORMS) (C) TFJ & PFJ | Presence of frequent knee pain (C) after 2 years | Conditional and marginal logistic regression | Low (59) |
| Javaid 2010 | 155 | Clinical knee OA, cases with incident pain (n=33), controls without pain (n=122); 67% female, mean age 59 yrs. ROA 0%; MOST | Baseline semi-quantitative MRI BML, osteophyte, bone cyst size (WORMS) (C) TFJ & PFJ | Incident frequent knee pain after 15 months (L) | Logistic regression | High (76) |
| Neogi 2013 | 531 | Incident knee TFJ ROA  cases (n=178), controls did not develop ROA (n=353); 62% female, mean age 61 yrs. OAI dataset | MRI 3D bone shape  (Tibia, Femur and patella) (C) | Incident TFJ ROA KL grade ≥2 (L) | Conditional Logistic regression | High (65) |
| Neogi 2009 | 4446 | Clinical knee OA. Within-knee subregion cases (n=973) had cartilage loss, controls (n=3473) had not. 64% female, mean age 63 yrs; ROA 59% MOST | Baseline semi-quantitative MRI Bone attrition size (WORMS) (C) TFJ | Cartilage defects progression (WORMS) after 30 months TFJ | Logistic regression | Low (59) |
| Ratzlaff 2014 | 278 | 59% female, mean age 64, mean BMI 30. 138 cases of TKR and 138 ROA matched controls. OAI | Total tibial BML volume 12 and 24 months before TKR and interval change between 12 and 24 (C) and (L) | Incident TKR (L) | Conditional logistic regression | High (65) |
| Scher 2008 | 65 | Patients with OA knee based upon radiography (>50% ROA), 54% female, mean age 51 yrs. USA | Presence of any baseline semi-quantitative MRI BMLs (C) | Incident TKR (L) over 3 years | Generalised estimating  equations | High (56) |
| Stahl 2011 | 60 | Clinical and 100% ROA knee cases (n=30) ; controls – healthy knees ROA 0% (n=30); All female, mean age 58 yrs. USA | Semi-quantitative MRI BML size (WORMS) (L) TFJ | Semi-quantitative Cartilage defect size (L) TFJ  WOMAC score | Generalised estimating  equations | Low (47) |
| Wluka 2005 | 149 | ACR knee OA cases (n=68), controls (n=81) without OA; 54% female, mean age 64 yrs. Australia | Change in MRI Tibial bone area (L) | Baseline radiographic JSN (C) | Logistic regression | Low (47) |
| Zhao 2010 | 38 | Clinical and ROA cases (n=24), control (n=14) knees (KL=0); 54% female, mean age 52 yrs | MRI BML volume (C) TFJ | Overlying cartilage defect progression after one year (WORMS) (L) TFJ  WOMAC pain | Student’s t test | Low (56) |

|  | Author of  Cross sectional studies | Patient  number  (n) | Study demographics | Subchondral bone feature assessed (method) region | Structural progression / pain outcome (method)  region | Statistical analysis | Quality (score%) | |
| --- | --- | --- | --- | --- | --- | --- | --- | --- |
| Knee cross-sectional studies | | | | | | | |  |
|  | Ai 2010 | 28 | Clinical knee OA. 55% ROA, 57% female, mean age 61 yrs. China | Semi-quantitative MRI BML and osteophytes (C) | Pain verbal rating scale (Likert) (C) | Fisher exact test | Low (57) | |
|  | Akamatsu 2014 Abstract | 192 | Varus ROA Knee 94%, 100% female, mean age 70 yrs. Japan | BMD (DXA) (C) (medial tibia & femoral condyle) | Medial TFJ JSN radiographic  (C) | Pearson’s Correlation | Low (57) | |
|  | Baranyay 2007 | 297 | No clinical knee OA (ACR clinical criteria) and no current or historic knee pathology, mean age 58yrs, 63% female, mean BMI 25. Melbourne | MRI BML defined as large or not large / absent in the medial and lateral compartments of TFJ  (C) | MRI semi-quantitative cartilage defects of medial and lateral compartments of TFJ  Quantitative Cartilage volume medial and lateral TFJ  (C) | Logistic regression | High (71%) | |
|  | Bilgici 2010 | 34 | ACR knee OA. ROA 65%, 71% female, mean age 50 yrs. Turkey | MRI BML (WORMS) (C) | WOMAC pain & pain VAS (C) | Linear regression | Low (57) | |
|  | Burnett 2012 | 42 | Knees with OA awaiting total knee replacement, 100% ROA, 60% Female, mean age 64 yrs. Canada | BMD of patellar facets (qCT) (C) | WOMAC pain – knee pain at rest (C) | Independent t-test | Low (57) | |
|  | Chiba 2012 | 60 | Prevalent Knee OA. ROA 50%. 100% female, mean age 68 yrs Japan | MRI Bone volume fraction & trabecular thickness of the medial & lateral femur & tibia. (C) | Metric JSW (radiographic) (C) of the medial and lateral TFJ | Pearson’s Correlation | Low (57) | |
|  | Crema 2010 | 1283 | Knee OA or at high risk of OA. ROA knee 44%. 60% female, mean age 62 yrs. | MRI Bone cysts (WORMS) (C) | Cartilage defect  (WORMS) (C) | Comparison of tabulated data | Low (50) | |
|  | Ding 2005 | 372 | Mostly no ROA knee (17% ~ KL =1), 58% female, Mean age 45 yrs. Mean BMI 27. Offspring study | MRI quantitative tibial bone area (C) | Semi-quantitative MRI knee cartilage defect severity scores (C) TFJ | Linear regression | High (64) | |
|  | Dore 2009 | 740 | >15% ROA knee, 52% female, Mean age 62 yrs. TASOAC | DXA Tibial subchondral BMD (C) | Radiograph JSN grade and MRI cartilage defect and volume (C) | Multivariable analysis | High (71) | |
|  | Driban 2011 Abstract | 421 | NR | MRI Bone volume fraction (C) | Radiographic JSN (C) | Multiple linear regression | High (64) | |
|  | Driban 2011 Abstract | 285 | OAI Progression Cohort. No other demographic data available. | MRI bone volume fraction, trabecular number, spacing & thickness of medial tibia (C) | The presence of any grade of radiographic medial & lateral JSN (C) | Multiple linear regression | High (71) | |
|  | Eckstein 2010 | 73 | ROA knee (100%), 63% female with mean age of 61 yrs. OAI | MRI Tibial bone area (segmented) (C) | OARSI JSN grade (C) | Paired t-tests | Low (43) | |
|  | Felson 2001 | 401 | Prevalent knee OA (ACR criteria) and assumed ROA knee of 100%. 41% female, and mean age of 67 yrs. BOKS | Semi-quantitative MRI BMLs (C) | KL grade  chronic knee pain presence (C) | Chi-square or fisher exact test & logistic regression | Low (54) | |
|  | Fernandez-Madrid 1994 | 90 | ACR knee OA, 66% knee ROA, 65% female, mean age 55 yrs. USA | Crude presence of MRI BMLs, osteophytes (C) | KL grade and crude pain presence (C) | Chi-square | Low (46) | |
|  | Frobell 2010 | 891 | Three groups; pre-radiographic OA (KL grade <2), ROA and controls without OA, (total ROA knee 89%), 60% female, mean age 61 yrs. OAI | MRI Bone area – manual segmentation (C) | KL grade, OARSI JSN grade (C) | T tests and multivariate analyses | Low (57) | |
|  | Gudbergsen  2013 | 192 | Obesity and knee OA (ACR criteria). 81% female, mean age 63 yrs. Denmark | Semi-quantitative MRI BML (BLOKS) (C) | KL grade (C) | Spearman correlation analyses | Low (57) | |
|  | Guymer 2007 | 176 | No clinical knee OA (ACR clinical criteria) and no current or historic knee pathology, mean age 52 yrs, 100% female, mean BMI 27. Melbourne. | Presence or absence of MRI BMLs  (C) TFJ | Presence or absence of semi-quantitative cartilage defects  (C) TFJ | Logistic regression | High (71) | |
|  | Haverkamp 2011 | 609 | 1201 knees with 6% knee ROA and 25% knee pain prevalence, 100% female, and mean age 54 yrs, mean BMI 27. Rotterdam study | 2D bone shape knee  1. femur & tibial width  2. elevation of lateral tibial plateau  (C) | 1.Presence of diffuse cartilage defects semi-quantitative scoring (MRI).  2.Presence of ROA knee (KL≥2)  3.Pain severity VAS  (C) | Logistic and linear generalised estimating equation regression models | Low (46) | |
|  | Hayashi 2012 | 40 | ROA knee 57% with and without pain, 75% female, mean age 57 yrs. USA | Crude presence of MRI osteophytes, bone cysts (C) | Presence of pain on WOMAC pain subscale (C) | Logistic regression | Low (57) | |
|  | Hayes 2005 | 232 | Clinical and ROA knee or healthy patients. 36% ROA knee, 100% female, mean age 46 yrs. Southeast Michigan Cohort. | Semi-quantitative MRI BML, osteophyte, bone cyst (C) | KL grade and chronic pain presence (C) | Fisher Exact Test,  Chi-squared test | High (61) | |
|  | Hernandez-Molina 2008 | 1627 | Patients >50 yrs of age with and without knee pain. ROA knee 22%, 59% female, mean age 64 yrs. Framingham OA cohort | Semi-quantitative MRI bone attrition (WORMS) (C) | Pain severity and nocturnal pain (WOMAC) (C) | Logistic regression | High (71) | |
|  | Ip 2011 | 255 | Knee pain. ROA 38%, 56% female, median age 62 yrs. Canada | Semi-quantitative MRI BML (C) | WOMAC pain,  KL grade (C) | Logistic regression  & Pearson chi-squared | High (68) | |
|  | Jones 2004 | 372 | Mean age 45yrs, right knees, early ROA knee (3-14%), 58% female, mean BMI 27. Offspring | Tibial bone area (MRI) (C) | Radiographic JSN (C) | Linear Regression | Low (50) | |
|  | Kalichman 2007 | 213 | Predominantly knee OA (ACR criteria). ROA knee 75%, 41% female, mean age 67 yrs. BOKS | MRI patellar length ratio, trochlea sulcus angle (C) | JSN grade (C) | Logistic regression | High (64) | |
|  | Kalichman 2007 | 213 | Predominantly knee OA (ACR criteria). ROA knee 75%, 41% female, mean age 67 yrs. BOKS | MRI patellar length ratio, trochlea sulcus angle (C) | Cartilage defect (WORMS) (C) | Logistic regression | Low (57) | |
|  | Kim 2013 | 358 | Aging population with 35% ROA knee. 51% female, mean age 72 yrs. Hallym Aging Study | Summary score and severity of MRI BML (WORMS) (C) | WOMAC pain (C) or presence of knee pain | Logistic regression | High (64) | |
|  | Kornaat 2006 | 205 | Symptomatic (35%)and ROA (47%) knee. 80% female, median age 60 yrs. GARP | Semi-quantitative MRI osteophyte  bone cyst & BML (C) | Chronic pain presence (C) | Logistic regression | High (71) | |
|  | Kornaat 2005 | 205 | Symptomatic (35%)and ROA (47%) knee. 80% female, median age 60 yrs. GARP | Semi-quantitative MRI BML (KOSS)  TFJ and PFJ (C) | Semi-quantitative cartilage defects (KOSS) TFJ and PFJ (C) | Odds ratio | Low (57) | |
|  | Kraus 2009 | 159 | Unilateral symptomatic and ROA knee (100%);74% female, mean age 63 yrs, mean BMI 32. POP | Ipsicompartmental late phase bone scintigraphy, semi-quantitative retention scoring of TFJ (C) | Ipsicompartmental OARSI scale of TFJ JSN (C) | Bivariate and Multivariable generalised linear modelling | High (71) | |
|  | Lindsey  2004 | 74 | Prevalent knee ROA (71%), 53% female, mean age 64 yrs, | MRI bone volume fraction & trabecular spacing (lateral TFJ) (C) | Cartilage volume in (contralateral TFJ) compartment (C) | Spearmans correlation | High (64) | |
|  | Link 2003 | 50 | Symptomatic knee OA, ROA 80%, 60% female, mean age 64 yrs. USA | Semi-quantitative MRI BML, osteophytes, and crude presence of bone cysts (C) | KL grade, WOMAC pain (C) | Fisher Exact Test,  Chi-squared test | Low (54) | |
|  | Lo 2005 | 498 | Patients >50 yrs of age with and without knee pain. ROA knee 23%, 59% female, mean age 66 yrs. Framingham OA cohort | Semi-quantitative MRI BML (WORMS≥1) (C) | KL grade≥2 (C) | Crude comparison | Low (50) | |
|  | Lo 2006 | 1612 | Prevalent Knee OA, 18% ROA knee, 56% female, mean age 64 yrs. Framingham OA Study Cohort | DXA Medial:lateral BMD ratio at the tibial plateau (C) | Radiographic JSN grade (medial and lateral TFJ) (C) | Logistic regression | High (71) | |
|  | Lo 2009 | 160 | Symptomatic OA knee, 100% ROA. 50% female, mean age 61 yrs. OAI | Semi-quantitative MRI BML (BLOKS) (C) | WOMAC pain (C) | Univariate and  multivariate cox regressions | High (71) | |
|  | Lo 2012 | 482 | Prevalent knee OA, 54% ROA, 47% female, mean age 64 yrs. OAI | MRI bone volume fraction, trabecular thickness, number and DXA BMD of proximal medial tibia (C) | Radiographic medial JSN grade (C) | Kruskal-Wallis and Mann-Whitney U tests | High (64) | |
|  | McCauley 2001 | 193 | Knees referred for MRI  43% female, mean age 40 yrs, mean weight 92kg. | MRI central osteophyte presence (C) TFJ | MRI cartilage lesion presence (C) TFJ | Crude association | Low (29) | |
|  | McCrae  1992 | 30 | Clinical or ROA knee (100%). 73% female, mean age 66yrs, Overweight or obese (65%). Recruited from rheumatology clinic | Late phase ‘extended bone uptake’ pattern bone scintigraphy, presence around the TFJ (C) | Radiographic TFJ JSN presence (C) | Chi squared test | Low (50) | |
|  | Meredith 2009 | 140 | Knees with MRIs before arthroscopic partial meniscectomy. Median age 61 yrs, 61% female | Sum of semi-quantitative MRI  Osteophyte and BML scores in the TFJ and PFJ (C) | Sum of semi-quantitative MRI  cartilage defect scores in the TFJ and PFJ (C) | Chi-squared test &  Spearman test for non-parametric correlations | Low (50) | |
|  | Moisio 2009 | 305 | Patients with some WOMAC dysfunction and 90% ROA knee. 78% female, mean age 66 yrs. MAK-2 | Baseline MRI semi-quantitative BML score (C) TFJ and PFJ | Presence of baseline moderate to severe knee pain (C) | Logistic regression | High (64) | |
|  | Ochiai 2010 | 48 | Patients with clinical medial knee OA and ROA knee (76%), mean age 73 yrs. Gender distribution unknown. Japan | MRI irregularity of femoral condyle contour (C) | Knee pain VAS (C) | Pearson’s correlation | Low (50) | |
|  | Okazaki 2014 | 29 | Radiographic knee OA (100%), 100% female, Mean age 65 yrs | Number of CT bone cysts (medial femur and tibia) (C) | Knee KL grade (C) | NR | Low (50) | |
|  | Ratzlaff 2013 | 115 | Radiographic knee OA (95%), 48% female, age range 45-79 yrs | Total BML volume in the femur or tibia (C) | Weight bearing knee pain WOMAC subscale (C) | Wilcoxon rank sum test, multivariable analysis, | High (71) | |
|  | Ratzlaff 2014 abstract | 115 | Knee ROA (90%) | Median BML volume (PFJ, TFJ) (C) | Stair-climbing knee pain WOMAC (C) | Wilcoxon rank sum test | High (64) | |
|  | Reichenbach 2008 | 964 | Patients over 50 yrs of age with and without knee pain. ROA knee 18%, 57% female, mean age 63 yrs. Framingham OA cohort | Semi-quantitative MRI Bone attrition (WORMS) (C) | KL grade and semi-quantitative cartilage defects (WORMS) (C) | Crude comparison | Low (43) | |
|  | Roemer 2012 | 1248 | Patients over 50 yrs of age with and without knee pain. ROA knee 23%, 58% female, mean age 64 yrs. Framingham OA cohort | MRI osteophyte  (WORMS) (C) | Cartilage defect (WORMS) (C) | Logistic regression and generalised estimating equations | Low (57) | |
|  | Scher 2008 | 73 | Patients with OA knee based upon radiography (>50% ROA), 54% female, mean age 51 yrs. USA | Semi-quantitative MRI BML (C) | Semi-quantitative cartilage defect (modified Noyes) (C) | Univariate comparison (t-tests) | Low (43) | |
|  | Sengupta 2006 | 217 | Patients with prevalent knee OA (ACR criteria). ROA knee >75%, 25% female, mean age 67 yrs. BOKS | Semi-quantitative MRI Osteophyte (WORMS) (C) | Pain severity WOMAC, chronic pain (C) | Logistic regression | High (71) | |
|  | Sharma 2014 | 837 | At risk of Knee OA but without ROA knee, 0% ROA knee, 57% female, mean age 60 yrs. OAI | Semi-quantitative MRI BML (WORMS)  TFJ or PFJ (C) | Prevalent frequent knee symptoms (C) | Multiple logistic regression | High (71) | |
|  | Sowers 2003 | 231 | Patients with infrequent OA knee symptoms and 15% ROA knee, 100% female, mean age 47 yrs. SWAN | Semi-quantitative MRI BML (C) | Semi-quantitative cartilage defect, chronic pain presence (C) | Wilcoxon or Maentel–  Haenszel test of general association and logistic regression | Low (54) | |
|  | Stefanik 2012 | 881 | Patients with knee OA or at high risk of it. (ROA knee % unknown), 63% female, mean age 63 yrs. MOST | MRI lateral trochlear inclination and trochlear angle (C) | Semi-quantitative cartilage defect (WORMS) (C) | Logistic regression | Low (57) | |
|  | Stefanik 2014 abstract | 2087 | Prevalent clinical knee OA, 60% female, mean age 67 yrs | BML (WORMS)  PFJ (C) | Prevalent knee pain (any pain in last 30 days) and pain VAS (C) | Logistic regression | High (71) | |
|  | Stehling 2010 | 236 | Knees without pain and with 6% ROA. Mean age 51yrs, 58% female, mean BMI 24. OAI | Presence of any MRI semi-quantitative BMLs, osteophytes or  cysts (C) | Presence of any WORMS MRI cartilage defects  (C) | Multi-variate regression | High (71) | |
|  | Torres 2006 | 143 | Patients with some WOMAC dysfunction and >55% ROA knee, 78% female, mean age 70 yrs. MAK-2 | MRI BML, osteophyte, attrition, bone cyst (WORMS) TFJ & PFJ (C) | Pain VAS, semi-quantitative cartilage (WORMS) TFJ & PFJ (C) | Median quantile regression, | High (68) | |
|  | Wang 2005 | 117 | Symptomatic, clinical (ACR) knee OA with mild to moderate TFJ ROA, mean age 64yrs, mean BMI 29, males and females. Australia | Annual % change in tibial bone area (L)  2 yr follow up. | Baseline JSN (C) | Linear regression | Low (57) | |
|  | Zhai 2006 | 500 | Randomly selected older adults with over 23% knee ROA. 50% female, mean age 63 yrs. TASOAC | Semi-quantitative MRI BML (C) | WOMAC pain>1 (C) | Multivariable analysis | High (79) | |
|  | Hip cohort studies | | | | | | | |
|  | Agricola 2013 | 723 | Early symptomatic hip OA, the majority had no ROA hip (with doubtful ROA in 26%), 80% female, mean age 56 yrs, mean BMI 26.1, CHECK | Baseline alpha angle (2D femur shape) dichotomous abnormal >60°, normal ≤60  (C) | Incident ROA hip (KL>1)  Incident end-stage ROA hip  (KL>2 or THR)  at or within 5 yrs (L) | Generalised estimating equations | High (67) | |
|  | Agricola 2013 | 720 | Early symptomatic hip OA, the majority had no ROA hip (with doubtful ROA in 24%), 79% female, mean age 56yrs, mean BMI 26.1. CHECK | Baseline 2D Centre edge angle (Acetabular shape):  25°<Normal<40°  Undercoverage<25°  Overcoverage>40°  (C) | Incident ROA hip (KL>1 or THR)  at or within 5 yrs (L) | Generalised estimating equations | High (67) | |
|  | Agricola 2013 | 723 | Early symptomatic hip OA, the majority had no ROA hip (with doubtful ROA in 24%), 79% women, mean age 56yrs, mean BMI 26.1. CHECK | Baseline 2D femoral and acetabular shape modes (segmented by statistical shape modelling)  (C) | Total hip replacement  at or within 5 yrs (L) | Generalised estimating equations | High (72) | |
|  | Hip case-control studies | | | | | | | |
|  | Doherty  2008 | 2076 | Symptomatic radiographic hip OA cases (n=965), asymptomatic controls without radiographic hip OA (n=1111) – GOAL | Non-spherical 2D femoral head shape assessment:   1. Appearance of ‘Pistol grip deformity’ (C)   2) Maximum femoral head diameter divided by minimum parallel femoral neck diameter (C) | Presence of radiographic hip OA  (JSW≤2.5mm) (C) | Multivariable Logistic regression | Low (53) | |
|  | Barr 2012 | 141 | First presentation of hip pain to primary care, 32% ACR hip OA criteria, 68% female, mean age 63 yrs, mean BMI 27. | 2D Shape measures of centre edge angle (acetabular shape) (C) | THR vs no radiographic progression over 5 years (L) | Logistic regression | High (76) | |
|  | Nicholls 2011 | 268 | 100% women, mean age, 55yrs, mean BMI 66. 243 controls, 25 cases of total hip replacement.  Chingford Study | 2D CAM deformity; mean modified triangular index height, alpha angle.  Acetabular dysplasia;  mean lateral center edge angle (C) | Total hip replacement  (L) | Logistic regression | High (71) | |
|  | Hip cross-sectional studies | | | | | | | |
|  | Chaganti  2010 | 3529 | 10% ROA hip, 100% male, Mean age 78 years. Cohort of the Study of Osteoporotic Fractures in Men | Femoral neck BMD (C) DXA | Hip ROA Modified croft score (categorical 0-4) (C) | Linear regression | High (64) | |
|  | Chiba 2011 | 47 | ROA, 100% female, mean age 69 yrs | Acetabular and femoral head subchondral trabecular morphometry:  bone volume fraction,  trabecular thickness, number, separation (CT) | Hip joint space volume (CT) (C) | Pearson’s correlation test. | Low (57) | |
|  | Dawson 2013  Abstract | 161 | 142 asymptomatic hips without clinical OA, 19 with hip OA. 56% female, mean age 63yrs, mean BMI 27, | Femoral head BMLs  (MRI) (C) | 1. Presence of hip OA  2.Femoral head cartilage volume (MRI) (C) | Regression modelling | Low (14) | |
|  | Gosvig  2010 | 3620 | Mean age 61yrs, 63% female, ROA hip 10.6%  (OA substudy - CCHS III) | 2D Categorical Hip deformity:  1) Normal  2)‘Pistol grip’  3) Deep acetabular socket  (C) | Presence of radiographic hip OA  (JSW≤2mm) (C) | Multivariate logistic regression | Low (50) | |
|  | Maksymowych 2014 | 40 | 55% female, mean age 65yrs, symptomatic but not radiographic hip OA | Semi-quantitative BML HIP  (HOAMS) (C) | Baseline WOMAC pain (C) | Univariable regression model | High (64) | |
|  | Neumann 2007 | 100 | Symptomatic hip OA | Semi-quantitative BMLs (C) | Semi-quantitative Cartilage lesions (C) | Spearman’s correlations | Low (43) | |
|  | Reichenbach 2011 | 244 | Asymptomatic men (100%). Mean age 20 yrs, mean BMI 23, Sumiswald cohort | The presence or absence of any semi-quantitative MRI-defined CAM-deformity  (C) | Combined femoral and acetabular cartilage thickness  (C) | Multivariable linear regression.  &  Wald test | High (64) | |
|  | Antoniades 2000 | 1148 | White female twins, 29% hip RO  A, 100% female, median age 53 yrs. St. Thomas’ UK Adult Twin Register (C) | DXA BMD  of the femoral neck of left (nondominant) hip with ROA (C) | Radiographic OA (croft Score) (C) | Logistic regression | High (64) | |
|  | Kumar 2013 | 85 | Members of the public with 35% ROA hip, 48% female , mean age 56 yrs (C) | Total hip semi-quantitative BML and subchondral cysts score (C) | Self-reported hip pain  HOOS score | Non-parametric Spearman’s correlations | High (71) | |
|  | Hand case-series studies | | | | | | | |
|  | Haugen 2014 | 74 | 91% female, Mean age 68 yrs | BMLs – semi-quantitative  At 2^nd^ to 5^th^ IPJs (C) | Progression of hand ROA (JSN, KL grade or new erosion) (L) | Generalised estimating equations | High (61) | |
|  | Haugen 2014  Abstract | 70 | 90% female, mean age 68 yrs | Sum scores (0-48) for BMLs (Oslo Hand OA MRI score) (C) IPJS | AUSCAN pain scale (L) | Linear regression | High (61) | |
|  | Hand cross-sectional studies | | | | | | | |
|  | Haugen 2012  Abstract | 108 | 91% women, mean age 69 years, 100% ROA hand Oslo hand osteoarthritis cohort | BML (Oslo MRI hand score) (C) IPJs | Radiographic JSN grade (OARSI atlas) (C) IPJs | logistic regression  with generalised estimating equations | Low (43) | |
|  | Haugen  2012 | 106 | 92% women, mean age 69 years, 100% ROA Oslo hand osteoarthritis cohort | BML, cyst, attrition, osteophyte (Oslo MRI hand score) (C)  IPJs | Hand KL grade of IPJs (C) | Generalised estimating equations | High (64) | |
|  | Haugen 2012 | 85 | 91% female, mean age 69 years, 100% ROA hand. Oslo hand osteoarthritis cohort | BML, cyst, attrition, osteophyte (Oslo MRI hand score) (C)  IPJs sum scores | AUSCAN pain scale (C) | Linear regression | High (64) | |
|  | Macfarlane 1993 | 35 | 100% ROA Hand, 91% female, mean age 62yrs. Rheumatology clinic attenders | Late phase isotope bone scan small joints of the hand  (C) | Hand Pain VAS (C) | Kendall’s correlation | Low (57) | |
|  | Ankle cross-sectional studies | | | | | | | |
|  | Knupp 2009 | 27 | Symptomatic ankle varus or valgus deformities refractory to conservative therapy, 37% female, mean age 49 yrs. | Late phase bone scintigraphy, semi-quantitative retention scoring of tibiotalar joint (C) | Tibiotalar ankle joint JSN. (Modified Takakura score)  (C) | Mann-Whitney Rank sum test | Low (57) | |
|  | Kraus 2013 | 138 | Symptomatic ankle OA (23%), ROA ankle (79%), 74% female, mean age 64yrs, mean BMI 31. POP | Ipsilateral late phase bone scintigraphy, retention presence in tibiotalar joint (C) | Tibiotalar ROA KL grade and JSN (C) | Generalized estimating equations | High (71) | |

Australian/Canadian Osteoarthritis Hand Index (AUSCAN); Bone mineral density (BMD); Bone marrow lesion (BML); Boston Osteoarthritis of the Knee Study (BOKS), Boston–Leeds. Osteoarthritis Knee Score (BLOKS); a feature or outcome described in cross-section (C); Copenhagen City Heart Study (CCHS); Cohort hip and cohort knee (CHECK); knee pain on most days for at least the last month (chronic pain); Dual-energy X-ray absorptiometry (DXA); Genetics, Osteoarthritis and Progression study (GARP); GOAL (Genetics of Osteoarthritis and Lifestyle); Hip Osteoarthritis MRI scoring system (HOAMS); Hip dysfunction and Osteoarthritis Outcome Score (HOOS); interphalangeal joint (IPJ); joint space narrowing (JSN); joint space width (JSW); Kellgren Lawrence (KL); Knee Osteoarthritis Scoring System (KOSS); a feature or outcome described longitudinally (L); mechanical factors in arthritis of the knee 2 (MAK-2); patellofemoral joint (PFJ); quantitative computed tomography (qCT); radiographic osteoarthritis (ROA); Michigan study of Women’s Health across the Nation (SWAN); Multicentre Osteoarthritis Study (most); osteoarthritis (OA); Osteoarthritis Initiative (OAI); Osteoarthritis Research Society International (OARSI); Strategies to Predict Osteoarthritis Progression (POP); Tasmanian Older Adult Cohort (TASOAC); tibiofemoral joint (TFJ); Total hip replacement (THR); visual analogue scale (VAS); Western Ontario and McMaster Universities arthritis index (WOMAC); whole-organ magnetic resonance imaging score (WORMS).

# References

1. Lievense AM, Bierma-Zeinstra SM, Verhagen AP, van Baar ME, Verhaar JA, Koes BW. Influence of obesity on the development of osteoarthritis of the hip: a systematic review. Rheumatology (Oxford). 2002;41(10):1155-62.

2. Yusuf E, Kortekaas MC, Watt I, Huizinga TW, Kloppenburg M. Do knee abnormalities visualised on MRI explain knee pain in knee osteoarthritis? A systematic review. Ann Rheum Dis. 2011;70(1):60-7.

3. Ai F, Yu C, Zhang W, Morelli JN, Kacher D, Li X. MR imaging of knee osteoarthritis and correlation of findings with reported patient pain. Journal of Huazhong University of Science and Technology - Medical Science. 2010;30(2):248-54.

4. Akamatsu Y, Kobayashi H, Kusayama Y, Kumagai K, Mitsugi N, Saito T. Does subchondral sclerosis protect progression of joint space narrowing in patients with varus knee osteoarthritis? Osteoarthritis Cartilage. 2014;22:S362.

5. Antoniades L, MacGregor AJ, Matson M, Spector TD. A cotwin control study of the relationship between hip osteoarthritis and bone mineral density. Arthritis Rheum. 2000;43(7):1450-5.

6. Baranyay FJ, Wang Y, Wluka AE, English DR, Giles GG, Sullivan RO, et al. Association of bone marrow lesions with knee structures and risk factors for bone marrow lesions in the knees of clinically healthy, community-based adults. Semin Arthritis Rheum. 2007;37(2):112-8.

7. Bilgici A, Dogan C, Cil E, Sakarya S, Kuru O, Selcuk MB. Relationship between pain severity and magnetic resonance imaging features in patients with osteoarthritis of the Knee [Turkish]. Turkish Journal of Rheumatology. 2010;25(4):184-90.

8. Burnett WK, SA. McLennan, CE. Wheaton, D. Talmo, C. Hunter, DJ. Wilson, DR. Johnston, JD. Patella Bone density is lower in knee osteoarthritis patients experiencing pain at rest. Osteoarthritis Cartilage. 2012;20(S200-S201).

9. Chaganti RK, Parimi N, Lang T, Orwoll E, Stefanick ML, Nevitt M, et al. Bone mineral density and prevalent osteoarthritis of the hip in older men for the Osteoporotic Fractures in Men (MrOS) Study Group. Osteoporos Int. 2010;21(8):1307-16.

10. Chiba K, Ito M, Osaki M, Uetani M, Shindo H. In vivo structural analysis of subchondral trabecular bone in osteoarthritis of the hip using multi-detector row CT. Osteoarthritis Cartilage. 2011;19(2):180-5.

11. Chiba K, Uetani M, Kido Y, Ito M, Okazaki N, Taguchi K, et al. Osteoporotic changes of subchondral trabecular bone in osteoarthritis of the knee: a 3-T MRI study. Osteoporosis International. 2012;23(2):589-97.

12. Crema MD, Roemer FW, Marra MD, Niu J, Lynch JA, Felson DT, et al. Contrast-enhanced MRI of subchondral cysts in patients with or at risk for knee osteoarthritis: the MOST study. European Journal of Radiology. 2010;75(1):e92-6.

13. Dawson L, Bennell, K., Wluka, A., Wang, Y., Cicuttini, F. Hip bone marrow lesions in asymptomatic and osteoarthritic adults: Prevalence, risk factors and significance. Osteoarthritis and cartilage / OARS, Osteoarthritis Research Society. 2013;21(Suppl):S241.

14. Ding C, Garnero P, Cicuttini F, Scott F, Cooley H, Jones G. Knee cartilage defects: association with early radiographic osteoarthritis, decreased cartilage volume, increased joint surface area and type II collagen breakdown. Osteoarthritis Cartilage. 2005;13(3):198-205.

15. Dore D, Quinn S, Ding C, Winzenberg T, Jones G. Correlates of subchondral BMD: a cross-sectional study. J Bone Miner Res. 2009;24(12):2007-15.

16. Driban JB, Price LL, Tassinari AM, Lo GH, McAlindon TE. Peri-articular apparent bone volume fraction is associated with numerous patient characteristics in knees with osteoarthritis: Data from the osteoarthritis initiative. Arthritis Rheum. 2011;63(10 SUPPL. 1).

17. Driban JB, Price LL, Tassinari AM, Lo GH, Schneider E, McAlindon TE. Trabecular morphology is associated with numerous patient characteristics in knees with osteoarthritis: Data from the osteoarthritis initiative (OAI). Osteoarthritis Cartilage. 2011;19:s169.

18. Eckstein F, Wirth W, Hunter DJ, Guermazi A, Kwoh CK, Nelson DR, et al. Magnitude and regional distribution of cartilage loss associated with grades of joint space narrowing in radiographic osteoarthritis - data from the Osteoarthritis Initiative (OAI). Osteoarthritis Cartilage. 2010;18(6):760-8.

19. Felson DT, Chaisson CE, Hill CL, Totterman SM, Gale ME, Skinner KM, et al. The association of bone marrow lesions with pain in knee osteoarthritis. Ann Intern Med. 2001;134(7):541-9.

20. Fernandez-Madrid F, Karvonen RL, Teitge RA, Miller PR, Negendank WG. MR features of osteoarthritis of the knee. Magn Reson Imaging. 1994;12(5):703-9.

21. Frobell RB, Nevitt MC, Hudelmaier M, Wirth W, Wyman BT, Benichou O, et al. Femorotibial subchondral bone area and regional cartilage thickness: a cross-sectional description in healthy reference cases and various radiographic stages of osteoarthritis in 1,003 knees from the Osteoarthritis Initiative. Arthritis Care Res. 2010;62(11):1612-23.

22. Gosvig KK, Jacobsen S, Sonne-Holm S, Palm H, Troelsen A. Prevalence of malformations of the hip joint and their relationship to sex, groin pain, and risk of osteoarthritis: a population-based survey. J Bone Joint Surg Am. 2010;92(5):1162-9.

23. Gudbergsen H, Lohmander LS, Jones G, Christensen R, Bartels EM, Danneskiold-Samsoe B, et al. Correlations between radiographic assessments and MRI features of knee osteoarthritis - a cross-sectional study. Osteoarthritis Cartilage. 2013;21(4):535-43.

24. Guymer E, Baranyay F, Wluka AE, Hanna F, Bell RJ, Davis SR, et al. A study of the prevalence and associations of subchondral bone marrow lesions in the knees of healthy, middle-aged women. Osteoarthritis Cartilage. 2007;15(12):1437-42.

25. Haugen IK, Boyesen P, Slatkowsky-Christensen B, Sesseng S, van der Heijde D, Kvien TK. Associations between MRI-defined synovitis, bone marrow lesions and structural features and measures of pain and physical function in hand osteoarthritis. Ann Rheum Dis. 2012;71(6):899-904.

26. Haugen IK. BP, Slatkowsky-Christensen B., Sesseng S., van der Heijde D., Kiven TK. Associations between radiographic and clinical osteoarthritis features and mri-defined bone marrow lesions in the finger joints. Annals of the Rheumatic Diseases. 2012;71(Suppl 3):299.

27. Haugen IK, Boyesen P, Slatkowsky-Christensen B, Sesseng S, Bijsterbosch J, van der Heijde D, et al. Comparison of features by MRI and radiographs of the interphalangeal finger joints in patients with hand osteoarthritis. Ann Rheum Dis. 2012;71(3):345-50.

28. Haverkamp DJ, Schiphof D, Bierma-Zeinstra SM, Weinans H, Waarsing JH. Variation in joint shape of osteoarthritic knees. Arthritis Rheum. 2011;63(11):3401-7.

29. Hayashi D, Xu L, Roemer FW, Hunter DJ, Li L, Katur AM, et al. Detection of osteophytes and subchondral cysts in the knee with use of tomosynthesis. Radiology. 2012;263(1):206-15.

30. Hayes CW, Jamadar DA, Welch GW, Jannausch ML, Lachance LL, Capul DC, et al. Osteoarthritis of the knee: comparison of MR imaging findings with radiographic severity measurements and pain in middle-aged women. Radiology. 2005;237(3):998-1007.

31. Hernandez-Molina G, Neogi T, Hunter DJ, Niu J, Guermazi A, Reichenbach S, et al. The association of bone attrition with knee pain and other MRI features of osteoarthritis. Ann Rheum Dis. 2008;67(1):43-7.

32. Ip S, Sayre EC, Guermazi A, Nicolaou S, Wong H, Thorne A, et al. Frequency of bone marrow lesions and association with pain severity: results from a population-based symptomatic knee cohort. J Rheumatol. 2011;38(6):1079-85.

33. Jones G, Ding C, Scott F, Glisson M, Cicuttini F. Early radiographic osteoarthritis is associated with substantial changes in cartilage volume and tibial bone surface area in both males and females. Osteoarthritis Cartilage. 2004;12(2):169-74.

34. Kalichman L, Zhang Y, Niu J, Goggins J, Gale D, Zhu Y, et al. The association between patellar alignment on magnetic resonance imaging and radiographic manifestations of knee osteoarthritis. Arthritis Research & Therapy. 2007;9(2).

35. Kalichman L, Zhang Y, Niu J, Goggins J, Gale D, Felson DT, et al. The association between patellar alignment and patellofemoral joint osteoarthritis features - An MRI study. Rheumatology. 2007;46(8):1303-8.

36. Kim IJ, Kim DH, Jung JY, Song YW, Guermazi A, Crema MD, et al. Association between bone marrow lesions detected by magnetic resonance imaging and knee pain in community residents in Korea. Osteoarthritis Cartilage. 2013;21(9):1207-13.

37. Knupp M, Pagenstert GI, Barg A, Bolliger L, Easley ME, Hintermann B. SPECT-CT compared with conventional imaging modalities for the assessment of the varus and valgus malaligned hindfoot. Journal of Orthopaedic Research. 2009;27(11):1461-6.

38. Kornaat PR, Bloem JL, Ceulemans RY, Riyazi N, Rosendaal FR, Nelissen RG, et al. Osteoarthritis of the knee: association between clinical features and MR imaging findings. Radiology. 2006;239(3):811-7.

39. Kornaat PR, Watt I, Riyazi N, Kloppenburg M, Bloem JL. The relationship between the MRI features of mild osteoarthritis in the patellofemoral and tibiofemoral compartments of the knee. European Radiology. 2005;15(8):1538-43.

40. Kraus VB, McDaniel G, Worrell TW, Feng S, Vail TP, Varju G, et al. Association of bone scintigraphic abnormalities with knee malalignment and pain. Annals of the rheumatic diseases. 2009;68(11):1673.

41. Kraus VB, Worrell TW, Renner JB, Coleman RE, Pieper CF. High prevalence of contralateral ankle abnormalities in association with knee osteoarthritis and malalignment. Osteoarthritis and cartilage / OARS, Osteoarthritis Research Society. 2013;21(11):1693.

42. Kumar D, Wyatt CR, Lee S, Nardo L, Link TM, Majumdar S, et al. Association of cartilage defects, and other MRI findings with pain and function in individuals with mild-moderate radiographic hip osteoarthritis and controls. Osteoarthritis Cartilage. 2013;21(11):1685-92.

43. Lindsey CT, Narasimhan A, Adolfo JM, Jin H, Steinbach LS, Link T, et al. Magnetic resonance evaluation of the interrelationship between articular cartilage and trabecular bone of the osteoarthritic knee. Osteoarthritis Cartilage. 2004;12(2):86-96.

44. Link TM, Steinbach LS, Ghosh S, Ries M, Lu Y, Lane N, et al. Osteoarthritis: MR imaging findings in different stages of disease and correlation with clinical findings. Radiology. 2003;226(2):373-81.

45. Lo GH, Hunter DJ, Zhang Y, McLennan CE, Lavalley MP, Kiel DP, et al. Bone marrow lesions in the knee are associated with increased local bone density. Arthritis Rheum. 2005;52(9):2814-21.

46. Lo GH, McAlindon TE, Niu J, Zhang Y, Beals C, Dabrowski C, et al. Bone marrow lesions and joint effusion are strongly and independently associated with weight-bearing pain in knee osteoarthritis: data from the osteoarthritis initiative. Osteoarthritis Cartilage. 2009;17(12):1562-9.

47. Lo GH, Tassinari AM, Driban JB, Price LL, Schneider E, Majumdar S, et al. Cross-sectional DXA and MR measures of tibial periarticular bone associate with radiographic knee osteoarthritis severity. Osteoarthritis Cartilage. 2012;20(7):686-93.

48. Lo GH, Zhang Y, McLennan C, Niu J, Kiel DP, McLean RR, et al. The ratio of medial to lateral tibial plateau bone mineral density and compartment-specific tibiofemoral osteoarthritis. Osteoarthritis Cartilage. 2006;14(10):984-90.

49. Macfarlane DG, Buckland-Wright JC, Lynch J, Fogelman L. A study of the early and late 99technetium scintigraphic images and their relationship to symptoms in osteoarthritis of the hands. Br J Rheumatol. 1993;32(11):977-81.

50. Maksymowych WP, Cibere J, Loeuille D, Weber U, Zubler V, Roemer FW, et al. Preliminary validation of 2 magnetic resonance image scoring systems for osteoarthritis of the hip according to the OMERACT filter. J Rheumatol. 2014;41(2):370-8.

51. McCauley TR, Kornaat PR, Jee WH. Central osteophytes in the knee: prevalence and association with cartilage defects on MR imaging. AJR. American Journal of Roentgenology. 2001;176(2):359-64.

52. McCrae F, Shouls J, Dieppe P, Watt I. Scintigraphic assessment of osteoarthritis of the knee joint. Annals of the rheumatic diseases. 1992;51(8):938.

53. Meredith DS, Losina E, Neumann G, Yoshioka H, Lang PK, Katz JN. Empirical evaluation of the inter-relationship of articular elements involved in the pathoanatomy of knee osteoarthritis using magnetic resonance imaging. BMC Musculoskeletal Disorders. 2009;10:133.

54. Moisio K, Eckstein F, Chmiel JS, Guermazi A, Prasad P, Almagor O, et al. Denuded subchondral bone and knee pain in persons with knee osteoarthritis. Arthritis Rheum. 2009;60(12):3703-10.

55. Neumann G, Mendicuti AD, Zou KH, Minas T, Coblyn J, Winalski CS, et al. Prevalence of labral tears and cartilage loss in patients with mechanical symptoms of the hip: evaluation using MR arthrography. Osteoarthritis Cartilage. 2007;15(8):909-17.

56. Ochiai N, Sasho T, Tahara M, Watanabe A, Matsuki K, Yamaguchi S, et al. Objective assessments of medial osteoarthritic knee severity by MRI: new computer software to evaluate femoral condyle contours. International Orthopaedics. 2010;34(6):811-7.

57. Okazaki N, Chiba K, Kidera K, Yonekura A, Osaki M. Relationship between subchondral bone cysts, the severity of knee osteoarthritis, and alignments of lower extremities. Osteoarthritis Cartilage. 2014;22:S370-S1.

58. Ratzlaff C, Guermazi A, Collins J, Katz JN, Losina E, Vanwyngaarden C, et al. A rapid, novel method of volumetric assessment of MRI-detected subchondral bone marrow lesions in knee osteoarthritis. Osteoarthritis Cartilage. 2013;21(6):806-14.

59. Ratzlaff C, Russell R, Duryea J. Quantitatively-measured bone marrow lesions in the patellofemoral joint: Distribution and association with pain. Osteoarthritis Cartilage. 2014;22:S247-S8.

60. Reichenbach S, Guermazi A, Niu J, Neogi T, Hunter DJ, Roemer FW, et al. Prevalence of bone attrition on knee radiographs and MRI in a community-based cohort. Osteoarthritis Cartilage. 2008;16(9):1005-10.

61. Reichenbach S, Leunig M, Werlen S, Nuesch E, Pfirrmann CW, Bonel H, et al. Association between cam-type deformities and magnetic resonance imaging-detected structural hip damage: a cross-sectional study in young men. Arthritis Rheum. 2011;63(12):4023-30.

62. Roemer FW, Guermazi A, Niu J, Zhang Y, Mohr A, Felson DT. Prevalence of magnetic resonance imaging-defined atrophic and hypertrophic phenotypes of knee osteoarthritis in a population-based cohort. Arthritis Rheum. 2012;64(2):429-37.

63. Scher C, Craig J, Nelson F. Bone marrow edema in the knee in osteoarthrosis and association with total knee arthroplasty within a three-year follow-up. Skeletal Radiology. 2008;37(7):609-17.

64. Sengupta M, Zhang YQ, Niu JB, Guermazi A, Grigorian M, Gale D, et al. High signal in knee osteophytes is not associated with knee pain. Osteoarthritis Cartilage. 2006;14(5):413-7.

65. Sharma L, Chmiel JS, Almagor O, Dunlop D, Guermazi A, Bathon JM, et al. Significance of preradiographic magnetic resonance imaging lesions in persons at increased risk of knee osteoarthritis. Arthritis Rheumatol. 2014;66(7):1811-9.

66. Sowers MF, Hayes C, Jamadar D, Capul D, Lachance L, Jannausch M, et al. Magnetic resonance-detected subchondral bone marrow and cartilage defect characteristics associated with pain and X-ray-defined knee osteoarthritis. Osteoarthritis Cartilage. 2003;11(6):387-93.

67. Stefanik J, Gross K, Felson D, Niu J, Zhang Y, Lewis C, et al. Does medial patellofemoral osteoarthritis matter? the relation of mri-detected structural damage in the medial and lateral patellofemoral joint to knee pain: the most and framingham osteoarthritis studies. Osteoarthritis Cartilage. 2014;22:S54-S5.

68. Stefanik JJ, Roemer FW, Zumwalt AC, Zhu Y, Gross KD, Lynch JA, et al. Association between measures of trochlear morphology and structural features of patellofemoral joint osteoarthritis on MRI: the MOST study. Journal of Orthopaedic Research. 2012;30(1):1-8.

69. Stehling C, Lane NE, Nevitt MC, Lynch J, McCulloch CE, Link TM. Subjects with higher physical activity levels have more severe focal knee lesions diagnosed with 3T MRI: Analysis of a non-symptomatic cohort of the osteoarthritis initiative. Osteoarthritis Cartilage. 2010;18(6):776-86.

70. Torres L, Dunlop DD, Peterfy C, Guermazi A, Prasad P, Hayes KW, et al. The relationship between specific tissue lesions and pain severity in persons with knee osteoarthritis. Osteoarthritis Cartilage. 2006;14(10):1033-40.

71. Wang Y, Wluka AE, Cicuttini FM. The determinants of change in tibial plateau bone area in osteoarthritic knees: a cohort study. Arthritis Res Ther. 2005;7(3):R687-93.

72. Zhai G, Blizzard L, Srikanth V, Ding C, Cooley H, Cicuttini F, et al. Correlates of knee pain in older adults: Tasmanian Older Adult Cohort Study. Arthritis Rheum. 2006;55(2):264-71.

73. Agricola R, Heijboer MP, Bierma-Zeinstra SM, Verhaar JA, Weinans H, Waarsing JH. Cam impingement causes osteoarthritis of the hip: a nationwide prospective cohort study (CHECK). Ann Rheum Dis. 2013;72(6):918-23.

74. Agricola R, Heijboer MP, Roze RH, Reijman M, Bierma-Zeinstra SM, Verhaar JA, et al. Pincer deformity does not lead to osteoarthritis of the hip whereas acetabular dysplasia does: acetabular coverage and development of osteoarthritis in a nationwide prospective cohort study (CHECK). Osteoarthritis Cartilage. 2013;21(10):1514-21.

75. Agricola R, Reijman M, Bierma-Zeinstra SM, Verhaar JA, Weinans H, Waarsing JH. Total hip replacement but not clinical osteoarthritis can be predicted by the shape of the hip: a prospective cohort study (CHECK). Osteoarthritis Cartilage. 2013;21(4):559-64.

76. Bruyere O, Dardenne C, Lejeune E, Zegels B, Pahaut A, Richy F, et al. Subchondral tibial bone mineral density predicts future joint space narrowing at the medial femoro-tibial compartment in patients with knee osteoarthritis. Bone. 2003;32(5):541-5.

77. Carnes J, Stannus O, Cicuttini F, Ding C, Jones G. Knee cartilage defects in a sample of older adults: natural history, clinical significance and factors influencing change over 2.9 years. Osteoarthritis Cartilage. 2012;20(12):1541-7.

78. Carrino JA, Blum J, Parellada JA, Schweitzer ME, Morrison WB. MRI of bone marrow edema-like signal in the pathogenesis of subchondral cysts. Osteoarthritis Cartilage. 2006;14(10):1081-5.

79. Cicuttini FM, Jones G, Forbes A, Wluka AE. Rate of cartilage loss at two years predicts subsequent total knee arthroplasty: a prospective study. Ann Rheum Dis. 2004;63(9):1124-7.

80. Crema MD, Felson DT, Roemer FW, Wang K, Marra MD, Nevitt MC, et al. Prevalent cartilage damage and cartilage loss over time are associated with incident bone marrow lesions in the tibiofemoral compartments: the MOST study. Osteoarthritis and cartilage / OARS, Osteoarthritis Research Society. 2013;21(2):306-13.

81. Crema MD, Cibere J, Sayre EC, Roemer FW, Wong H, Thorne A, et al. The relationship between subchondral sclerosis detected with MRI and cartilage loss in a cohort of subjects with knee pain: the knee osteoarthritis progression (KOAP) study. Osteoarthritis Cartilage. 2014;22(4):540-6.

82. Davies-Tuck ML, Wluka AE, Wang Y, Teichtahl AJ, Jones G, Ding C, et al. The natural history of cartilage defects in people with knee osteoarthritis. Osteoarthritis Cartilage. 2008;16(3):337-42.

83. Davies-Tuck ML, Wluka AE, Forbes A, Wang Y, English DR, Giles GG, et al. Development of bone marrow lesions is associated with adverse effects on knee cartilage while resolution is associated with improvement--a potential target for prevention of knee osteoarthritis: a longitudinal study. Arthritis Res Ther. 2010;12(1):R10.

84. de_Lange BJ, Ioan-Facsinay A, Bijsterbosch J, Van G, Zuurmond A, Kornaat P, et al. The patellofemoral and femorotibial joints are related based on patterns of MRI features and their association with radiologic progression. Osteoarthritis Cartilage. 2014;22:S254-S5.

85. Dieppe P, Cushnaghan J, Young P, Kirwan J. Prediction of the progression of joint space narrowing in osteoarthritis of the knee by bone scintigraphy. Annals of the rheumatic diseases. 1993;52(8):557.

86. Ding C, Cicuttini F, Scott F, Cooley H, Boon C, Jones G. Natural history of knee cartilage defects and factors affecting change. Arch Intern Med. 2006;166(6):651-8.

87. Ding C, Martel-Pelletier J, Pelletier JP, Abram F, Raynauld JP, Cicuttini F, et al. Two-year prospective longitudinal study exploring the factors associated with change in femoral cartilage volume in a cohort largely without knee radiographic osteoarthritis. Osteoarthritis Cartilage. 2008;16(4):443-9.

88. Dore D, Quinn S, Ding C, Winzenberg T, Zhai G, Cicuttini F, et al. Natural history and clinical significance of MRI-detected bone marrow lesions at the knee: a prospective study in community dwelling older adults. Arthritis Res Ther. 2010;12(6):R223.

89. Dore D, Martens A, Quinn S, Ding C, Winzenberg T, Zhai G, et al. Bone marrow lesions predict site-specific cartilage defect development and volume loss: a prospective study in older adults. Arthritis Res Ther. 2010;12(6):R222.

90. Dore D, Quinn S, Ding C, Winzenberg T, Cicuttini F, Jones G. Subchondral bone and cartilage damage: a prospective study in older adults. Arthritis Rheum. 2010;62(7):1967-73.

91. Driban JB, Lo GH, Lee J, Ward RJ, Miller E, Pang J, et al. Quantitative bone marrow lesion size in osteoarthritic knees correlates with cartilage damage and predicts longitudinal cartilage loss. BMC Musculoskeletal Disorders. 2011;12.

92. Driban JB, Pang J, Miller E, Destenaves G, Lo GH, Ward RJ, et al. Quantitative bone marrow lesion changes relate to cartilage parameter changes. Osteoarthritis Cartilage. 2012;20:S217-S8.

93. Driban JB, Price LL, Lo GH, Pang J, Hunter DJ, Miller E, et al. Evaluation of bone marrow lesion volume as a knee osteoarthritis biomarker - longitudinal relationships with pain and structural changes: Data from the Osteoarthritis Initiative. Arthritis Research and Therapy. 2013;15(5).

94. Everhart JS, Siston RA, Flanigan DC. Tibiofemoral subchondral surface ratio (SSR) is a predictor of osteoarthritis symptoms and radiographic progression: data from the Osteoarthritis Initiative (OAI). Osteoarthritis Cartilage. 2014;22(6):771-8.

95. Felson DT, McLaughlin S, Goggins J, LaValley MP, Gale ME, Totterman S, et al. Bone marrow edema and its relation to progression of knee osteoarthritis. Ann Intern Med. 2003;139(5 Pt 1):330-6.

96. Foong YC, Khan HI, Blizzard L, Ding C, Cicuttini F, Jones G, et al. The clinical significance, natural history and predictors of bone marrow lesion change over eight years. Arthritis Res Ther. 2014;16(4):R149.

97. Guermazi A, Eckstein F, Hayashi D, Roemer FW, Wirth W, Yang T, et al. Cartilage damage, bone marrow lesions and meniscal lesions predict quantitatively measured loss of cartilage over 30-months: The most study. Osteoarthritis Cartilage. 2014;22:S356.

98. Haugen IK, Slatkowsky-Christensen B, Boyesen P, Sesseng S, van der Heijde D, Kvien TK. MRI findings predict radiographic progression and development of erosions in hand osteoarthritis. Ann Rheum Dis. 2014.

99. Haugen IK. KE, Slatkowsky-Christensen B., Sesseng S., Kiven TK. Predictive value of mri-defined synovitis, bone marrow lesions and central erosions on pain and physical function in hand osteoarthritis. Osteoarthritis Cartilage. 2014;22:S386.

100. Hernandez-Molina G, Guermazi A, Niu J, Gale D, Goggins J, Amin S, et al. Central bone marrow lesions in symptomatic knee osteoarthritis and their relationship to anterior cruciate ligament tears and cartilage loss. Arthritis Rheum. 2008;58(1):130-6.

101. Hochberg MC, Yip A, Favors K, Sorkin J, Martel-Pelletier J, Pelletier JP. Features assessed on magnetic resonance images improve prediction of total knee arthroplasty in subjects with symptomatic radiographic knee osteoarthritis: Data from the osteoarthritis initiative. Osteoarthritis Cartilage. 2014;22:S175.

102. Hudelmaier M, Wirth W, Nevitt M, Eckstein F. Longitudinal rates of change in subchondral bone size in healthy knees and knees with radiographic osteoarthritis. Osteoarthritis Cartilage. 2013;21(April Suppl):S242.

103. Hunter DJ, Zhang Y, Niu J, Goggins J, Amin S, LaValley MP, et al. Increase in bone marrow lesions associated with cartilage loss: a longitudinal magnetic resonance imaging study of knee osteoarthritis. Arthritis Rheum. 2006;54(5):1529-35.

104. Kornaat PR, Kloppenburg M, Sharma R, Botha-Scheepers SA, Le MPM, Coene LN, et al. Bone marrow edema-like lesions change in volume in the majority of patients with osteoarthritis; associations with clinical features. European Radiology. 2007;17(12):3073-8.

105. Koster IM, Oei EHG, Hensen JHJ, Boks SS, Koes BW, Vroegindeweij D, et al. Predictive factors for new onset or progression of knee osteoarthritis one year after trauma: MRI follow-up in general practice. European Radiology. 2011;21(7):1509-16.

106. Kothari A, Guermazi A, Chmiel JS, Dunlop D, Song J, Almagor O, et al. Within-subregion relationship between bone marrow lesions and subsequent cartilage loss in knee osteoarthritis. Arthritis Care Res. 2010;62(2):198-203.

107. Kubota M, Ishijima M, Kurosawa H, Liu L, Ikeda H, Osawa A, et al. A longitudinal study of the relationship between the status of bone marrow abnormalities and progression of knee osteoarthritis. Journal of Orthopaedic Science. 2010;15(5):641-6.

108. Liu L, Kaneko H, Sadatsuki R, Hada S, Yusup A, Kinoshita M, et al. MRI-detected osteophyte is a predictor for receiving total knee arthroplasty in patients with end-stage knee osteoarthritis. Osteoarthritis Cartilage. 2014;22:S470-S1.

109. Lo GH, Schneider E, Price L, Driban J, Tassinari A, Nevitt M, et al. Periarticular bone density and trabecular morphology predict knee OA structural progression. Osteoarthritis Cartilage. 2012;20:S76.

110. Madan-Sharma R, Kloppenburg M, Kornaat PR, Botha-Scheepers SA, Le MPH, Bloem JL, et al. Do MRI features at baseline predict radiographic joint space narrowing in the medial compartment of the osteoarthritic knee 2 years later? Skeletal Radiology. 2008;37(9):805-11.

111. Mazzuca A, Brandt D, Schauwecker S, Buckwalter A, Katz P, Meyer M, et al. Bone scintigraphy is not a better predictor of progression of knee osteoarthritis than Kellgren and Lawrence grade. The Journal of rheumatology. 2004;31(2):329-32.

112. Mazzuca A, Brandt D, Schauwecker S, Katz P, Meyer M, Lane A, et al. Severity of joint pain and Kellgren-Lawrence grade at baseline are better predictors of joint space narrowing than bone scintigraphy in obese women with knee osteoarthritis. The Journal of rheumatology. 2005;32(8):1540-6.

113. Parsons C. EMH, Bruye`re O., Belissa P., Genant H.K., Guermazi A., Roemer F., Zaim S., Reginster J.-Y., Dennison E.M., Cooper C. Impact of bone marrow lesion on the progression of knee osteoarthritis in the sekoia study. Rheumatology. 2014;53(Suupl 1):i130.

114. Pelletier JP, Raynauld JP, Berthiaume MJ, Abram F, Choquette D, Haraoui B, et al. Risk factors associated with the loss of cartilage volume on weight-bearing areas in knee osteoarthritis patients assessed by quantitative magnetic resonance imaging: a longitudinal study. Arthritis Research & Therapy. 2007;9(4).

115. Raynauld JP, Martel-Pelletier J, Berthiaume MJ, Abram F, Choquette D, Haraoui B, et al. Correlation between bone lesion changes and cartilage volume loss in patients with osteoarthritis of the knee as assessed by quantitative magnetic resonance imaging over a 24-month period. Annals of the Rheumatic Diseases. 2008;67(5):683-8.

116. Raynauld JP, Martel-Pelletier J, Haraoui B, Choquette D, Dorais M, Wildi LM, et al. Risk factors predictive of joint replacement in a 2-year multicentre clinical trial in knee osteoarthritis using MRI: Results from over 6 years of observation. Annals of the Rheumatic Diseases. 2011;70(8):1382-8.

117. Raynauld JP, Martel-Pelletier J, Dorais M, Haraoui B, Choquette D, Abram F, et al. Total Knee Replacement as a Knee Osteoarthritis Outcome: Predictors Derived from a 4-Year Long-Term Observation following a Randomized Clinical Trial Using Chondroitin Sulfate. Cartilage. 2013;4(3):219-26.

118. Roemer FW, Guermazi A, Javaid MK, Lynch JA, Niu J, Zhang Y, et al. Change in MRI-detected subchondral bone marrow lesions is associated with cartilage loss: the MOST Study. A longitudinal multicentre study of knee osteoarthritis. Ann Rheum Dis. 2009;68(9):1461-5.

119. Roemer FW, Zhang Y, Niu J, Lynch JA, Crema MD, Marra MD, et al. Tibiofemoral joint osteoarthritis: Risk factors for MR-depicted fast cartilage loss over a 30-month period in the multicenter osteoarthritis study. Radiology. 2009;252(3):772-80.

120. Roemer FW, Kwoh CK, Hannon MJ, Green SM, Jakicic JM, Boudreau R, et al. Risk factors for magnetic resonance imaging-detected patellofemoral and tibiofemoral cartilage loss during a six-month period: the joints on glucosamine study. Arthritis Rheum. 2012;64(6):1888-98.

121. Sowers M, Karvonen-Gutierrez CA, Jacobson JA, Jiang Y, Yosef M. Associations of anatomical measures from MRI with radiographically defined knee osteoarthritis score, pain, and physical functioning. Journal of Bone and Joint Surgery - Series A. 2011;93(3):241-51.

122. Tanamas SK, Wluka AE, Pelletier JP, Pelletier JM, Abram F, Berry PA, et al. Bone marrow lesions in people with knee osteoarthritis predict progression of disease and joint replacement: a longitudinal study. Rheumatology. 2010;49(12):2413-9.

123. Tanamas SK, Wluka AE, Pelletier JP, Martel-Pelletier J, Abram F, Wang Y, et al. The association between subchondral bone cysts and tibial cartilage volume and risk of joint replacement in people with knee osteoarthritis: a longitudinal study. Arthritis Res Ther. 2010;12(2):R58.

124. Wildi LM, Raynauld JP, Martel-Pelletier J, Abram F, Dorais M, Pelletier JP. Relationship between bone marrow lesions, cartilage loss and pain in knee osteoarthritis: results from a randomised controlled clinical trial using MRI. Ann Rheum Dis. 2010;69(12):2118-24.

125. Zhang Y, Nevitt M, Niu J, Lewis C, Torner J, Guermazi A, et al. Fluctuation of knee pain and changes in bone marrow lesions, effusions, and synovitis on magnetic resonance imaging. Arthritis Rheum. 2011;63(3):691-9.

126. Aitken D, Khan HI, Ding C, Blizzard L, Pelletier JP, Martel-Pelletier J, et al. Structural predictors of ten year knee cartilage volume loss. Arthritis Rheum. 2013;65:S97-S8.

127. Barr RJ, Gregory JS, Reid DM, Aspden RM, Yoshida K, Hosie G, et al. Predicting OA progression to total hip replacement: can we do better than risk factors alone using active shape modelling as an imaging biomarker? Rheumatology (Oxford). 2012;51(3):562-70.

128. Bennell KL, Creaby MW, Wrigley TV, Hunter DJ. Tibial subchondral trabecular volumetric bone density in medial knee joint osteoarthritis using peripheral quantitative computed tomography technology. Arthritis Rheum. 2008;58(9):2776-85.

129. Bowes MA, Vincent GR, Wolstenholme CB, Conaghan PG. A novel method for bone area measurement provides new insights into osteoarthritis and its progression. Ann Rheum Dis. 2015;74(3):519-25.

130. Doherty M, Courtney P, Doherty S, Jenkins W, Maciewicz RA, Muir K, et al. Nonspherical femoral head shape (pistol grip deformity), neck shaft angle, and risk of hip osteoarthritis: a case-control study. Arthritis Rheum. 2008;58(10):3172-82.

131. Felson DT, Niu J, Guermazi A, Roemer F, Aliabadi P, Clancy M, et al. Correlation of the development of knee pain with enlarging bone marrow lesions on magnetic resonance imaging. Arthritis Rheum. 2007;56(9):2986-92.

132. Hunter DJ, Bowes M, Boudreau RM, Hannon MJ, Kwohx KC. Does bone shape predict the development of incident knee oa? Osteoarthritis Cartilage. 2013;21.

133. Javaid MK, Kiran A, Guermazi A, Kwoh CK, Zaim S, Carbone L, et al. Individual magnetic resonance imaging and radiographic features of knee osteoarthritis in subjects with unilateral knee pain: The health, aging, and body composition study. Arthritis Rheum. 2012;64(10):3246-55.

134. Javaid MK, Lynch JA, Tolstykh I, Guermazi A, Roemer F, Aliabadi P, et al. Pre-radiographic MRI findings are associated with onset of knee symptoms: the most study. Osteoarthritis Cartilage. 2010;18(3):323-8.

135. Neogi T, Bowes MA, Niu JB, De Souza KM, Vincent GR, Goggins J, et al. Magnetic Resonance Imaging-Based Three-Dimensional Bone Shape of the Knee Predicts Onset of Knee Osteoarthritis: Data From the Osteoarthritis Initiative. Arthritis Rheum. 2013;65(8):2048-58.

136. Neogi T, Felson D, Niu J, Lynch J, Nevitt M, Guermazi A, et al. Cartilage loss occurs in the same subregions as subchondral bone attrition: a within-knee subregion-matched approach from the Multicenter Osteoarthritis Study. Arthritis Rheum. 2009;61(11):1539-44.

137. Nicholls AS, Kiran A, Pollard TC, Hart DJ, Arden CP, Spector T, et al. The association between hip morphology parameters and nineteen-year risk of end-stage osteoarthritis of the hip: a nested case-control study. Arthritis Rheum. 2011;63(11):3392-400.

138. Ratzlaff C. RLR, K. Kwoh, M. Hannon, J. Grago, A. Guermazi, F. Roemer, M. Jarraya, D. Hunter, J. Duryea. Quantitative MRI measures of bone marrow lesion volume predict total knee replacement. Osteoarthritis Cartilage. 2014;22:S238-S9.

139. Stahl R, Jain SK, Lutz J, Wyman BT, Le MPM, Vignon E, et al. Osteoarthritis of the knee at 3.0 T: comparison of a quantitative and a semi-quantitative score for the assessment of the extent of cartilage lesion and bone marrow edema pattern in a 24-month longitudinal study. Skeletal Radiology. 2011;40(10):1315-27.

140. Wluka AE, Wang Y, Davis SR, Cicuttini FM. Tibial plateau size is related to grade of joint space narrowing and osteophytes in healthy women and in women with osteoarthritis. Ann Rheum Dis. 2005;64(7):1033-7.

141. Zhao J, Li X, Bolbos RI, Link TM, Majumdar S. Longitudinal assessment of bone marrow edema-like lesions and cartilage degeneration in osteoarthritis using 3 T MR T1rho quantification. Skeletal Radiol. 2010;39(6):523-31.
